# Tough, Ductile, and Strong Hard‐Soft Cementitious Composite Enabled by Multi‐Material Additive Manufacturing

**DOI:** 10.1002/adma.202515461

**Published:** 2026-04-09

**Authors:** Aimane Najmeddine, Shashank Gupta, William Makinen, Zayvinn Lin, Krystal Delnoce, Reza Moini

**Affiliations:** ^1^ Department of Civil and Environmental Engineering Princeton University NJ USA

**Keywords:** finite‐element, fracture toughness, multi‐material additive manufacturing, phase‐field and cohesive‐zone model framework, tough hard‐soft architected cementitious composites

## Abstract

Monolithic cementitious materials lack fracture resistance and are brittle. Advancements in additive manufacturing techniques with cement‐based materials and architected designs have remained limited to the use of a single (cement‐based) constituent. This work charts a new pathway for manufacturing and designing tough, ductile, and strong architected cement‐based composites by proposing a novel multi‐material additive manufacturing (MMAM) technique for the first time, integrated with a coupled experimental‐numerical design approach. The new class of architected cementitious composites (ACC) of mortars and elastomeric constituents (silicone and polyurethane) are exemplified, through a layered hard‐soft architected design, inspired by the microstructure of a sea sponge (glass sponge *Euplectella aspergillum*). The MMAM technique alternates extrusion of hard‐soft composites, enabling systematic control over the geometry and constituents of resulting architected structures.

The results demonstrate layered mortar‐silicone composites achieved up to 3.9‐ and 8.8‐fold enhancements in fracture toughness and up to 11.7‐ and 12.4‐fold enhancements in ductility relative to monolithic 3D‐printed (3DP) and cast mortars, respectively. A coupled large‐deformation phase‐field–cohesive‐zone (PF‐CZM) framework was used to systematically probe soft‐layer thickness and bulk soft‐material properties. Simulations revealed that combining higher‐stiffness soft layers with reduced thickness can yield up to a 24‐fold increase in work‐of‐fracture while recovering the load‐bearing capacity of monolithic mortar.

Guided by numerical predictions, experiments on thin, stiffer polyurethane interlayers validated the numerical predictions and provided additional experimental evidence that the proposed (mortar‐polyurethane) composites achieve 82‐ and 187‐fold higher fracture toughness, and 22.6‐fold higher ductility, relative to 3DP and cast monolithic references while recovering the flexural strength to levels statistically comparable to monolithic mortar. These large gains arise from three synergistic mechanisms: crack arrest/deflection, crack bridging, and discontinuous layerwise crack re‐nucleation, activated by the layered hard‐soft architecture and supported by DIC/AE fracture analyses). The proposed MMAM‐enabled fabrication‐design‐mechanics approach in ACC can unleash entirely new pathways for engineering next‐generation damage‐resilient and multi‐functional concrete structures.

## Graphical abstract

Drawing inspiration from the layered hard‐soft architecture found in sea sponges, this work establishes a new framework for architected cementitious composites (ACC) through multi‐material additive manufacturing (MMAM) process. The integration of mortar and elastomer phases into layered architectures enables synergistic toughening mechanisms, including crack arrest, crack deflection, bridging, and renucleation. Supported by experiments and phase fieldcohesive zone modeling, the study demonstrates that these multi‐material architected composites can overcome the intrinsic brittleness of conventional cementitious materials and achieve a rare combination of toughness, ductility, and strength.

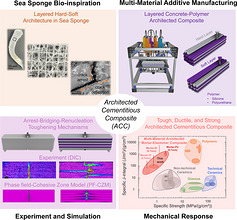

## 1 Introduction

Nature presents one of the most elegant manufacturing processes in producing articulate arrangements [1, 2]. Biological systems, for instance, utilize multiple materials in often rather refined morphologies with contrasting constitutive properties to achieve superior mechanical performance [3]. These complex multi‐materials are often arranged into purposeful architected arrangements that are not only geometrically intricate, but also essential for their functional characteristics [4, 5]. Biological materials such as nacre [6], bone [7], dentin [8], and sponge skeletons [9], for example, demonstrate strategic arrangements of ‘hard’ and ‘soft’ components across multiple length scales to achieve remarkable combinations of toughness and strength [1, 9, 10, 11, 12, 13, 14, 15]. More specifically, these natural composites typically feature hard, brittle mineral phases interspersed with compliant organic interlayers that enable energy dissipation through controlled deformation and damage delocalization mechanisms, resulting in materials that are simultaneously ductile, damage‐tolerant, and strong [1, 6, 10, 12, 16].

Human‐made materials, particularly concrete ‐ the world's most widely used commodity and structural material [17] ‐ suffer from inherent brittleness [18, 19] and low fracture toughness [20]. This brittleness and low fracture toughness are characteristic of synthetic structural materials, including glass [21, 22], technical ceramics [23], and non‐technical ceramics [24], of which cement paste, mortar, and concrete are prime examples. Despite these well‐known limitations, efforts to develop tough and ductile concrete have remained largely conventional over recent decades, primarily relying on fiber reinforcement within cementitious matrices [25, 26] through traditional casting methods.

The advent of concrete additive manufacturing and automated robotic extrusion processes [27, 28] has enabled significant advancements in material architecture design [28, 29]. However, current extrusion‐based additive manufacturing processes across all scales ‐ from desktop 3D‐printing at millimeter‐to‐centimeter scales [30, 31] to benchtop robotic fabrication at centimeter‐to‐meter scales [32, 33, 34], and large industrial gantry systems spanning several meters [35, 36, 37, 38, 39, 40, 41] ‐ remain fundamentally limited to single‐material applications or exclusively with cement‐based constituents (i.e., in cement paste, mortar, or concrete) [42, 43]. Consequently, the development of bio‐inspired tough and ductile composites composed of multiple materials has remained a significant challenge using current concrete fabrication technologies. Engineering the mechanics of cementitious materials to transition from brittle and weak to tough and strong behavior, inspired by the dissimilar constituents found in natural arrangements, requires fundamental advancements in manufacturing processes. In this paper, we present a novel multi‐material additive manufacturing (MMAM) process that significantly expands bio‐inspired design freedom for engineering tough, strong, and ductile architected cement‐based composites beyond conventional monolithic structures. The MMAM process enables a new way to directly address the inherent brittleness and limited ductility characteristic of cementitious materials like concrete, without sacrificing the strength.

In broader additive manufacturing domains, multi‐material approaches have recently gained significant traction [44, 45, 46]. Various systems incorporating polymer‐polymer [47, 48, 49], ceramic–metal [50, 51, 52], concrete‐metal [53], and other hard‐soft material combinations [54, 55] have been developed to achieve improved characteristics, including enhanced fracture toughness, wear resistance, or adaptive compliance. Nacre‐like and layered hard‐soft architectures have also been explored at smaller scales in glass‐polymer and polymer‐polymer systems, including centrifuged or infiltrated nacre analogues and multi‐material printed polymer laminates [56, 57], with a broader context provided by reviews of bio‐inspired hard‐soft composites [58]. Despite these advances, there is, to our knowledge, no prior work that (i) realizes layered hard‐soft architectures in *cementitious* materials using a multi‐material extrusion‐based process, (ii) integrates a compliant elastomeric layer directly with a 3DP mortar configuration at meso‐scale, and (iii) systematically couples experiments with a computational fracture‐mechanics framework to map how geometric and material design factors (e.g., soft layer thickness and soft layer stiffness) influence fracture toughness, ductility, and strength, and to iteratively inform subsequent experimental designs aimed at discovering even better‐performing composites. In particular, existing nacre‐like and polymer‐polymer systems [56, 57] do not address the fabrication or fracture mechanics of layered mortar‐elastomer composites, nor do they provide design guidance for multi‐material concrete additive manufacturing. This represents a significant gap, especially given the potential to overcome the fundamental brittleness and poor fracture resistance that characterize single‐constituent cementitious systems.

**FIGURE 1:**
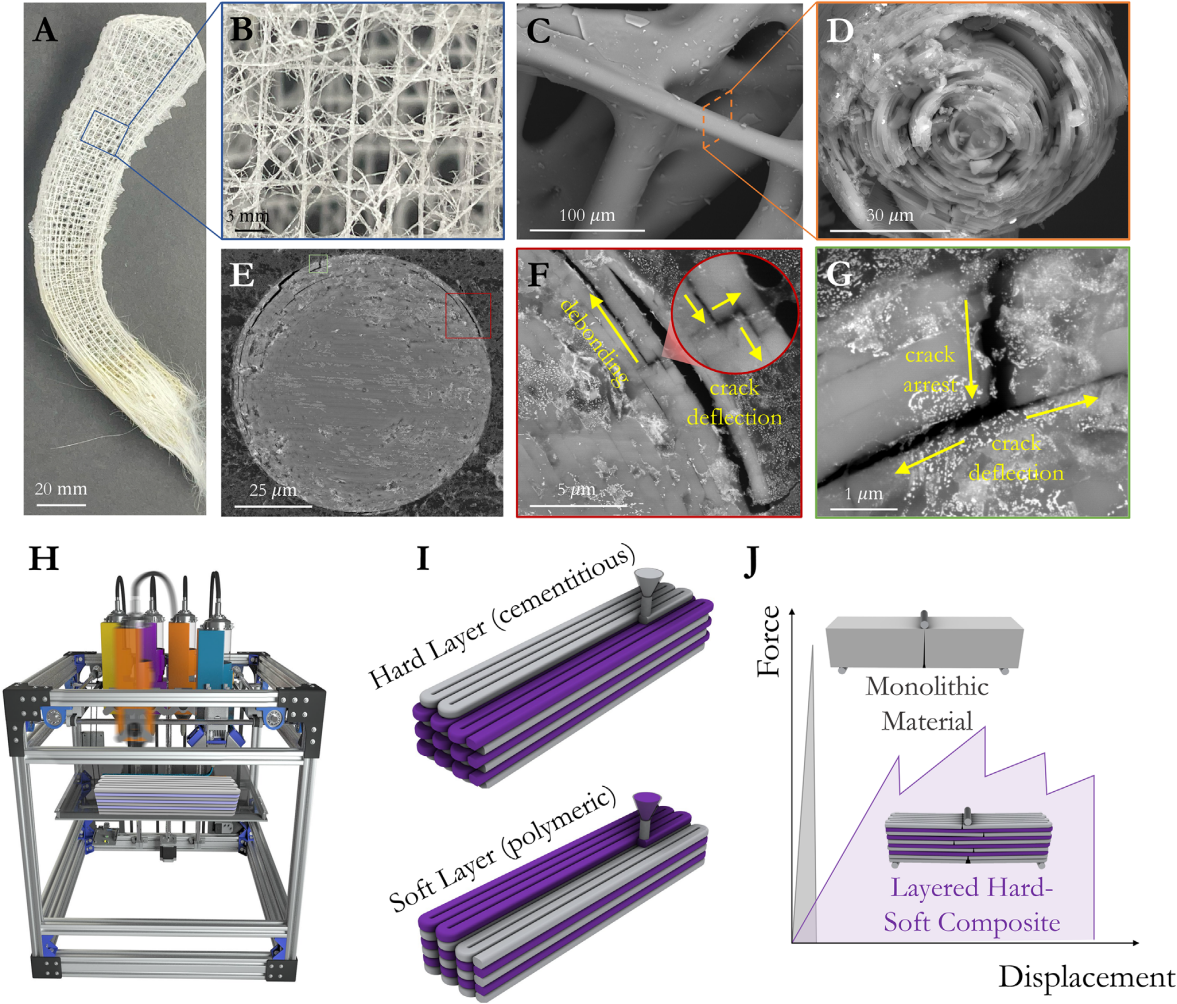
(A–G) Structural characteristics of *Euplectella aspergillum* (EA) glass sea‐sponge. (A) Photograph of the skeleton of EA from the spicules anchored in the soft sediments of the sea floor (bottom) to the cap (top). (B) Close‐up of the cage structure (from A) showing a lattice of orthogonal and diagonal struts. (C) Scanning electron microscopy (SEM) image of intersecting struts (bundles of spicules). (D) SEM cross‐sectional image of a single spicule displaying concentric layered ring‐like architecture with alternating mineral (siliceous) and organic (proteinaceous) phases. (E) SEM image of a fractured spicule surface (prepared using Focused Ion Beam (FIB) milling) demonstrating crack propagation mechanisms in the layered architecture. (F) Magnified view of the top right region of E illustrating crack deflection and debonding between the hard siliceous layers, preventing straight crack propagation. (G) Magnified view of the top center region of E demonstrating crack arrest at the hard‐soft (siliceous‐proteinaceous) interface, followed by crack deflection within the multi‐material layered architecture. (H–J) Multi‐material additive manufacturing of tough and ductile layered hard‐soft (cementitious‐elastomer) assembly. (H) Custom multi‐material additive manufacturing platform designed with multiple extruders (interchangeable toolheads) capable of depositing both hard (cementitious suspension) and soft (elastomeric) materials consecutively in programmable patterns (Figures S1 and S2). (I) Schematic of consecutive hard‐soft layerwise deposition process for the fabrication of the proposed architected multi‐material assembly (also broadly defined as architected cementitious composites). (J) Schematic force‐displacement curves of the anticipated enhanced mechanical performance of the proposed hard‐soft (cementitious‐elastomer) architected multi‐material (purple) compared to monolithic cast and additively manufactured counterparts (gray), hypothesized to demonstrate significantly improved fracture toughness and ductility, exhibiting a transition from catastrophic failure to graceful failure.

Here, we introduce a MMAM technique that enables the fabrication of layered hard‐soft architected multi‐material composites, also defined by the authors as architected cementitious composites (ACC), that combine two major additional degrees of freedom: geometry and material constitutive properties. By drawing inspiration from *Euplectella aspergillum* (EA) (Figure 1A–G), commonly known as the Venus flower basket, a deep‐sea hexactinellid sponge inhabiting the Western Pacific at depths of 100‐1000 meters [59], the utility of the proposed MMAM is exemplified. In such extreme environments, the sponge must withstand hydrostatic pressures and mechanical stresses that would otherwise shatter conventional brittle glass constituents [60]. The species contains spicules (silica fibers) whose function varies from predation deterrence to providing mechanical rigidity for the skeleton of the sponge [61].

The EA's cylindrical skeleton (Figure 1A) forms a diagonally‐reinforced rectangular lattice (Figure 1B) constructed from bundles of fused spicules (Figure 1C). These spicules are laminated skeletal elements consisting of a central proteinaceous axial filament surrounded by alternating concentric domains of consolidated silica nanoparticles (∼99% volume fraction [62]) and organic interlayers (5–10 nm thick) (Figure 1D) [9, 62, 63, 64, 65]. The concentric arrangement of layers within each spicule protects the central core through predominantly three toughening mechanisms (Figure 1E), with the organic layers promoting crack arrest through crack deflection and partial debonding (Figure 1F,G). These toughening mechanisms arise from the layered architecture, which has been shown to improve fracture toughness and failure strain of spicules [63, 64, 65, 66, 67]. More generally, recent studies on bio‐inspired and composite architected materials have demonstrated that properly designed soft or compliant interlayers can trigger early crack deflection/arrest, promote stepwise fracture, and in some cases improve damage resilience, underscoring the broader mechanistic basis for the layered strategy pursued here.

Inspired by the layered architecture of the EA species, we develop a multi‐material extrusion‐based additive manufacturing approach to enable the engineering of tough cementitious‐elastomeric layered multi‐materials. The multi‐material additive manufacturing (MMAM) process leverages a custom multi‐material 3D printer design (Figure 1H) capable of depositing both hard and soft materials in a programmable fashion for a given design arrangement (e.g., layer‐by‐layer) (Figure S1). Using this technique, two or more materials are sequentially or simultaneously deposited in a layer‐wise manner (Movies S1 and S2), enabling purposeful architectures of mortar‐elastomer multi‐material assemblies. We implement the MMAM process using a tool‐changing system (Figure 1H; Figure S1–S4) that parks and picks various toolheads associated with multiple materials. Importantly, all composite specimens analyzed throughout this work are 3DP, as layered mortar‐elastomer beams with two bottom mortar layers followed by alternating mortar and elastomer layers, fabricated using this custom MMAM platform (see Figure S1–S4 for full processing details).

We hypothesize that using the MMAM technique and incorporating soft elastomeric interlayers within a layered cementitious material (Figure 1I) significantly promotes damage delocalization, enhancing fracture toughness and ductility compared to monolithic cementitious counterparts (Figure 1J). Specifically, we anticipate that engaging large deformations in the soft materials will activate new toughening mechanisms in the layered composite, enabling crack arrest and graceful fracture while preventing the catastrophic failure common in monolithic cementitious materials (as conceptually illustrated in Figure 1J). These hypotheses are examined through rigorous fracture mechanics experiments followed by finite‐element simulations using a recently developed numerical phase‐field and cohesive zone model (PF‐CZM) framework [68, 69] to understand the underlying toughening mechanisms and to refine the proposed hard‐soft material design compared to monolithic cast and additively manufactured counterparts. The numerical findings are then validated through an extended experimental protocol, further substantiating the proposed MMAM‐enabled pathway for designing architected mortar‐elastomer composites that simultaneously enhance fracture toughness, ductility, and load‐bearing capacity beyond monolithic cementitious counterparts.

## 2 Results

Unless otherwise noted, the results presented in this section concern 3D‐printed (3DP), layered mortar‐elastomer beams fabricated using the custom multi‐material extrusion‐based printing platform introduced in Figure 1H and detailed in Figures S1–S4. The architected specimens consist of two bottom mortar layers followed by alternating mortar and soft elastomer layers (initially silicone and, in later designs, polyurethane), forming 110×27×28 *mm* beams with a layered hard‐soft architecture. Monolithic cast and 3DP mortar beams of identical overall dimensions serve as reference systems. Using this common specimen configuration, we first quantify fracture toughness and ductility enhancements over monolithic systems experimentally, then employ a coupled phase‐field–cohesive zone modeling framework to probe the influence of soft‐layer thickness and stiffness, and finally validate the numerically identified designs (including polyurethane‐based alternatives to silicone interlayers) through additional experiments and acoustic‐emission–based fracture analysis.

### 2.1 Experimental Evaluation of Fracture Responses

**FIGURE 2:**
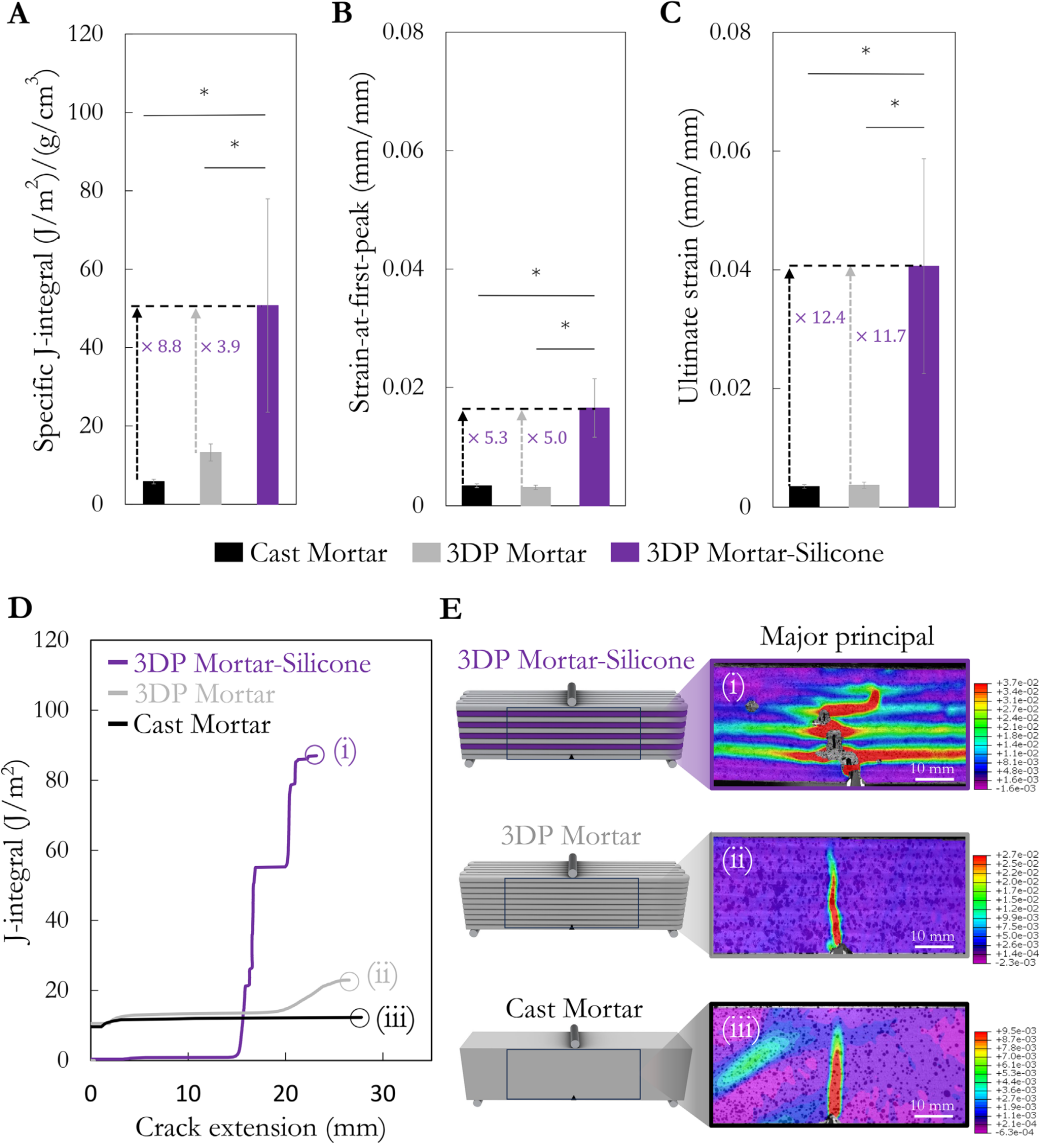
Mechanical response and characteristic toughening mechanisms of 3D‐printed (3DP) architected hard‐soft mortar‐silicone multi‐material assemblies compared to 3DP and cast monolithic mortar counterparts. (A) Specific J‐integral (i.e., strain energy release rate normalized by density) (measured using single‐edge notch bend test) for 3DP mortar‐silicone multi‐material versus 3DP and cast mortar, demonstrating statistically significant enhancement of fracture toughness (*p<0.05). B. Strain‐at‐first‐crack (measured using unnotched three‐point bending test), demonstrating delayed crack initiation in the multi‐material case versus both monolithic counterparts. (C) Ultimate strain capacity (measured using unnotched three‐point bending test) for 3DP mortar‐silicone multi‐material versus 3DP and cast mortar, demonstrating statistically significant enhancement of ductility (*p<0.05). (D) J‐integral versus crack extension curves revealing a substantially rising resistance‐curve (R‐curve) in the 3DP multi‐material (sample i) compared to near‐flat responses in 3DP mortar (sample ii) and cast mortar (sample iii). (E) Digital image correlation (DIC) of the major principal strain contours illustrating contrasting fracture responses: localized strain in cast mortar (sample iii), moderate strain distribution in 3DP mortar (sample ii), and extensive strain distribution with tortuous crack path in the 3DP mortar‐silicone multi‐material (sample i).

In this section, the elastomer used in the fabrication and mechanical testing of the hard‐soft mortar‐elastomer architected composites is a silicone elastomer (see Figure S7 for the corresponding mechanical characteristics). Fracture toughness of the three configurations, i.e., 3D‐printed (3DP) mortar‐silicone architected multi‐material assembly, 3DP monolithic mortar, and cast monolithic mortar, was evaluated through J‐integral (path‐independent strain energy release rate) calculations [70] and digital image correlation (DIC) analysis based on the single‐edge notch bend test performed on all samples (Figure 2). Details of the J‐integral calculations are provided in Supporting Information. Strain‐at‐first‐crack and ultimate strain were measured using the unnotched three‐point bending test (details are provided in the Experimental Section). For the 3DP mortar‐silicone multi‐material systems, architected assemblies were printed with silicone and mortar layers of equal thickness ($t_{e}=2.7\;\text{mm}$ each, i.e., silicone‐to‐mortar thickness ratio of r=1), resulting in a total beam depth of 27mm. The multi‐material 3DP configuration consists of two mortar layers at the bottom (including the layer containing the pre‐existing notch), followed by alternating mortar and silicone layers. Additional details about the fabrication and toolpath generation for the 3DP multi‐material and 3DP mortar configurations are provided in the Experimental Section and Figures S1–S4.

Results demonstrate a significant contrast between the mechanical properties and fracture response of the 3DP multi‐materials relative to the 3DP and cast mortar configurations. Figure 2A illustrates the enhancements in the fracture‐toughness quantified in terms of the density‐normalized specific J‐integral. The figure demonstrates that the 3DP mortar‐silicone multi‐material assembly exhibits a statistically significantly higher specific J‐integral (51.0±27 ${J/m}^{2}$/(${g/\mathrm{cm}}^{3}$)) compared to both the 3DP mortar (13.2±2.2 ${J/m}^{2}$/(${g/\mathrm{cm}}^{3}$)) and cast mortar (5.80±0.6 ${J/m}^{2}$/(${g/\mathrm{cm}}^{3}$)), representing a 3.9‐fold and 8.8‐fold increase, respectively.

The strain‐at‐first‐crack (Figure 2B) remains relatively similar between the cast mortar (0.0033±0.001mm/mm) and 3DP mortar (0.0031±0.001mm/mm); however, it increases significantly in the 3DP mortar‐silicone multi‐material system (0.0165±0.005mm/mm), representing 5‐fold and 5.3‐fold increases compared to both monolithic systems, respectively. This enhancement suggests that in the multi‐material case, crack initiation is effectively delayed by accommodating higher strains (i.e., larger deformation) at the onset of crack propagation from the notch due to the presence of the soft silicone interlayers.

Similarly, the ultimate strain capacity (i.e., ductility) (Figure 2C) of the 3DP mortar‐silicone multi‐material (0.041±0.019mm/mm) increases substantially, reaching approximately 12.4‐fold and 11.7‐fold enhancements compared to cast mortar (0.0033±0.001mm/mm) and 3DP mortar (0.0035±0.001mm/mm), indicating significantly higher ductility in the multi‐material system.

Resistance curve (R‐curve) represents the J‐integral versus crack extension (Figure 2D), providing insights into the fracture response during crack propagation, revealing the presence of any crack‐microstructure interactions and associated toughening mechanisms in the three configurations. Both monolithic cases, the cast mortar (black) and 3DP mortar (gray), exhibit a nearly flat R‐curve, characteristic of brittle materials with minimal resistance to crack propagation upon initiation, plateauing at approximately 10.4 and 22.9 ${J/m}^{2}$, respectively. In contrast, the 3DP mortar‐silicone multi‐material case (purple) demonstrates a distinctly rising R‐curve behavior, with the J‐integral value increasing substantially with crack extension, in a staggered manner, reaching 87.1 ${J/m}^{2}$. This represents a nearly 8.4‐fold increase compared to cast mortar and a 3.8‐fold increase compared to reference 3DP mortar. The rising R‐curve indicates the activation of multiple toughening mechanisms throughout the crack propagation (Figure 2D).

Figure 2E illustrates DIC contours of the major principal strain profiles at the end of the crack extension, i.e., points (i), (ii), (iii) in the R‐curve (Figure 2D) for 3DP mortar‐silicone, 3DP mortar, and cast mortar, respectively. In the cast mortar case (Figure 2E, black), strain localization is confined to a narrow band with limited strain values (maximum major principal strain of approximately $0.95\times 10^{-2}$), indicating a single dominant crack with minimal energy dissipation upon propagation. The 3DP mortar (Figure 2E, gray) shows slightly higher strain values (maximum principal strain of approximately $2.7\times 10^{-2}$) compared to cast mortar. In contrast to localized damage in the monolithic cases, the 3DP mortar‐silicone multi‐material assembly (Figure 2E, purple) displays a fundamentally different fracture profile, with extensive damage delocalization illustrated by the high strain distribution across a broad area (i.e., maximum principal strain reaching $3.7\times 10^{-2}$) and with tortuosity in the crack path indicative of several toughening mechanisms.

The relatively large scatter in the J‐integral values demonstrated in Figure 2A,D for the mortar‐silicone composite case is expected due to the J‐integral being a derived quantity (i.e., calculated from the effective modulus obtained from the load‐CMOD compliance and from the load‐CMOD response up to the first peak). Variability in the effective modulus propagates directly into the computed J‐integral values. Such scatter is expected for 3D‐printed layered mortar‐elastomer composite case, which are multi‐material systems with several interfaces and therefore contain more intrinsic sources of variation in terms of damage mechanisms and compliance compared to monolithic counterparts. In particular, the global compliance is highly sensitive to the soft interlayer's geometry; thus, small differences in elastomer thickness or interfacial quality can significantly affect the overall stiffness and resulting computed J‐integral. In addition, fracture in these multi‐material assemblies involves multiple competing mechanisms, including crack initiation, arrest/deflection at the elastomer layer, bridging, and re‐nucleation, making the response inherently stochastic in that the type and order of dissipation can lead to larger variability in fracture properties and ultimate strain (strain at failure). Therefore, the observed error bars in the 3DP mortar‐silicone case reflect the intrinsic variability of the architected composite system. A more extended discussion about the variability of fracture and ductility properties is provided in the Supporting Information. Nevertheless, despite the large scatter, the quantitative differences observed between the mortar‐silicone architected composites and the monolithic references remain large and statistically significant, as indicated by the one‐tailed F‐ and T‐tests reported in Figure 2A.

**FIGURE 3:**
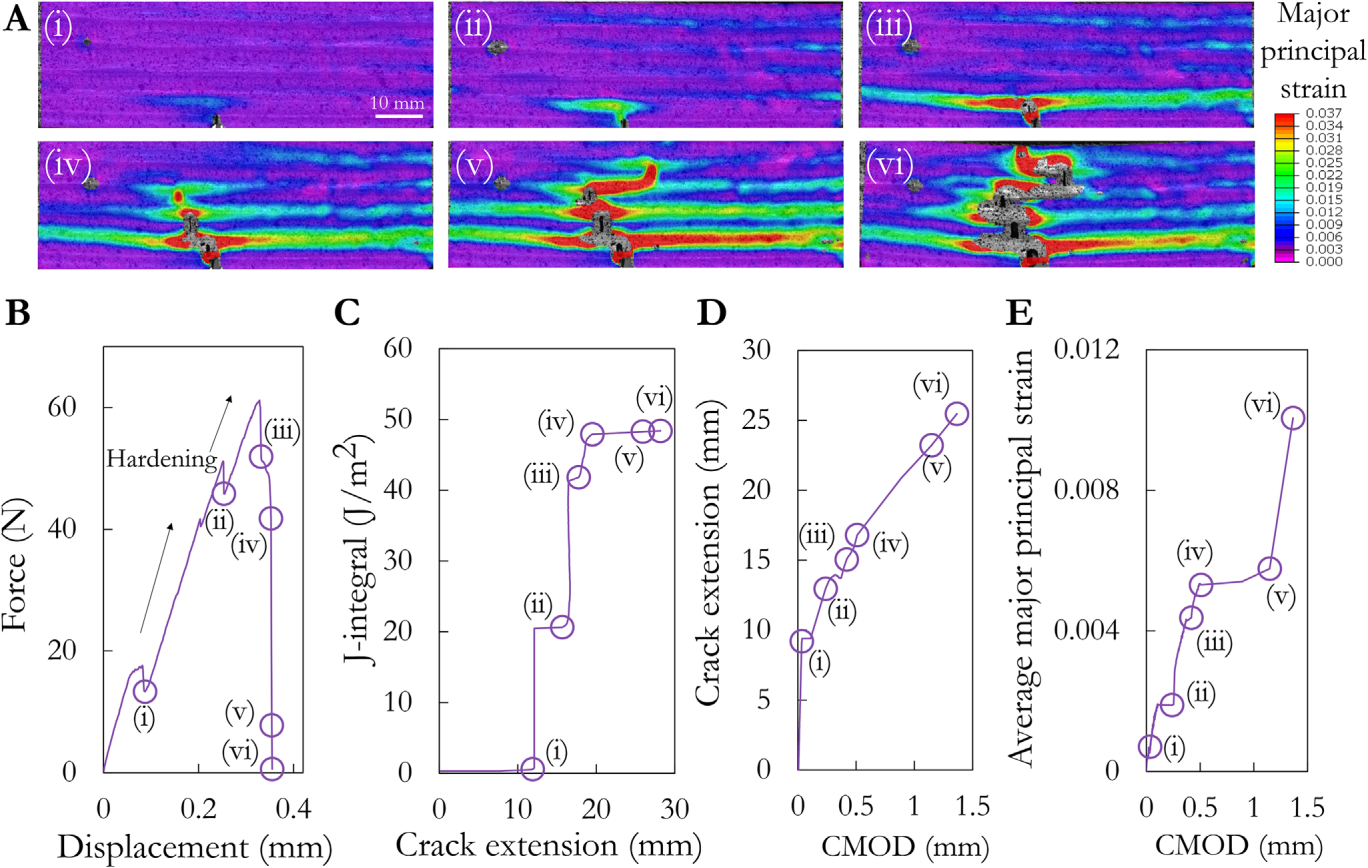
Crack propagation analysis for a representative 3D‐printed (3DP) mortar‐silicone multi‐material assembly using digital image correlation (DIC). (A) DIC demonstrating the major principal strain contours during the multi‐stage crack propagation (i‐vi), revealing a multi‐stage tortuous fracture involving distributed damage through several toughening mechanisms (crack arrest, deflection, bridging, and re‐nucleation) across multiple silicone and mortar layers. (B) Force‐displacement curve highlighting multi‐stage non‐linear fracture behavior with controlled post‐peak hardening and softening at marked measurement points (i‐vi). (C) J‐integral evolution demonstrating substantial fracture toughness with rising R‐curve behavior, reaching a maximum of 48.4 ${J/m}^{2}$ (point vi) during crack propagation. (D) Crack extension versus crack mouth opening displacement (CMOD) relationship indicating stable crack growth over 1.4mm opening. (E) Full‐beam average major principal strain versus CMOD showing substantially high ductility. Scale bar: 10mm.

Figure 3A illustrates the evolution of the major principal strain in the 3D‐printed (3DP) mortar‐silicone multi‐material system throughout the beam depth. The crack initiates at the pre‐existing notch in the bottommost mortar layer and propagates upward through the mortar‐mortar interface into the adjacent mortar layer (points i‐ii in Figure 3A). Upon reaching the first mortar‐silicone interface, the crack arrests. As loading continues, the silicone layer accommodates deformation through stretching without allowing crack propagation. When the subsequent mortar layer accumulates sufficient strain, the crack re‐nucleates from a new location near the beam center (point iii). This mechanism repeats throughout the beam depth (points iv, v, vi), resulting in the tortuous crack profile shown in Figure 3A. The crack develops a staggered pattern that deviates from the mid‐span after initial propagation. The repeated cycle of crack arrest and its re‐nucleation consumes energy, which is one way the beam increases its resistance to fracture, as was illustrated in the purple curve of Figure 2D.

The DIC analysis reveals that this multi‐stage crack propagation involves several steps: (1) fracture initiation in the hard mortar layer, (2) crack arrest at mortar‐silicone interfaces, (3) extensive deformation within the silicone layers leading to subsequent hardening after each mortar fracture event, (4) crack bridging by the soft‐layers, and (5) crack re‐nucleation after deflection at subsequent mortar layers. The presence of soft layers therefore enables graceful, multi‐stage, and stable crack propagation, in contrast to monolithic cast and 3DP mortar, ultimately enhancing both fracture toughness and ductility as documented in Figure 2.

The force‐displacement curve of the 3DP mortar‐silicone multi‐material (Figure 3B) demonstrates the non‐brittle multi‐stage fracture response identified qualitatively in the DIC analysis ‐ namely, initial elastic deformation, progressive energy dissipation due to damage delocalization and multi‐stage cracking (as discussed through Figure 3A), and controlled post‐peak softening. The response exhibits multiple staggered load drops (points i through vi), each corresponding to the fracture of an individual mortar layer. These discrete load drops indicate that each mortar layer fails independently rather than catastrophically, with the silicone interlayers maintaining load transfer between successive fracture events.

The corresponding J‐integral evolution (Figure 3C) quantifies the energy dissipation during fracture, with values increasing from crack initiation at point i to approximately 20 ${J/m}^{2}$ at point ii, where the second mortar layer fractures, and ultimately reaching 48.4 ${J/m}^{2}$ through points iii, v, and vi, where the last three mortar layers fracture.

The crack extension versus CMOD (Crack Mouth Opening Displacement) plot exhibits a gradual increase in crack length with increasing CMOD (Figure 3D), indicating stable crack growth from the first to the last fracture in the hard layers (points i‐vi). The crack extends to approximately 26mm at a large CMOD value of 1.4mm. The crack extension‐CMOD plot demonstrates the multi‐material's capacity to sustain substantial deformation during crack propagation while maintaining structural integrity.

The full‐beam average principal strain versus CMOD plot (Figure 3E) further elucidates the large deformation characteristics of the multi‐material assembly, with strain values progressively increasing from 0.001 at point i to 0.01 at point vi, induced by the extensive deformation experienced by all four silicone layers as visually documented in Figure 3A. This high strain capacity ‐ approximately an order of magnitude higher than conventional mortars (∼0.001 strain at failure [71, 72]) ‐ directly results from the large deformation capability of silicone interlayers.

**FIGURE 4:**
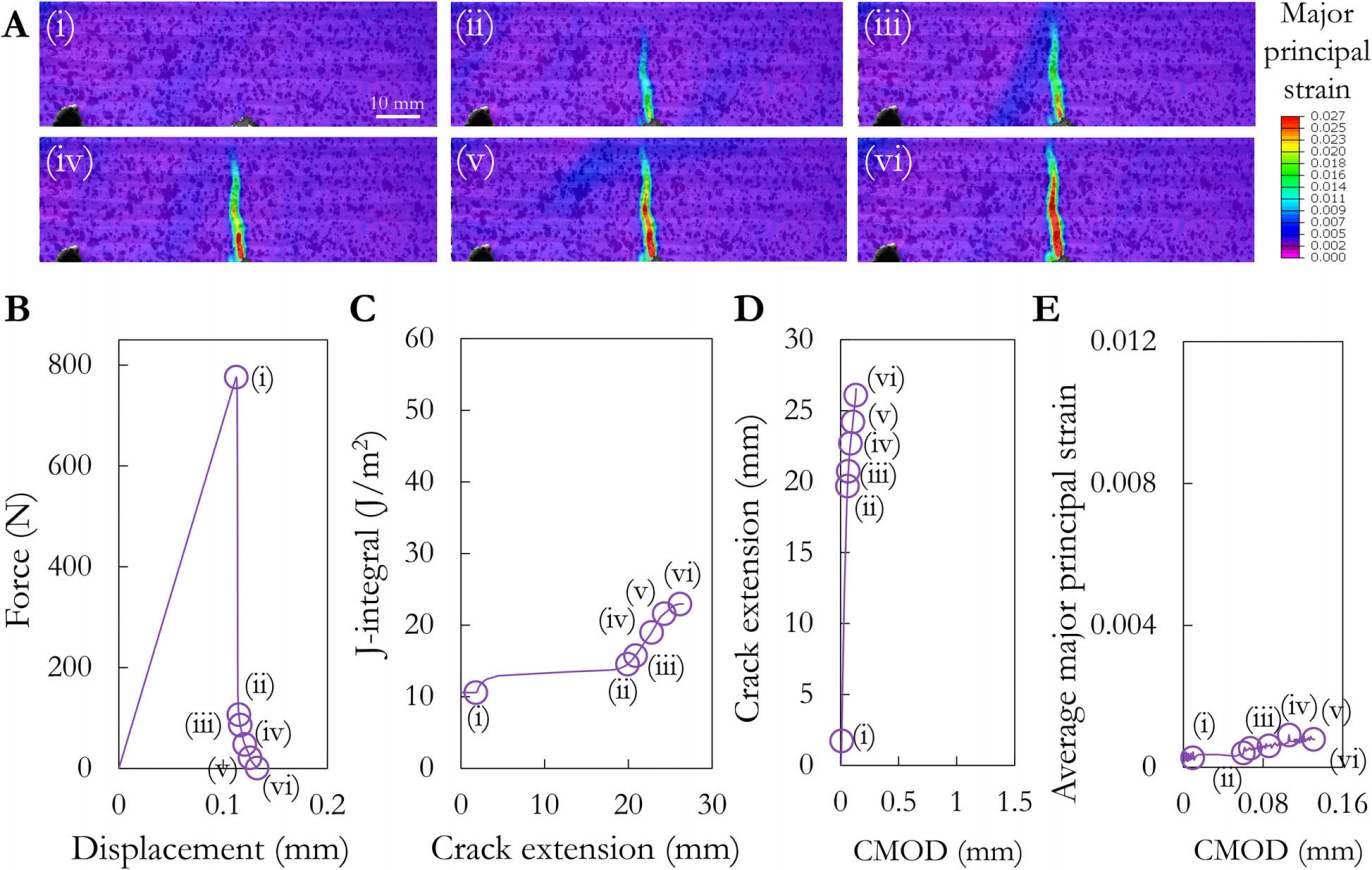
Crack propagation analysis for the reference monolithic 3D‐printed (3DP) mortar using digital image correlation (DIC). (A) DIC demonstrating the major principal strain contours during the abrupt crack propagation (i‐vi), illustrating highly localized strain along a single, straight crack path involving minimal damage distribution in the hard‐hard filaments, demonstrating characteristic brittle fracture in 3DP mortar. (B) Force‐displacement curve showing abrupt linear elastic fracture behavior followed by limited post‐peak softening and rapid deterioration. (C) J‐integral evolution demonstrating limited fracture toughness reaching only 22 ${J/m}^{2}$ (point vi) due to the absence of major toughening mechanisms and crack‐material interactions. (D) Crack extension versus crack mouth opening displacement (CMOD) demonstrating rapid crack propagation at a very small CMOD value of only 0.13mm. (E) Full‐beam average major principal strain measurements confirming limited strain capacity with maximum values of only 0.0015. Scale bar: 10mm.

In contrast, the monolithic 3D‐printed (3DP) mortar exhibits a fundamentally different fracture response. The DIC strain contours (Figure 4A) illustrate a rather highly localized strain field along a single, relatively straight crack path ‐ compared to the multi‐material case (Figure 3A) ‐ with minimal spread of damage into the surrounding material.

The force‐displacement curve (Figure 4B) demonstrates a linear elastic response up to the peak load (point i), followed by a brittle load drop and subsequent rapid and complete loss of load‐bearing capacity (points ii through vi). This response is expected in rectilinear 3DP brittle and quasi‐brittle cement‐based materials, indicative of the material's inherent brittleness, in contrast to the non‐brittle, graceful response of the hard‐soft multi‐materials illustrated in Figure 3B.

The J‐integral evolution (Figure 4C) displays a modest increase from approximately 10 ${J/m}^{2}$ at crack initiation (point i) to a maximum of only 22.29 ${J/m}^{2}$ at points v and vi due to the subtle softening tail of the load‐displacement response. This limited fracture resistance contrasts sharply with the R‐curve reported for the multi‐material case in Figure 3C.

The crack extension vs CMOD plot (Figure 4D) reveals a rapid crack propagation at very small CMOD values, with the crack extending to approximately 26mm at point vi with a CMOD of only 0.13mm ‐ approximately one‐eleventh of the ultimate CMOD value found for the multi‐material (1.4mm). This rapid crack growth at minimal deformation further underscores the brittle nature of 3DP mortar and the limited underlying microstructural toughening mechanism.

The average major principal strain measurements (Figure 4E) confirm the limited strain capacity of the monolithic material, with maximum values reaching only 0.0008 at point vi: approximately one‐thirteenth of the strain achieved in the multi‐material case (0.01).

The experiments reveal several toughening mechanisms, activated sequentially, that are unique to the proposed bio‐inspired mortar‐silicone multi‐materials and absent in both monolithic counterparts:

1. Crack arrest (and deflection) through the mortar‐silicone multi‐material layers, similar to those found in the organic‐inorganic layered EA glass sea‐sponge, induced by the presence of soft material, leading to a tortuous crack path that increases the effective fracture surface area and energy dissipation.
2. Crack bridging between the mortar layers by the soft layers, which provides substantial energy absorption through large deformation upon each hard mortar fracture event.
3. Crack re‐nucleation within subsequent mortar layers, leading to a multi‐stage crack propagation mechanism. When the crack deflects along the interface before re‐nucleating, it must overcome additional resistance and create new fracture surfaces, contributing to the overall toughening effect measured through the J‐integral.

The toughening mechanisms identified here are consistent with prior observations in layered and bio‐inspired hard‐soft composites. In particular, crack arrest/deflection, crack bridging, and crack re‐nucleation or stepwise crack advance have all been reported as effective routes for suppressing catastrophic fracture and promoting progressive failure in architected composite systems [16, 66, 73, 74, 75, 76, 77]. Similar to the present study, Wiener et al. have demonstrated that compliant soft interlayers in talc‐reinforced polypropylene multilayer composites can arrest propagating cracks and enhance fracture toughness, with the extent of the improvement governed by interlayer compliance, interlayer thickness, and the number of interlayers [74]. Greenfeld and Wagner have similarly reported in sponge‐inspired layered architectures, motivated by the siliceous skeleton of *Euplectella aspergillum*, that crack deflection/delamination and repetitive propagation‐deflection sequences suppress catastrophic crack advance and improve structural resilience [75]. Likewise, Jia et al. have demonstrated, in 3D‐printed biomimetic architected beams inspired by nacre‐, conch‐, and fish‐scale‐like motifs, that structural design can activate multiple fracture processes, including crack deflection and crack bridging, thereby improving the strength‐toughness balance [76]. Additional studies on nacre‐inspired and architected cementitious composites have similarly identified tortuous crack propagation, interlayer deformation, and crack bridging as key contributors to rising crack‐growth resistance [16, 56]. In the present study, using a mortar‐elastomer system enabled by MMAM, these mechanisms are expressed synergistically within the same layered cementitious composite, where crack arrest/deflection at the mortar‐elastomer interface, large‐deformation bridging by the soft interlayer, and crack re‐nucleation in the subsequent mortar layer occur as a coupled sequence during fracture. Therefore, the present results place the observed behavior within the broader framework of bio‐inspired layered toughening, while further showing that these three mechanisms can operate in concert in a single additively manufactured cementitious architecture.

The experimental findings demonstrate that soft material presence is critical for enabling these toughening mechanisms. Consequently, both the soft‐layer geometry (thickness) and material properties (stiffness) relative to the hard layer are expected to be central parameters for engineering the mechanical properties of these multi‐materials. To explore this relationship, the soft‐to‐hard thickness ratio and the soft‐to‐hard stiffness ratio between the layers are investigated.

More specifically, the soft‐to‐hard thickness ratio governs the balance between compliance capacity and toughening activation. We hypothesize that thinner soft layers can maintain the three key toughening mechanisms (arrest, bridging, and re‐nucleation) while simultaneously enhancing fracture toughness and load‐bearing capacity.

Equally important, the substantial elastic mismatch, with silicone exhibiting a modulus approximately five orders of magnitude lower than mortar, enables extensive energy dissipation through large deformations in the soft phase without fracture. This mismatch is essential for crack bridging because, without sufficient compliance contrast, the soft layer cannot accommodate the strain energy that would otherwise drive abrupt crack propagation through the brittle hard layer. This principle is evidenced by the absence of bridging in monolithic 3DP mortar, where no elastic mismatch between layers exists. Soft compliant layers overcome the inherent brittleness of ceramics by providing a ‘cushion effect’ that enables progressive failure mechanisms. We hypothesize that soft layers with higher stiffness can enhance both toughness and load‐bearing capacity of multi‐materials.

The potential for synergistic optimization of layer thickness ratio and elastic mismatch can create conditions necessary for maximizing the arrest, bridging, and re‐nucleation mechanisms. To systematically investigate these geometric and material interdependencies and establish design guidelines for engineering mutually exclusive properties (i.e., load‐bearing capacity and toughness) in architected multi‐materials, we next employ numerical modeling to parametrically explore soft‐to‐hard thickness ratio and soft‐to‐hard stiffness ratio effects using the phase‐field cohesive zone model (PF‐CZM) framework.

### 2.2 Numerical Evaluation of Fracture Responses

To elucidate the fundamental mechanisms governing fracture behavior in architected multi‐materials of hard‐soft layers, a comprehensive finite‐element investigation is conducted, examining two critical design parameters: (1) geometry: soft‐to‐hard layer thickness ratio and (2) property: soft‐to‐hard material stiffness ratio. The numerical simulations employ a state‐of‐the‐art constitutive framework that integrates a large‐deformation phase‐field approach for modeling bulk fracture [78, 79] with the Park‐Paulino‐Roesler Cohesive‐Zone‐Model (PPR‐CZM) [80], capable of simulating crack propagation in hard‐soft multi‐materials with interfaces [68, 69, 81]. This unified phase‐field PPR framework is detailed in Figure S5, and is implemented through a user‐element subroutine for plane‐strain 2D problems within the finite‐element software ABAQUS/STANDARD [82]. The numerical simulations enable the probing of broader design parameters beyond experiments to better understand and engineer the mechanics of the proposed hard‐soft multi‐materials through informed selection of the geometric configuration and the relative properties of the materials. The hard material properties and the number of layers have been kept constant throughout the simulations, parametrically altering only the soft material thickness and soft material stiffness.

Note that to simulate crack propagation in the single‐edge notch bend (SENB) test, the previously developed unified phase‐field CZM framework is further extended in this study to incorporate tension‐compression asymmetry within both bulk materials (i.e., hard and soft domains) that is present under bending and necessary for carrying out the simulations (Supporting Information). Here, the numerical study represents the soft constituent with the mechanical properties typical of rubbery and compliant materials (i.e., elastomeric) and the hard constituent with the properties typical of non‐technical brittle ceramics (i.e., cementitious mortar).

#### 2.2.1 Effect of Soft Material Geometry

To investigate the effect of the elastomer‐to‐mortar thickness ratio, r, on the overall fracture response, finite‐element simulations were conducted for three elastomer layer thickness configurations: $t_{e}=2.7\;\text{mm}$ (r=1, matching experimental geometric design), $t_{e}=1.7\;\text{mm}$ (r=0.46), and $t_{e}=0.7\;\text{mm}$ (r=0.15), with the monolithic mortar case serving as the benchmark. This parametric study quantifies the toughening mechanisms arising from soft layer incorporation in the architected multi‐material assembly.

**FIGURE 5:**
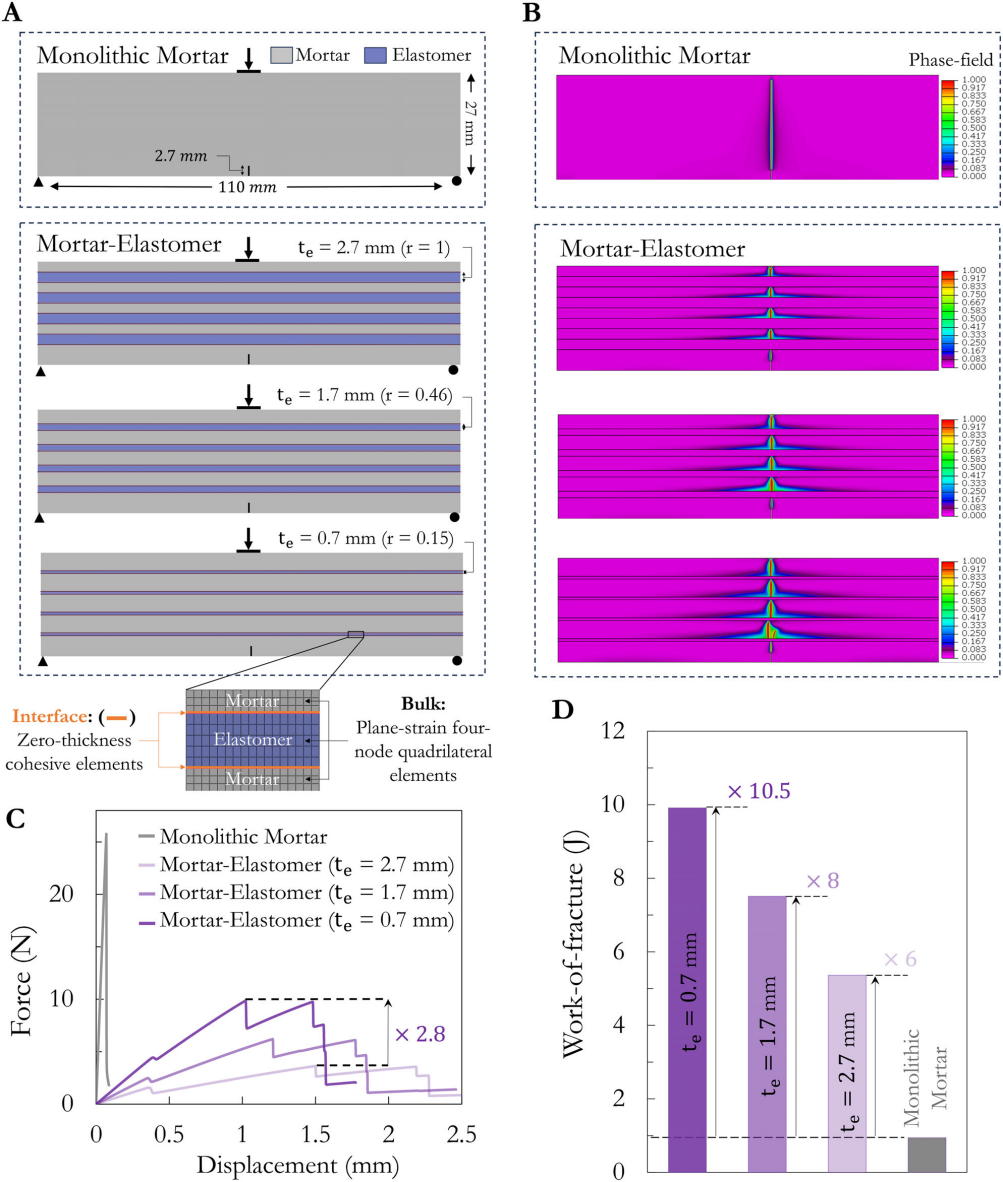
Effect of elastomer layer thickness on the fracture response of mortar‐elastomer multi‐materials with toughening mechanisms similar to those observed experimentally (arrest, bridging, re‐nucleation). (A) finite‐element model configuration showing single‐edge notch bend (SENB) specimen (110mm × 27mm) with alternating mortar‐elastomer layers. Three elastomer thicknesses were investigated: $t_{e}=0.7$ mm (r=0.15), $t_{e}=1.7$ mm (r=0.46), and $t_{e}=2.7$ mm (r=1.0), where r represents the elastomer‐to‐mortar thickness ratio. The finite‐element mesh consists of four‐node plane‐strain quadrilateral elements for bulk phases with zero‐thickness cohesive elements at interfaces. (B) Phase‐field damage profiles at complete fracture, highlighting distinct crack profiles: monolithic mortar exhibits single straight crack propagation, while all multi‐material configurations display discontinuous, multi‐stage crack paths exclusively through mortar layers with intact elastomer bridging. (C) Force‐displacement curves demonstrating transition from catastrophic brittle failure in monolithic cast mortar to graceful multi‐stage fracture in multi‐material systems. Decreasing elastomer thickness increases initial stiffness and peak force while preserving the three characteristic toughening mechanisms (arrest, bridging, re‐nucleation). (D) Work‐of‐fracture plot demonstrating substantial toughening with decreasing elastomer thickness, achieving approximately 6‐fold, 8‐fold, and 10.5‐fold enhancements over monolithic cast mortar for $t_{e}=2.7,1.7,\mathrm{and}\;0.7\mathrm{mm}$ configurations, respectively.

Figure 5 presents the results of the finite‐element simulations pertaining to the elastomer layer thickness effects on the overall fracture response. The computational domain consists of a 110mm×27mm SENB beam with alternating mortar‐elastomer layers (Figure 5A). Finite‐element discretization was performed using four‐node plane‐strain quadrilateral elements for the two bulk phases (i.e., hard mortar and soft elastomer domains), whereas for the interface between them, zero‐thickness cohesive elements were inserted at inter‐material boundaries (Figure 5A).

The crack propagation profiles at failure (Figure 5B) reveal distinct fracture modes between the monolithic and hard‐soft multi‐material configurations. The monolithic mortar exhibits a single, straight crack path along the beam centerline as anticipated. In contrast, all three multi‐material configurations display discontinuous crack propagation characterized by crack arrest, crack bridging by soft material, and crack re‐nucleation, for all three thickness ratios. Consistent with experimental observations (Figures 2E and 3A), cracks advance exclusively through mortar layers while elastomer layers remain intact (i.e., undamaged) throughout the process. Specifically, cracks initiate at the pre‐existing notch and propagate through the first mortar layer before being arrested at the mortar‐elastomer interface. Following substantial elastomer deformation, crack re‐nucleation occurs in the adjacent mortar layer, enabling the crack bridging mechanism observed experimentally (Figure 3A, i–vi). This arrest‐bridging‐re‐nucleation sequence repeats throughout the beam depth as a multi‐faceted toughening mechanism.

The force‐displacement curves (Figure 5C) quantify the contrast between brittle failure in monolithic mortar and graceful failure in multi‐material cases. While the monolithic case fails abruptly at minimal displacement, all multi‐material configurations exhibit multi‐stage failure characterized by repeat cycles of discrete load drops followed by hardening, resulting in the substantially enhanced ductility observed experimentally (Figure 3A). Each cycle corresponds to the sequence of hard layer fracture, arrest at the hard‐soft material interface, elastomer deformation, and crack re‐nucleation described above. As the elastomer‐to‐mortar thickness ratio decreases from r=1 ($t_{e}=2.7\;\mathrm{mm}$) to r=0.15 ($t_{e}=0.7\;\mathrm{mm}$), the initial stiffness, peak load, load drop magnitude, and post‐drop hardening stiffness all increase due to the larger mortar volume fraction. Indeed, the peak load, indicative of the load‐bearing capacity, increases by 2.8‐fold as the elastomer‐to‐mortar thickness ratio reduces from r=1 to r=0.15 ($t_{e}=2.7\;\mathrm{mm}$ to $t_{e}=0.7\;\mathrm{mm}$). Furthermore, the work‐of‐fracture (Figure 5D) also increases dramatically as the elastomer‐to‐mortar thickness ratio decreases. In fact, substantial toughening of approximately 6‐fold, 8‐fold, and 10.5‐fold was achieved for the multi‐material system compared to the monolithic cases for thicknesses of 2.7mm (r=1), 1.7mm (r=0.46), and 0.7mm (r=0.15), respectively. The increase in both load‐bearing capacity and work‐of‐fracture by reduction of the soft material indicates the existence of a desired layer thickness ratio. The reduction of the soft layer thickness efficiently balances two critical factors: the load transfer between layers and the cushioning from the soft material's large deformation, which is necessary to maintain the gradual multi‐stage fracture (and ductility) brought forth by the aforementioned crack arrest, bridging, and re‐nucleation mechanisms. Based on the simulation results, it can therefore be hypothesized that the enhancement of the two mechanical properties, i.e., load‐bearing capacity and fracture toughness, can be reconciled with ductility, using the utility offered by the soft material in multi‐material designs, in contrast to monolithic counterparts (Figure 5C).

Taken together, these simulations establish a clear geometric design trend for layered mortar–elastomer composites: decreasing the soft‐to‐hard thickness ratio from r=1 to r=0.15 systematically increases both peak load (by up to 2.8‐fold) and work‐of‐fracture (by up to 10.5‐fold) relative to monolithic mortar, while preserving the same arrest–bridging–re‐nucleation sequence of toughening mechanisms. This identifies thin, continuous soft layers as an efficient means to reconcile stiffness, load‐bearing capacity, and toughness in architected cementitious systems.

#### 2.2.2 Effect of Soft Material Properties

While the silicone elastomer employed in the experiments exhibited particularly low stiffness ($G_{e}=0.1\;\mathrm{MPa}$), it nonetheless contributed to significant fracture toughness and ductility enhancements in the architected multi‐material assemblies (Figure 2A,C,D). To explore the potential of alternative elastomer properties with higher stiffness (readily available commercially or synthesized with tailored properties) finite‐element simulations were conducted using elastomer moduli (i.e., rubber shear modulus) $G_{e}$ of 1, 5, and 10 MPa, representing 10‐, 50‐, and 100‐times the silicone modulus used experimentally ($G_{e}=0.1\;\mathrm{MPa}$). These values span the range achievable through modifications in crosslink density and filler content (e.g., carbon black, fumed silica) within elastomeric materials [83]. A constant Young's modulus was used for the mortar $E_{m}=8.8\;\text{GPa}$. The simulations employed the thin elastomer configuration from the previous section (Figure 5 with $t_{e}=0.7\;\mathrm{mm}$, i.e., elastomer‐to‐mortar thickness ratio r=0.15) to maximize the effects of modulus variation.

**FIGURE 6:**
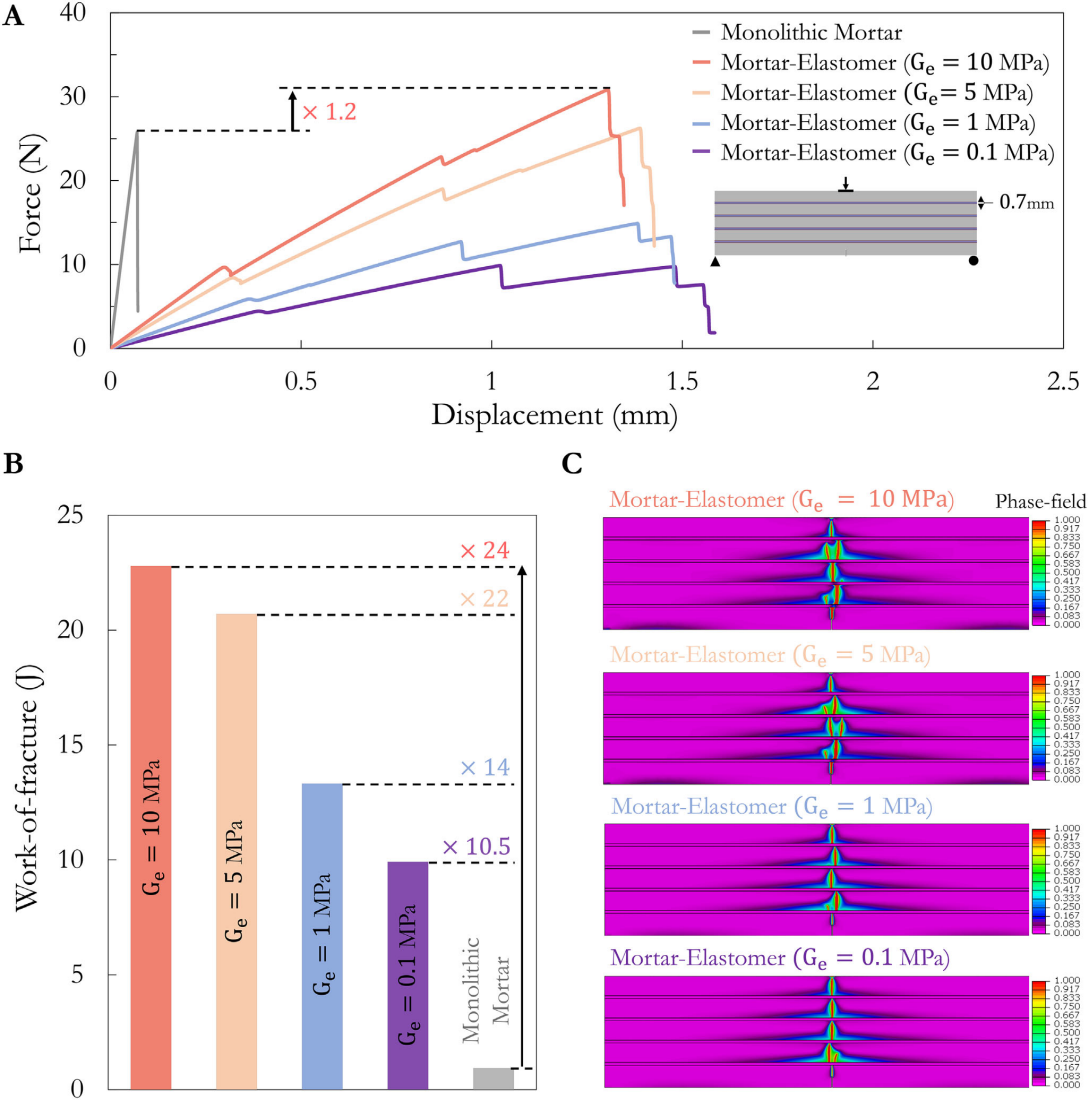
Effect of elastomer stiffness on fracture response of mortar‐elastomer multi‐materials with thin elastomer layers from the thickness study ($t_{e}=0.7\;\text{mm}$, i.e., elastomer‐to‐mortar thickness ratio r=0.15). (A) Force‐displacement curves for the mortar‐elastomer multi‐material assemblies with elastomer moduli (i.e., rubber shear moduli) ranging from 0.1 to 10 MPa compared to monolithic mortar. Increase in elastomer stiffness enhances load‐bearing capacity while maintaining the graceful multi‐stage fracture characteristics of multi‐materials. The 5 and 10 MPa configurations achieve a similar and 1.2‐fold increase in the peak force compared to monolithic mortar, respectively. (B) Work‐of‐fracture plot demonstrates dramatic toughening across all elastomer stiffness values, with 10.5‐fold, 14‐fold, 22‐fold, and 24‐fold improvements over monolithic mortar for 0.1, 1, 5, and 10 MPa configurations, respectively. (C) Phase‐field damage profiles at complete fracture, revealing distinct fracture patterns: discontinuous, multi‐stage crack paths exclusively through mortar layers arrested and bridged by intact elastomers for all stiffness cases. Compliant elastomer (0.1 MPa) produces relatively straight crack paths, while stiffer elastomers generate increasingly more tortuous damage patterns and a higher distributed damage (i.e., apparent crack branching and multiple re‐nucleation sites within mortar layers), indicating enhanced energy dissipation through increased fracture surface area.

Figure 6A demonstrates that elastomer stiffness provides a powerful design parameter for engineering the mechanical performance, particularly to overcome the mutual exclusivity between strength and fracture toughness (and ductility). While the baseline multi‐material case with $G_{e}=0.1\;\mathrm{MPa}$ achieved substantial fracture toughness improvement (10.5‐fold increase illustrated in Figure 5D), its load‐bearing capacity remained below the monolithic benchmark (∼8 N vs. ∼25 N). Increasing elastomer stiffness systematically enhances load‐bearing capacity: in the configurations with $G_{e}=10\;\text{MPa}$, the peak load increases by 1.2‐fold compared to the monolithic mortar case, respectively. This result highlights a remarkable finding: despite replacing 15% of the mortar thickness with an elastomer that has a modulus three orders of magnitude lower ($G_{e}=10\;\text{MPa}$ vs. $E_{m}=8.8\;\text{GPa}$), the multi‐material system achieves a 1.2‐fold higher load‐bearing capacity than the monolithic mortar. The work‐of‐fracture (Figure 6B) exhibits even more significant improvements with the 1, 5, and 10 MPa configurations, achieving 14‐fold, 22‐fold, and 24‐fold enhancements over monolithic mortar, respectively.

The phase‐field damage profiles (Figure 6C) elucidate the underlying fracture mechanisms involving a similar multi‐faceted arrest‐bridging‐re‐nucleation sequence, reported in Figure 5B. The case with the most compliant elastomer, i.e., $G_{e}=0.1\;\mathrm{MPa}$, produces relatively straight crack paths along the beam centerline. As the elastomer stiffness ($G_{e}$) increases, progressively more intricate crack propagation paths emerge as indicated by the apparent spread of damage in the hard material shown in Figure 6C (i.e., crack branching and multiple re‐nucleation sites within individual mortar layers). The emergence of damage spread in the hard material can be attributed to the increase in the ability to better transfer load between the hard layers with increasing elastomer stiffness (undergoing higher stresses). This increased load transfer engages larger volumes of mortar under tension (i.e., tension‐stiffening), creating multiple stress‐localization sites and promoting the spread of cracking in the hard material (ultimately seen as multiple crack re‐nucleation sites).

The computational results demonstrate that multi‐material assemblies enable a disruptive approach to the classical strength‐toughness trade‐off in cementitious composites. By integrating thin elastomeric layers with engineered stiffness into brittle cement‐based matrices, we computationally demonstrated simultaneous enhancements in both properties: load‐bearing capacity (strength) (by 1.2‐fold) and work‐of‐fracture (toughness) (by 24‐fold) as shown for the case of $G_{e}=10\;\mathrm{MPa}$ and $t_{e}=0.7\;\mathrm{mm}$. This design approach, using a thin soft layer with high stiffness, can open new frontiers for experimentally creating damage‐tolerant structural cement‐based materials that transcend the limitations of conventional monolithic systems.

These results provide a second, complementary design trend: at fixed thin‐layer geometry ($t_{e}=0.7\;\mathrm{mm}$, r=0.15), increasing soft‐layer stiffness from $G_{e}=0.1\;\mathrm{to}\;10\;\mathrm{MPa}$ transforms the layered composite from a compliant system with high toughness but reduced strength into an architected assembly that simultaneously exceeds monolithic mortar in both peak load (i.e. strenght) and fracture toughness. In practical terms, thin but relatively stiff elastomer layers are identified as a particularly promising regime for overcoming the classical strength–toughness trade‐off in cementitious materials.

### 2.3 Experimental Validation of Numerically Predicted Design Trends and Findings

To assess whether the computational design trends predicted by the phase‐field‐CZM framework are experimentally realizable, additional 3D‐printed (3DP) hard‐soft multi‐material architected designs were fabricated and tested for flexural strength under three‐point bending (3PB) and for fracture toughness under single‐edge notch bend (SENB) loading. Two investigations were performed with respect to the effect of soft material geometry (i.e., soft layer thickness) and soft material properties (i.e., soft material stiffness). Informed by the numerical findings, the two experimental investigations were carried out by (i) reducing the soft‐layer thickness while maintaining the same hard‐soft mortar‐silicone layered architecture used in the numerical section (Figure 5), and (ii) increasing the soft‐layer stiffness by replacing silicone with a stiffer elastomer (polyurethane) while keeping the thin ($t_{e}=0.7\;\mathrm{mm}$) soft‐layer thickness as was used in the numerical parametric study (Figure 6).

#### 2.3.1 Effect of Soft‐Layer Thickness (Silicone)

**FIGURE 7:**
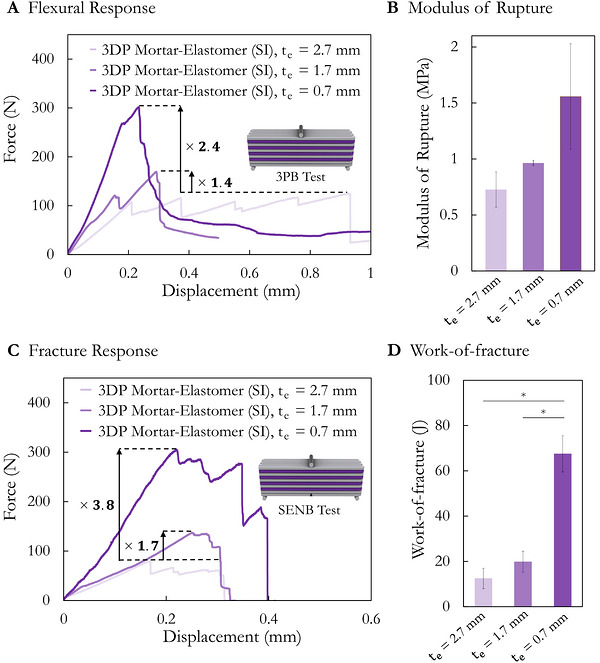
Experimental validation of soft‐layer thickness effects on the flexural and fracture responses of 3D‐printed (3DP) mortar‐silicone layered configurations. (A) Representative three‐point bending (3PB) force‐displacement responses for 3DP layered mortar‐silicone configurations with soft material thicknesses $t_{e}=2.7$, 1.7, and 0.7 mm, demonstrating an increase in both stiffness and peak load‐bearing capacity with reduced $t_{e}$ while preserving multi‐stage non‐catastrophic failure. (B) Modulus of rupture (MPa) for 3DP layered mortar‐silicone configurations with soft material thicknesses $t_{e}=2.7$, 1.7, and 0.7 mm. (C) Representative single‐edge notch bend (SENB) force‐displacement responses for 3DP layered mortar‐silicone configurations with soft material thicknesses $t_{e}=2.7$, 1.7, and 0.7 mm, demonstrating an increase in the area under the curves with reduced $t_{e}$ while preserving stable crack‐growth behavior and progressive load drops/hardening cycles across all thicknesses. (D) Work‐of‐fracture (Joules) for 3DP layered mortar‐silicone configurations with soft material thicknesses $t_{e}=2.7$, 1.7, and 0.7 mm, calculated as the area under the force‐displacement curves in (C), demonstrating statistically significant enhancement of toughness (*p<0.05) for the 0.7 mm case.

To verify the numerical predictions associated with decreased soft‐layer thickness, additional architected hard‐soft composites were 3D printed and tested, with the silicone layer thickness decreased from the baseline design ($t_{e}=2.7\;\mathrm{mm}$) to $t_{e}=1.7\;\mathrm{mm}$ and $t_{e}=0.7\;\mathrm{mm}$. Representative force‐displacement curves from 3PB and SENB tests are illustrated in Figure 7A,C. In both cases, decreasing soft material thickness produces a systematic increase in the initial stiffness and peak force while preserving the defining characteristic of the hard‐soft system: non‐catastrophic multi‐stage fracture characterized by discrete load drops corresponding to crack re‐nucleation from subsequent layers.

Quantitatively, reducing the silicone thickness from 2.7 to 1.7 mm and 0.7 mm in the 3PB test increases the initial stiffness 3.6‐fold and 6.4‐fold, and the peak load 1.4‐fold and 2.4‐fold, respectively. This enhancement confirms that reducing the soft‐layer thickness improves both resistance to deformation and load‐bearing capacity, thereby increasing the modulus of rupture (MOR) (Figure 7B), while preserving progressive failure. Similarly, the SENB test shows peak force increases by 1.7‐fold and 3.8‐fold for the same thickness reductions. The experimentally observed improvements in load‐bearing capacity align well with numerical predictions for soft‐layer thickness effects (Figure 5C), which similarly demonstrate pronounced increases in stiffness and peak force while maintaining the arrest‐bridging‐re‐nucleation fracture toughening mechanism.

In addition to the enhancement of initial stiffness and load‐bearing capacity, the energy required for fracture in the SENB test also increases, as evidenced by the larger area under the force‐displacement curves in Figure 7C, calculated as the work‐of‐fracture in Figure 7D. The increase in the work‐of‐fracture aligns with the numerical predictions in Figure 5D.

#### 2.3.2 Effect of Soft‐Layer Stiffness (Polyurethane vs. Silicone)

**FIGURE 8:**
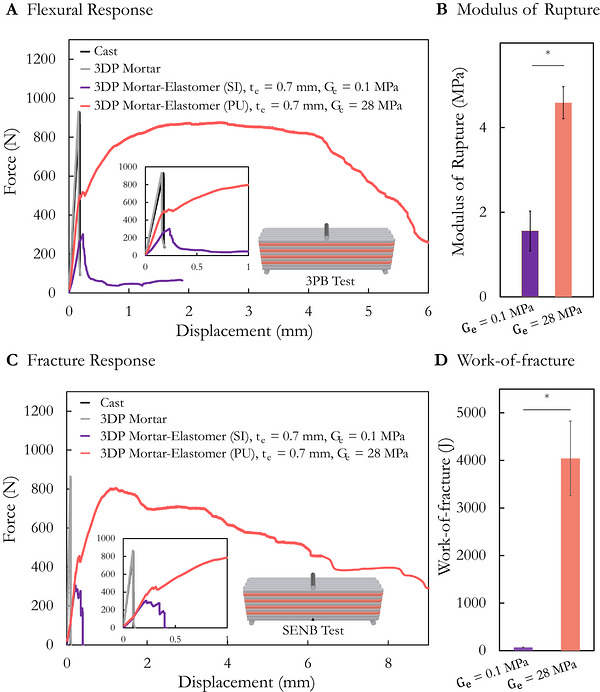
Experimental validation of soft‐layer stiffness effects on the flexural and fracture responses of 3D‐printed (3DP) mortar‐elastomer layered configurations. (A) Representative force‐displacement curves from 3PB of layered mortar‐elastomer specimens with fixed elastomer‐layer thickness ($t_{e}=0.7$ mm) compared with monolithic cast and 3DP mortar counterparts. Increasing the elastomer stiffness from $G_{e}=0.1$ MPa (Mortar‐SI, silicone) to $G_{e}=28$ MPa (Mortar–PU, polyurethane) substantially increases the initial flexural stiffness and peak load. (B) Modulus of rupture (MPa) for the 3DP layered Mortar‐PU configuration compared to its Mortar‐SI counterpart for a fixed elastomer‐layer thickness ($t_{e}=0.7$ mm), demonstrating statistically significant and substantial enhancement of modulus of rupture (*p<0.05). (C) Representative SENB force‐displacement curves for the same material set, showing that the stiffer soft layer (Mortar‐PU) sustains substantially higher post‐peak load and dissipates more energy (larger area under the curve) than the more compliant soft layer (Mortar‐SI), while maintaining load‐bearing capacity comparable to the monolithic cast and 3DP mortar counterparts. (D) Work‐of‐fracture (Joules) for the 3DP layered Mortar‐PU configuration compared to its Mortar‐SI counterpart for a fixed elastomer‐layer thickness ($t_{e}=0.7$ mm), calculated up as the area under the force‐displacement curves in (C), demonstrating statistically significant and substantial enhancement of toughness (*p<0.05).

To verify the numerical predictions associated with the increased soft‐layer stiffness, additional architected hard‐soft composites were 3D printed and tested using polyurethane (PU) as the soft material, an elastomer 280‐fold stiffer than silicone, with shear modulus $G_{e}=28$ MPa compared to silicone's $G_{e}=0.1$ MPa (see mechanical characterizations in Figure S7). These composites were fabricated using a thin soft layer thickness of $t_{e}=0.7$ mm, consistent with the numerical parametric study (Figure 6). Representative force‐displacement responses under 3PB and SENB tests are illustrated in Figure 8A,C, respectively. Two key experimentally‐informed insights emerge:

(i) Recovery of load‐bearing capacity while enhancing ductility: The Mortar‐PU (Polyurethane) composite achieves a peak force on par with monolithic cast and 3D‐printed (3DP) mortar references (Figure 8A), while exhibiting significantly higher ductility, with strain‐at‐peak 22.6‐fold higher than both monolithic counterparts, compared to a 10.9‐fold enhancement observed in Mortar‐SI (Silicone) composites of the same thickness. The modulus of rupture increased by 2.9‐fold for the Mortar‐PU composite compared to its Mortar‐SI counterpart (Figure 8B).
(ii) A significant increase in fracture toughness without sacrificing load‐bearing capacity: the Mortar‐PU layered composite exhibits an exceptionally larger area under the curve (i.e., work‐of‐fracture) (Figure 8C,D), reaching a staggering 124‐fold increase relative to the monolithic references, compared with a 1.82‐fold increase for Mortar‐SI at the same thickness (0.7 mm) relative to the monolithic cast and 3DP references. Both layered composite systems transition from the abrupt post‐peak loss observed in monolithic mortars to stable crack growth (Figure 8C), with Mortar‐PU sustaining remarkably higher post‐peak load and exhibiting extended hardening response. This behavior is indicative of a broader range of microstructural energy dissipation mechanisms in the hard layers (layer‐wise crack nucleation and micro‐cracking) and interfaces (interfacial debonding and damage).

DIC analysis (Figures S11 and S12) further elucidates the underlying fracture mechanisms in both Mortar‐SI and Mortar‐PU layered composites at the 0.7 mm soft layer thickness. For Mortar‐SI, the crack initiates at the notch tip and propagates through the first few mortar layer, followed by repeated crack arrest/deflection at mortar‐silicone interfaces and re‐nucleation in subsequent mortar layers (Figure S11). The discrete load drops and subsequent hardening segments correspond to successive mortar‐layer fracture events, while the silicone layers remain intact, providing bridging and load transfer between the layers.

In contrast, for the Mortar‐PU composite (Figure S12), after initiation at the notch tip and upward propagation through the mortar, the crack path becomes increasingly tortuous in interaction with the interface: pronounced deflection along mortar‐elastomer interfaces, sustained bridging between cracked mortar segments, and progressive interfacial debonding enable continued load transfer during the post‐mortar cracked regime (Figure S12, and Movies S3 and S4). The result is a long, stable post‐peak response with gradual load decay, consistent with a broader spectrum of energy‐dissipation processes across (i) the hard phase (layer‐wise crack nucleation), (ii) interfaces (debonding and damage) as evidenced by the shear and Von Mises strain evolution in Movies S3 and S4, and (iii) the soft phase (large‐strain deformation and bridging). These trends are in agreement with predictions from the coupled phase‐field‐CZM framework (Figure 6B).

**FIGURE 9:**
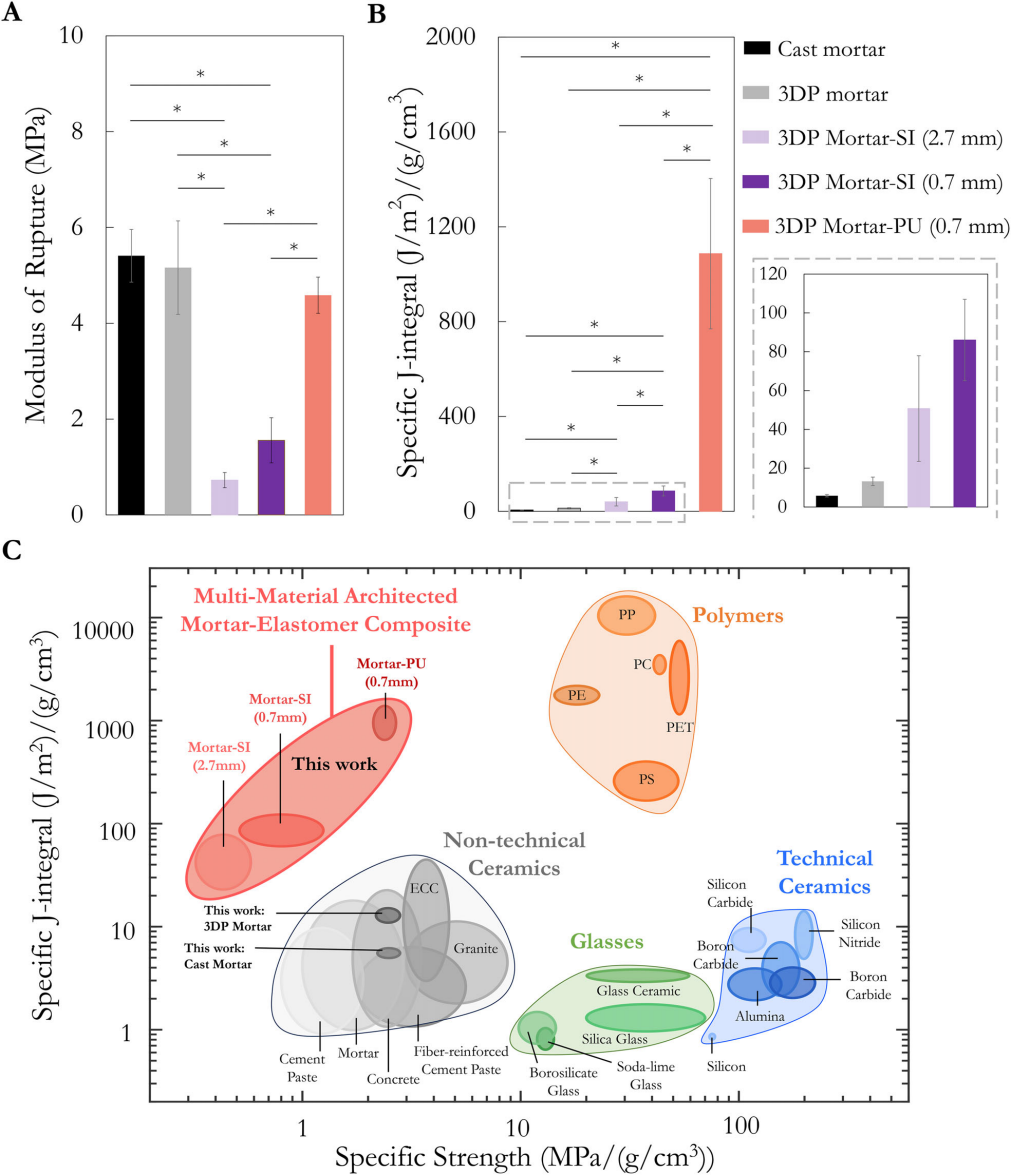
Experimental validation of fracture resistance and strength improvements. (A) Modulus of rupture (MOR) showing that thinning silicone in the SI‐based composites increases strength from 0.76 MPa (2.7 mm) to 1.6 MPa (0.7 mm), while the PU‐based composite (at 0.7 mm) achieves 4.6 MPa, which is statistically on par with cast mortar (5.4 MPa) and 3D‐printed (3DP) mortar (5.5 MPa), demonstrating simultaneous strength and toughness enhancement as predicted numerically. (B) Specific J‐integral for cast mortar, 3DP mortar, and 3DP layered multi‐materials. Decreasing silicone thickness increases fracture resistance from 51 J/$m^{2}$ /(g/${\mathrm{cm}}^{3}$) (at $t_{e}=2.7\;\mathrm{mm}$) to 86 J/$m^{2}$ /(g/${\mathrm{cm}}^{3}$) ($t_{e}=0.7\;\mathrm{mm}$). Replacing silicone with polyurethane at $t_{e}$ = 0.7 mm yields a pronounced increase to 1087 J/$m^{2}$ /(g/${\mathrm{cm}}^{3}$). (C) Ashby Plot of the specific fracture toughness (J‐integral) versus specific strength comparing the hard‐soft layered architected mortar‐elastomer composites designed in this work with monolithic cementitious references from this work and those in the literature inclduing cement paste [84, 85, 86, 87, 88, 89, 90], mortar [32, 84, 85, 87, 88, 91], concrete [85, 91, 92, 93, 94, 95, 96, 97], fiber‐reinforced cement paste [98, 99], and engineered cementitious composite (ECC) [100]. The sequence of Mortar‐SI (2.7 mm → 0.7 mm) and Mortar‐PU (0.7 mm) illustrates the systematic performance trajectory achieved by tuning soft‐layer thickness and stiffness.

The mechanical properties of the cases shown in Figure 8 are reported in Figure 9. The recovery of flexural strength to levels comparable to the cast and 3D‐printed (3DP) monolithic mortars is captured by the MOR in Figure 9A: the Mortar‐PU ($t_{e}=0.7$ mm) system reaches 4.6 MPa, compared to 5.4 MPa (cast mortar) and 5.5 MPa (3DP mortar), corresponding to 0.85× and 0.84× of the cast and 3DP counterparts, respectively. Statistical comparisons indicate that the MOR of the PU‐based composite is not significantly different from those of the reference monolithic cast and 3DP mortar. Furthermore, the fracture‐toughness enhancement is quantified using the density‐normalized specific J‐integral (Figure 9B). For each specimen, the reported specific J‐integral value corresponds to the point marked “Last Mortar Layer Cracking” on the representative R‐curves in Figure S13, i.e., the final fracture event in the uppermost mortar layer. The Mortar‐PU system ($t_{e}=0.7$ mm) reaches 1087 J/$m^{2}$/(g/${\mathrm{cm}}^{3}$), corresponding to a 12.7−fold increase relative to Mortar‐SI at the same thickness (86 J/$m^{2}$/(g/${\mathrm{cm}}^{3}$)), a 21.3−fold increase relative to Mortar‐SI with thicker elastomer layers ($t_{e}=2.7$ mm; 51 J/$m^{2}$/(g/${\mathrm{cm}}^{3}$)), an 82.2−fold increase relative to monolithic 3DP mortar (13.2 J/$m^{2}$/(g/${\mathrm{cm}}^{3}$)), and a 187−fold increase relative to monolithic cast mortar (5.80 J/$m^{2}$/(g/${\mathrm{cm}}^{3}$)). Together, these results demonstrate that decreasing soft‐layer thickness while increasing soft‐layer stiffness simultaneously amplifies load‐bearing capacity, stable post‐peak deformation, and fracture toughness through synergistic hard/soft/interfacial dissipation mechanisms.

Taken together, the experimental findings highlight the opportunity for substantial improvements in the engineering properties of cement‐based composites enabled by multi‐material additive manufacturing, using relatively small amounts of appropriately selected soft material classes with appropriate stiffness. The experiments further validate the numerical prediction that a thin, stiff soft material integrated between hard mortar can achieve simultaneous enhancement of strength and fracture toughness, circumventing the classical strength‐toughness trade‐off. More broadly, the combined approach demonstrates how constitutive computational probes can rapidly identify superior regions of the design space, which can then be experimentally validated and realized, and, in turn, used to further probe the design space computationally.

To contextualize the mechanical performance of the proposed architected hard‐soft cementitious composites relative to conventional cement‐based systems and broader material classes, Figure 9C plots the specific J‐integral from SENB tests against strength (MOR from 3PB) on an Ashby plot. The monolithic cementitious cases tested in this work as references (cast mortar and 3DP mortar) fall within (conservatively, at the upper bound of) the fracture toughness and flexural strength properties characterized from literature for mortar [32, 84, 85, 87, 88, 91], and concrete [91, 92]. The properties of cement paste [84, 85, 86, 87, 88, 89, 90], fiber‐reinforced cement paste [98, 99], concrete [85, 91, 92, 93, 94, 95, 96, 97], and engineered cementitious composite (ECC) [100] are also demonstrated for comparison, within the region characteristic of non‐technical ceramics. All cementitious properties are reported for laboratory specimens at 7 days of age to enable consistent, same‐age comparison with the monolithic and composite systems investigated in this work.

In contrast to cast and 3DP cementitious counterparts, the layered architected hard‐soft systems occupy a distinct, large region with substantially higher fracture toughness (J‐integral), shown in red. The baseline Mortar‐SI configuration with $t_{e}=2.7\;\mathrm{mm}$ lies well above monolithic mortars in terms of fracture toughness, but at a comparatively lower strength than the 3DP and cast mortar counterparts. Thinning the silicone layer to $t_{e}=0.7\;\mathrm{mm}$ shifts the composite further toward the upper right side of the plot, improving the strength. More substantially, an even larger shift toward the upper right side of the plot is achieved by increasing the soft‐layer stiffness at the same thin‐layer geometry (Mortar‐PU, $t_{e}=0.7\;\mathrm{mm}$), where the cementitious composite properties move into a regime of statistically similar strength to monolithic cases (maintaining mortar‐like strength) while achieving polymer‐like fracture resistance, effectively placing the layered cementitious composite outside the typical non‐technical ceramics envelope and into a higher‐performance domain of the Ashby plot. These results demonstrate that multi‐material architected cementitious composites provide a strategic new design pathway for engineering fracture toughness and strength, rather than relying on conventional microstructural modifications (e.g., fiber bridging or strain‐hardening cementitious matrices).

In combination, Figure 7, 8, 9 thus translate the numerical parametric trends into experimentally verified design guidelines: (i) thinning the soft layer (at fixed elastomer) moves the composite upward and modestly rightward on the Ashby map (higher toughness with increased strength), and (ii) replacing silicone with a stiffer polyurethane at the same thin‐layer geometry produces a substantial rightward/upward jump into a domain of polymer‐like fracture resistance at mortar‐like strength. This trajectory provides a transferable blueprint for designing other layered mortar‐elastomer systems.

#### 2.3.3 Fracture Analysis: Acoustic Emission (AE) Detection of Damage Evolution

**FIGURE 10:**
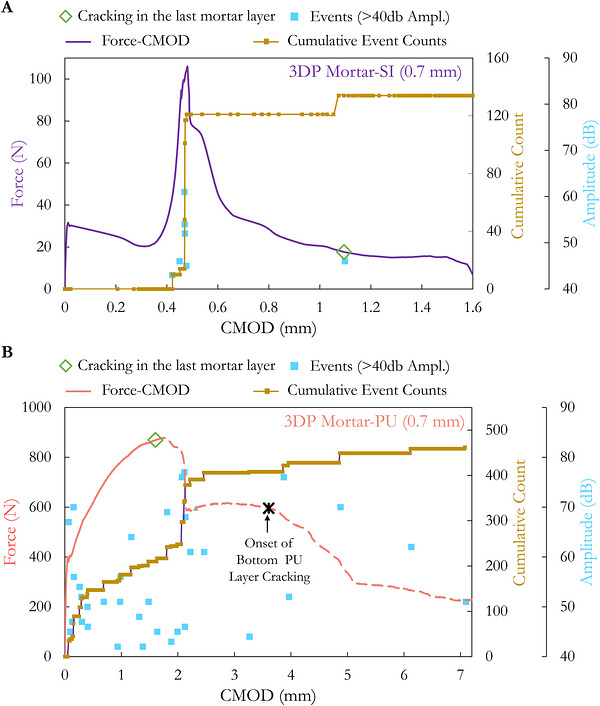
Acoustic Emission (AE) event evolution during SENB tests for Mortar‐SI and Mortar‐PU layered architectures. Force‐CMOD response overlaid with cumulative AE event count and event amplitude (dB) versus CMOD for A. Mortar‐SI and B. Mortar‐PU. The dashed force segment in (B) denotes the response after the last mortar layer cracking event, where load transfer is governed primarily by elastomer‐dominated bridging and interlayer friction while AE event activity continues to evolve.

To further elucidate the toughening and microstructural damage mechanisms underlying the distinct fracture responses of Mortar‐SI and Mortar‐PU layered composites, acoustic emission (AE) monitoring was conducted during the SENB tests. Specimens were instrumented with two broadband AE sensors and recorded using the Micro‐SHM acquisition system (see Experimental Section). Figure 10 reports the mechanical response (force‐CMOD) together with the cumulative AE event count and amplitude (dB) of individual events versus CMOD for both Mortar‐SI and Mortar‐PU systems. To further resolve the temporal localization and intensity of AE activity beyond the CMOD‐based representation, event‐level descriptors (counts and energy) are shown versus time in Figures S14 and S15 for Mortar‐SI and Mortar‐PU, respectively. Complementary cumulative representations of event count and cumulative AE energy over time, together with event amplitudes, are provided in Figures S16 and S17 for Mortar‐SI and Mortar‐PU, respectively.

For the case of Mortar‐SI layered composite, Figure 10A illustrates that the cumulative event count remains negligible over a relatively small CMOD range (0‐0.4 mm), then rises sharply within a very narrow CMOD window (0.4‐0.5 mm), just before the peak load. This increase is driven by a small number of relatively high‐amplitude events associated with the fracture of the mortar layers, followed by a single final event associated with the last mortar layer fracture, consistent with the burst‐like clustering seen in the time‐domain event descriptors (see Figures S14 and S16). The burst‐like events concentrated in the narrow CMOD range around the peak load are consistent with the nucleation and coalescence of localized tensile damage along the dominant tensile crack path observed in the mortar layers experimentally (see Figures S11, S14, S16, and S18). Beyond this point, the cumulative count rapidly approaches a plateau, and subsequent AE activity becomes sparse, indicating that further deformation is accommodated primarily by stable stretching and bridging of the silicone layers with limited additional damage.

In contrast, the case of Mortar‐PU composite exhibits a richer and more sustained AE signature. Figure 10B illustrates that the cumulative event count increases progressively throughout the test, including well into the post‐peak regime, consistent with the sustained time‐domain AE activity and cumulative energy accumulation (see Figures S15 and S17). AE activity intensifies around the first major load drop at a small CMOD range (<0.2 mm) and continues to accumulate as the load rises toward the peak over a very broad CMOD range (0.2‐2 mm), with both low‐ and high‐amplitude events distributed over such a large range. The dashed segment of the force‐CMOD curve marks the regime after the peak load corresponding to the last mortar‐layer cracking event has taken place; a regime where the structure continues to carry load while AE events persist, indicating ongoing damage and dissipation past the exhausted mortar cracking alone, all the way to a regime where the PU layer began to fail. This prolonged accumulation of AE activity into a post‐mortar‐fracture regime reflects the ability of the Mortar‐PU composite to sustain extended stable deformation and crack growth, enabled by the much stiffer PU interlayers and, consistently, by their higher measured interfacial strength and fracture energy relative to the Mortar‐SI interfaces (Figure S9).

The more sustained AE activity in the Mortar‐PU system, especially into the post‐peak regime, is consistent with the presence of additional dissipative mechanisms beyond mortar cracking, in line with the enhanced load‐bearing capacity and fracture toughness quantified in Figures 8 and 9.

It is also instructive to contrast the force‐CMOD responses of the two layered composites in Figure 10A,B. The Mortar‐SI system exhibits a noticeably higher compliance: following the first load drop (associated with the first mortar‐layer cracking event), the curve shows a pronounced softening over a finite CMOD interval on the order of 0.4 mm before re‐hardening occurs. This behavior is consistent with a scenario in which the very soft silicone interlayers undergo large elastic deformation and temporarily store strain energy, thereby delaying re‐engagement and load transfer to the remaining mortar layers. By contrast, the Mortar‐PU system displays a more immediate and sustained re‐hardening after the first load drop, with the force‐CMOD curve rising monotonically over a broader CMOD range. This response reflects the much higher stiffness of the PU interlayers compared to silicone, which promotes more efficient load redistribution back into the mortar layers and alters the post‐cracking composite response, even though the mortar‐elastomer stiffness contrast remains large in both systems.

**FIGURE 11:**
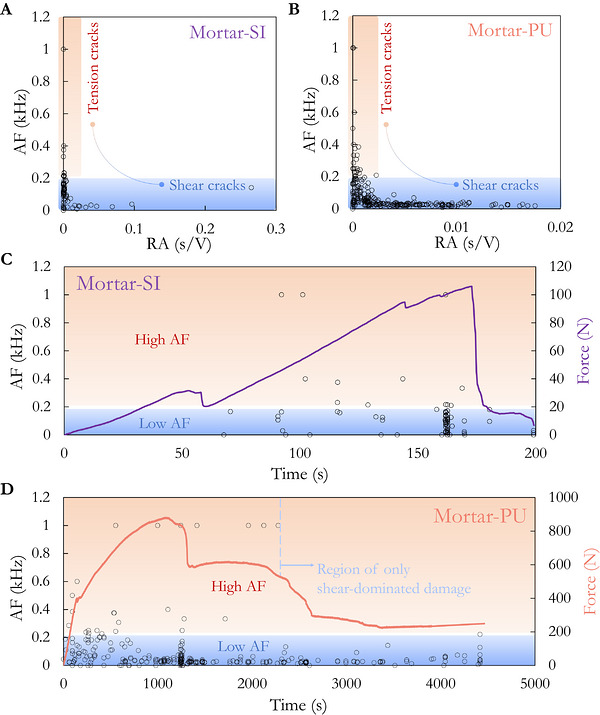
Acoustic Emission (AE) event characterization using average frequency (AF) versus risetime amplitude (RA) (in seconds over Volts, s/V) plot and fracture characteristics highlighted by the AF over time and force history plots. (A,B) AF‐RA distributions for Mortar‐SI and Mortar‐PU, respectively; (C,D) AF and force histories over time for Mortar‐SI and Mortar‐PU, respectively, highlighting correlations between AE characteristics and stages of damage.

Figure 11 provides more rigorous complementary AE descriptors in terms of the average frequency (AF) and risetime‐amplitude ratio (RA), both in AF‐RA space and as AF‐time space, overlaid with the force history. AF and RA are commonly used as qualitative indicators of dominant damage kinematics in brittle and quasi‐brittle materials: relatively fast, high‐frequency events with low RA are typically associated with tension‐dominated cracking, whereas slower events with lower AF and larger RA are often linked to shear‐dominated (e.g., interface‐controlled processes) such as frictional sliding, debonding, or crack‐face contact [101, 102, 103, 104].

For the Mortar‐SI layered composite, the AF‐RA plot in Figure 11A is dominated by a compact cluster of events at low RA and relatively high AF. This narrow distribution is consistent with a response governed primarily by discrete, rapid tensile‐like cracking events in the brittle mortar layers, with only limited evidence of slower, shear‐like damage processes. In other words, most of the AE energy in Mortar‐SI is released during short‐lived tensile microcracking episodes concentrated around the main fracture events.

In contrast, the Mortar‐PU composite exhibits a broader AF‐RA distribution (Figure 11B). While a low‐RA/high‐AF cluster is still present in this case, similar to Mortar‐SI, indicating tensile‐like cracking in the mortar, the event population additionally includes a substantial number of signals at higher RA and lower AF compared to Mortar‐SI. These additional events are consistent with enhanced contributions from slower shear‐dominated damage mechanisms such as interface debonding and continuous frictional sliding along crack faces (see Movies S3 and S4). The Mortar‐PU case, therefore, activates a richer set of dissipative pathways compared to Mortar‐SI, spanning both tensile and shear (e.g., interfacial) kinematics.

The AF‐time evolution for Mortar‐SI, overlaid with the force history in Figure 11C, further elucidates this behavior. Higher‐AF events concentrate during the initial post‐peak hardening that involves major mortar‐layer cracking episodes, in agreement with the burst‐like AE accumulation near peak load seen in Figure 10A. After the final mortar‐layer fracture, AE activity drops significantly to sporadic, relatively low‐intensity events, mirroring the plateau in cumulative event count in Figure 10A. This temporal pattern paints a picture of Mortar‐SI as a system in which damage accumulation is possible through a short phase of tensile cracking, followed by an acoustically quiet regime after all mortar layers are fractured, where deformation is carried mainly by stable silicone bridging and limited additional damage mechanisms.

For the case of Mortar‐PU, the AF‐time evolution in Figure 11D reveals a qualitatively different acoustic signature. During the early loading stage involving the initial mortar‐layer cracking, several high‐AF events take place, similar to Mortar‐SI, reflecting tensile cracking in the mortar, albeit with a significantly higher population of low‐AF events compared to Mortar‐SI. Nevertheless, after the last mortar‐layer cracking event (marked by a green diamond in Figure 10B), Mortar‐PU force‐CMOD response transitions into a low‐AF activity corresponding to a shear‐dominated damage mechanism. The energy dissipation at this stage is governed predominantly by elastomer and interfacial processes such as sustained PU bridging, local damage or yielding within the PU layers due to shear, and frictional/shear interactions at the interface or along previously formed crack paths (see Movies S3 and S4), rather than additional brittle mortar fracture. The annotated onset of the first PU‐layer cracking further confirms that the soft phase itself participates in energy dissipation while the composite still carries substantial load, underscoring the active role of the PU in stabilizing and extending the fracture process.

Finally, the spatial distribution of AE source locations along the specimen length is summarized in Figures S18 and S19, for Mortar‐SI and Mortar‐PU, respectively, providing a spatial counterpart to the temporal (Figure 10; Figures S14–S17) and kinematic (Figure 11) AE descriptors.

The AE findings corroborate the mechanical and fracture mechanics results, demonstrating that the stiffer (PU) interlayers both stabilize multi‐crack growth and activate additional dissipative shear‐dominated mechanisms, promoting a rare concurrent increase in load‐bearing capacity and toughness that mitigates the classical strength‐toughness trade‐off.

## 3 Conclusions

In this work, we propose a multi‐material additive manufacturing (MMAM) approach to engineer tough, ductile, and strong cement‐based architected composites enabled by an advanced automated multi‐material extrusion platform. The proposed technique allows engineering cementitious‐elastomeric multi‐material composites, defined here for the first time as architected cementitious composites (ACC), with the ability to control the geometry and material properties in an alternating co‐continuous fashion. Inspired by the siliceous‐proteinaceous layered architecture of the glass sponge *Euplectella aspergillum*, the design approach directly addresses the inherent brittleness, low fracture toughness, and limited ductility of monolithic cast and 3D‐printed (3DP) cementitious materials.

Initial implementation was performed with silicone (SI) layers having equal thickness to the mortar layers (2.7 mm). The proposed mortar‐silicone composites achieved up to 3.8‐fold and 8.7‐fold enhancements in fracture toughness and 11.7‐fold and 12.4‐fold enhancements in ductility relative to 3DP and cast mortars, respectively, while exhibiting rising R‐curves and graceful post‐peak behavior.

Experimental findings were then explored numerically using a coupled large‐deformation phase‐field‐cohesive zone (PF‐CZM) framework to probe a broader design space in terms of soft‐layer geometry (thickness) and soft‐layer stiffness (shear modulus). The simulations revealed that combining reduced soft‐layer thickness with higher soft‐layer stiffness can deliver up to a 24‐fold enhancement in numerically predicted work‐of‐fracture, while recovering the load‐bearing capacity to the levels of monolithic counterparts.

Building on the prediction of the numerically conceived designs, fabrication of layered mortar‐polyurethane (PU) composites with thin, stiff PU interlayers was subsequently carried out. These experimentally validated designs recovered flexural strength to levels statistically comparable to both cast and 3DP mortars, while achieving up to over two orders of magnitude higher fracture toughness and over an order of magnitude higher ductility. The specific J‐integral increased by approximately 82‐fold and 187‐fold relative to 3DP and cast mortars, respectively, and the ductility (strain‐at‐peak) increased by about 22.6‐fold relative to both monolithic references.

Across these layered architectures, three synergistic toughening mechanisms were identified and visualized experimentally (via load‐CMOD, R‐curves, DIC, and AE), underpinning the large performance enhancements: (i) crack arrest and deflection along the soft interlayers, generating tortuous crack paths and increased fracture surface area; (ii) crack bridging by the soft layers, which sustains load transfer during crack propagation (penetration or deflection); and (iii) discontinuous crack re‐nucleation in the hard layers following large deformation of the soft material, promoting distributed damage and graceful failure instead of catastrophic fracture.

The proposed MMAM technique and the layered mortar‐elastomer composites establish a new class of cement‐based architected multi‐materials that transcend the conventional brittleness of concrete. The proposed fabrication freedom instigates a design freedom that can unleash entirely new properties, pathways, and perspectives for next‐generation concrete structures. The proposed integrated manufacturing‐design‐engineering approach opens a scalable pathway for reimagining future concrete materials and structures where multi‐material concrete additive manufacturing is extended upon single‐material extrusion, where geometry and constituent selection can be design variables for engineering target combinations of strength, fracture toughness, and ductility.

While recent advances in additive manufacturing have enabled the architectural design of cement‐based materials with improved fracture toughness using a single constituent, current technologies remain limited in the achievable degree of enhancement, primarily due to constraints in introducing a second material class (e.g., a soft elastomer) and in exploiting higher degrees of freedom in architectural design (e.g., layered motifs) and geometry (e.g., layer thickness). While the implementation of the process toward higher technology readiness and at a larger construction scale requires further engineering such as the need for additional extrusion hardware (pump, extrusion, nozzle) and software (end effector manipulation and sequencing), these challenges are merely engineering problems that are within the realm of growing concrete additive manufacturing platforms at scale. Once the utility and impact of the proposed approach are demonstrated, as exemplified in this study, the process can evolve and impact a broad range of products and components across scales.

More specifically, by proposing a novel multi‐material extrusion process, this work charts a new pathway for engineering advanced composites with applications in civil infrastructure; buildings (e.g., 3DP concrete housing); marine energy infrastructure (e.g., onshore and offshore structures for solar, wind, and wave renewable energy); and aerospace hard‐soft assemblies for extreme loading conditions or loading combinations where load‐bearing under large (cyclic) deformation and damage tolerance is a virtue of engineering and design (e.g., wind, earthquake, wave, or conditions subjected to dynamic or impact loads). This work only scratches the surface in terms of design; the proposed multi‐material composites can be further optimized and tailored to achieve targeted mechanical responses and properties (e.g., fracture resistance, ductility, and load‐bearing capacity) per application‐specific requirements using the MMAM technique.

The proposed MMAM process is scalable, especially for infrastructure and housing, leveraging existing concrete additive manufacturing platforms that have grown to tens of meters in scale over the past several years [105] and extending them from single‐material to multi‐material fabrication. This approach can transform the growing use of form‐free, additively manufactured concrete structures by adding degrees of freedom through both (i) architecting mesoscale to macroscale geometry and (ii) selecting appropriate secondary constituents from other material classes to integrate with concrete, including opportunities for reinforcement. Moreover, the proposed experimental‐numerical workflow demonstrates a significant potential for the discovery of new designs of cementitious composites.

Beyond mechanical performance, the proposed techniques can also enable new degrees of freedom in tailoring functional characteristics (e.g., carbonation [106], thermoregulation [107]) by expanding existing single‐material additive manufacturing methods to multi‐material systems. In short, this work broadens the design space and unlocks a vast domain of advanced infrastructure composites with a viable path to scale.

## 4 Experimental Section

### 4.1 Multi‐Material Additive Fabrication of Architected Cementitious Composites (ACC)

The fabrication of both monolithic 3D‐printed (3DP) mortar and mortar‐silicone multi‐material samples was performed using a custom‐designed extrusion process based on a modified E3D ToolChanger Motion System (Figure S1) [108]. For both ACC sample types, the mortar syringe was secured into the toolhead (designated as T2, Figure S2) through a friction fit with U‐shaped slots. For multi‐material printing, the prepared silicone tube (Figure S3C) was loaded into the respective toolhead (designated as T3, Figure S2) and secured using a threaded rod with a 3DP insert. The printing sequence was initiated by placing an acrylic plate onto the print bed (Figure S2H).

G‐code files were then uploaded to Duet Web Control. As part of the G‐code, all axes, including the tool locking axis, were automatically zeroed. The G‐code was designed with purge segments for both mortar and silicone to stabilize pressure within each reservoir and ensure consistent extrusion, minimizing flow anomalies. For monolithic prints, the purge segment was implemented only on the first layer, while for multi‐material fabrications, a purge sequence was executed after each tool change to account for pressure irregularities in the inactive reservoirs. Upon completion, excess material from the purge segments was carefully removed, and the printed sample was placed into a curing chamber, maintained at 97 ± 2% relative humidity using the saturated solution of potassium sulphate [109], where it was cured for 7 days before testing.

### 4.2 Multi‐Material 3D‐Printer Design and Control

The custom multi‐material 3D‐printing system was built on an E3D ToolChanger platform, which provided a motion control system and a kinematic coupling mechanism for automated toolhead selection and parking (Figures S1 and S2A). Up to four independently configured toolheads were mounted (T0, T1, T2, & T3; Figure S2) and interchanged to broadly accommodate different printing techniques, including syringe‐based direct ink writing (DIW) and fused filament fabrication (FFF). The printer was controlled by a Duet 2 mainboard with a DUEX 5 expansion board, which supports multiple toolheads and associated sensors. Firmware control was implemented using RepRap Firmware (RRF) 3.0, enabling real‐time macro‐based tool definitions, toolpath execution, sensor integration, and parameter tuning through advanced G‐ and M‐code commands (Supporting Information).

Tool‐changing and priming operations were managed through G‐code macro calls handled by the onboard firmware. These macros define the move sequences and xyz‐locations required for homing, tool pickup, parking, and priming. All tuning parameters, including tool locations, accelerations, material‐specific priming procedures, and tool offsets, were configured locally on the machine and executed by the Duet board running RRF. This approach decoupled low‐level motion control from the toolpath file, allowing the print instructions to consist of high‐level commands for material deposition, while tool changes and transitions were managed on‐device. The hardware and firmware were engineered to accommodate the rheological and processing requirements of each material, manage time‐dependent behavior during standby, and ensure synchronized multi‐material assembly with minimal user intervention.

### 4.3 Design of Automated Multi‐Material Printing Logic for Toolpath Generation Using Grasshopper

Toolpaths for multi‐material printing were generated using an in‐house Grasshopper script within the Rhino environment. A beam geometry was first defined based on the desired sample size and given the volumetric constraint of cementitious and silicone syringes, as well as the mortar and silicone filament dimensions that make up the total volume. The final geometry measures 110 mm in length, 27 mm in height, and 28 mm in width. In the multi‐material printing sequence, the first two bottom layers were designed using cementitious mortar. The additional cementitious bottom layer was specifically included to allow for notching of the sample for the SENB test. Following these two layers, alternating layers of silicone and mortar were designed throughout the remainder of the geometry (four silicone and four additional mortar layers). The layer paths were arrayed with appropriate spacing to accommodate tool changes and were then combined into a single geometry (See Figure S4).

Grasshopper supports toolpaths that coordinate multiple materials within a single part, unlike traditional slicers, which were typically limited to single‐material deposition. Grasshopper was used to facilitate parametric generation of multi‐material toolpaths by integrating geometric modeling with material‐specific printing logic. Extrusion paths were computed based on input parameters such as specimen length, layer height, filament width, number of filaments, extrusion multiplier, and vertical printing speed, separately for mortar (Figure S4A) and silicone (Figure S4B). After generating the base layer geometry (Figure S4C), discrete toolpaths for each material were created (Figure S4D,E) and merged into a single geometry (Figure S4F). These paths were then deconstructed into Cartesian coordinates (Figure S4G,H), which were woven (or interlaced) in alternating layers (Figure S4I) to produce the final G‐code (Figure S4J).

Building on the parametric generation and weaving of material‐specific paths, this approach allowed seamless integration of geometry and printing logic for each material. In contrast to custom G‐code macros that require manual motion programming, the developed Grasshopper script automates tool sequencing and deposition routines, offering enhanced flexibility and efficiency in multi‐material printing.

### 4.4 Materials Formulation

Cementitious mortar was prepared using Heidelberg Type I/II cement (sieved through a 150 μm mesh to eliminate coarse particles and prevent clogging), ASTM C778 [110] standard sand, high‐range water‐reducing admixture (HRWRA, MasterGlenium 7700), and a viscosity‐modifying admixture (VMA, MasterMatrix 362). The mix design included a water‐to‐cement ratio (w/c) of 0.30 and a sand‐to‐cement ratio (s/c) of 0.5. For each batch, 250 g of cement, 125 g of sand, 71.2 g of deionized water, 1.5 g of HRWRA (0.2% by weight of cement), and 3.0 g of VMA (0.05% by weight of cement) were used (Figure S3A). Mixing was performed using a Twister Evolution Venturi vacuum mixer (Renfert), following a two‐step mixing procedure [29, 30, 111]. The first step involved 25 s of pre‐mixing, followed by 90 s of mixing at 400 rpm under 70% vacuum. After scraping the mortar from the container walls, the second step involves an additional 90 s of mixing at 400 rpm under 100% vacuum (schematically shown in Figure S3A).

### 4.5 Materials Preparation

The freshly prepared cementitious mortar was then loaded into a 150 mL syringe (schematically shown in Figure S3B). The syringe had a tapered tip with a 4 mm opening. The plunger was fitted with a rubber seal, into which four grooves (Figure S3B) were manually introduced to facilitate the escape of air in the syringe during compression. To minimize the entrapment of air bubbles, the mortar was loaded into the syringe in approximately 30 mL increments. Following each addition, the syringe was tapped until no visible bubbles remained. This procedure was repeated until the mortar level reached within 1 cm of the syringe's top. The inner walls of the syringe were then cleaned, and the plunger was lubricated with DuPont dry‐film lubricant before insertion. Finally, the plunger was manually depressed to prime the syringe until it reached the 150 mL mark. For the multi‐material sample fabrication, silicone was prepared using a DAP clear silicone (schematically shown in Figure S3C), compliant with ASTM C920 [112]. The tube tip was cut to match the 4 mm syringe opening.

### 4.6 Mechanical Properties Characterization

Fracture toughness was determined using the single‐edge notch bend (SENB) test according to ASTM E1820 [113]. Unnotched prismatic specimens with dimensions of 27 mm in depth, 28 mm in width, and 110 mm in length were prepared for this testing. In each SENB specimen, a 1.7mm deep notch was first introduced using a 2mm thick diamond saw, followed by an additional 1mm depth extension using a 0.3 mm thick razor blade to achieve the required sharp crack tip. All specimens were tested with a span‐to‐depth ratio of 3. Load versus CMOD curves were obtained experimentally from SENB tests. Crack extension was determined from the compliance of these curves, and the J‐integral was calculated as the sum of elastic and plastic components using the load, crack extension, and the plastic area under the load‐CMOD curve. Details of the calculations of the J‐integral and crack extension were provided in the Supporting Information. Work‐of‐fracture was evaluated as the area under the load‐displacement curve.

Unnotched three‐point bending tests using specimens of identical dimensions as SENB (27 mm × 28 mm × 110 mm) were conducted to determine the Strain‐at‐first‐crack and the ultimate strain. All specimens were tested with the span‐to‐depth ratio of three at a loading rate of $0.05\;mm\;min^{-1}$. Mechanical testing was performed using an electromechanical testing frame (MTS Criterion C45.305) equipped with a 20kN load cell. At least five replicate specimens of each type were tested, and the average values were reported.

The mechanical properties of the bulk hard mortar were obtained as follows: The Young's modulus was obtained using the compliance of the load versus CMOD plot, whereas the fracture toughness was determined as the initiation point in the J‐integral versus crack extension plot (Supporting Information).

The mechanical properties of the bulk soft elastomer (silicone and polyurethane) were obtained as follows: The rubber modulus was obtained as the initial slope of the stress–strain curve corresponding to a uniaxial tensile test performed on an uncut dogbone sample; whereas for the fracture toughness, the property was determined using the tear test (Figure S7).

The mechanical properties of the mortar‐elastomer (Silicone and Polyurethane) interfaces were characterized using a combination of flexural, shear, and interfacial fracture tests. Interface strength was evaluated under tension and shear using three‐point bending and unnotched antisymmetric four‐point bending tests, while interfacial fracture toughness was measured under Mode I (tension) and Mode II (shear) loading using SENB and notched antisymmetric four‐point bending tests, respectively (Figure S8). Further details of experiments are discussed in the Supporting Information. Representative load–displacement responses for all interface tests are provided in Figure S9.

### 4.7 Acoustic Emission Damage Detection and Fracture Analysis

Acoustic emission (AE) monitoring was employed to characterize damage evolution during SENB and 3PB tests of mortar‐silicone and mortar‐polyurethane specimens. Two wideband AE sensors (PKWDI, MISTRAS Group Inc.), operating in the frequency range of 200–1000 kHz and equipped with an integrated differential preamplifier, were installed on each specimen.

The sensors were symmetrically mounted on the side surface of the beam with a sensor‐to‐sensor spacing of 58 mm. The mid‐span cross‐section of the beam was centered between the two sensors to ensure that acoustic emissions associated with damage initiation and propagation in the mid‐span region were captured well, although damage outside this region was also captured. Prior to installation, the specimen surfaces were cleaned, and the sensors were tightly attached to the beam specimens using high‐vacuum grease (Molykote high vacuum grease). The sensors were further secured using tape to maintain stable contact throughout the tests.

Pencil lead break (PLB) tests were conducted to verify amplitude consistency and wave propagation velocity. Three PLB events were performed near each sensor, and the mean peak amplitudes were compared, with differences maintained within 6 dB. Signal arrival times from PLB events were used to estimate the wave propagation velocity in the specimen. For this purpose, a PLB test was performed along the straight line defined by the two collinearly mounted sensors, at a known distance from one sensor. The arrival times at the sensor closest to the PLB location and at the farthest sensor were measured. The wave propagation velocity was then calculated using the known sensor‐to‐sensor distance (58 mm) and the corresponding time difference between the first signal arrivals at the two sensors.

AE signals were continuously acquired using a Mistras Micro‐SHM data acquisition system during mechanical testing. A minimum acquisition threshold of 40 dB was applied [114], and only signals with peak amplitudes exceeding this threshold were recorded. The recorded data were post‐processed for various outputs and analyzed using AEwin software (MISTRAS Group Inc.).

### 4.8 Scanning Electron Microscopy ‐ Focused Ion Beam Characterization

The microstructure of *Euplectella aspergillum* (EA) glass sponge was examined using a Focused Ion Beam‐Scanning Electron Microscope (FIB‐SEM, FEI Helios G3 DualBeam). SEM imaging was conducted for both unpolished and polished specimens. For the unpolished specimens (Figure 1C,D), sections of the cage skeletal structure were imaged without surface polishing. Prior to imaging, these samples were cut and coated with a 10nm iridium layer using a VCR IBS/TM200S Ion Beam Sputterer to ensure conductivity and reduce charging. For the polished specimens (Figure 1E–G), spicules from the skeletal framework were carefully cut, oven‐dried for 24 h at 60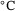, and embedded in low‐viscosity epoxy (Spurr) within cylindrical molds [115]. The molds were placed under vacuum at room temperature for 30 min to remove air bubbles from epoxy followed by curing for 24 h at 65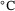 [115]. The epoxy‐embedded spicules were then sectioned using a diamond saw lubricated with isopropyl alcohol at 100 rpm to obtain cross‐sectional views. The exposed cross‐sections were sequentially polished using abrasive papers with grit sizes of 120, 180, 240, 320, 400, and 600, followed by diamond pastes of 6, 3, 1, and 0.25 μm. The polished surfaces were sputter‐coated with a 15 nm iridium layer using a Leica EM ACE600 sputter coater. After loading the specimen on SEM‐FIB system (FEI Helios G3 DualBeam), the spicules were located, and the FIB was used to mill the selected region to obtain clear cross‐sectional images.

### 4.9 Micro‐CT Analysis of Mortar‐Elastomer Interface

The mortar‐elastomer interfaces were examined using X‐ray micro‐computed tomography to assess interfacial continuity and porosity. Experimental details are provided in the Supporting Information, and representative cross‐sectional images of the mortar‐silicone and mortar‐polyurethane interfaces are shown in Figure S10.

### 4.10 Statistical Analysis

Data was presented as mean ± SD. A minimum of five repetitions was performed for each test. The one‐tailed F‐test and T‐test with a confidence interval of 95% were employed as a statistical method to study the significant difference between the variance and mean of the data, respectively. p<0.05 indicates a statistically significant difference between the samples. An α value of 0.05 was used. The Data Analysis Toolbox in Microsoft Excel was used to perform statistical analysis.

## Author Contributions

A.N. and R.M. conceived the project. A.N. conducted the numerical simulations. A.N., Z.L., S.G., K.D., and W.M. conducted the experiments. W.M. and R.M. developed the multi‐material printing process. W.M, K.D., Z.L, S.G., A.N., and R.M. developed the scripts for tool‐path generation. A.N. analyzed the data. A.N. and R.M. interpreted the data. A.N. wrote the original manuscript. A.N, R.M., and S.G. edited the manuscript. R.M. acquired funding.

## Conflicts of Interest

The authors declare no conflicts of interest.

## Supporting information


**Supporting File**: adma72960‐sup‐0001‐SuppMat.pdf.

## Data Availability

The data that support the findings of this study are available from the corresponding author upon reasonable request.
