# Supplementary material for: Tough, Ductile, and Strong Hard‐Soft Cementitious Composite Enabled by Multi‐Material Additive Manufacturing
Source: Adv Mater. 2026 Apr 9;38(45):e15461. doi: 10.1002/adma.202515461 (PMC13471887; doi:10.1002/adma.202515461)
Supplement: Supplementary file 1 [file ADMA-38-e15461-s001.pdf]

Supporting Information

Tough, Ductile, and Strong Hard-Soft Cementitious Composite Enabled by Multi-Material Additive Manufacturing

Aimane Najmeddine, Shashank Gupta, William Makinen, Zayvinn Lin, Krystal Delnoce, Reza Moini[1]

Aimane Najmeddine, Shashank Gupta, William Makinen, Zayvinn Lin, Krystal Delnoce, Reza Moini[1]

Department of Civil and Environmental Engineering, Princeton University, Princeton, NJ, USA

Email: Reza.Moini@princeton.edu

Multi-Material Additive Manufacturing

The custom-built multi-material printer (Figure S1A) is a versatile fabrication platform capable of depositing multiple materials with independent control. It supports asynchronous operation of distinct printing techniques, such as fused filament fabrication (FFF), direct ink writing (DIW), and elastomer/polymer extrusion, tailored to the requirements of individual materials like polymers, mortars, and silicones. By offering precise spatial control, the system supports the fabrication of architected composites that integrate materials with contrasting properties into unified, high-performance structures.

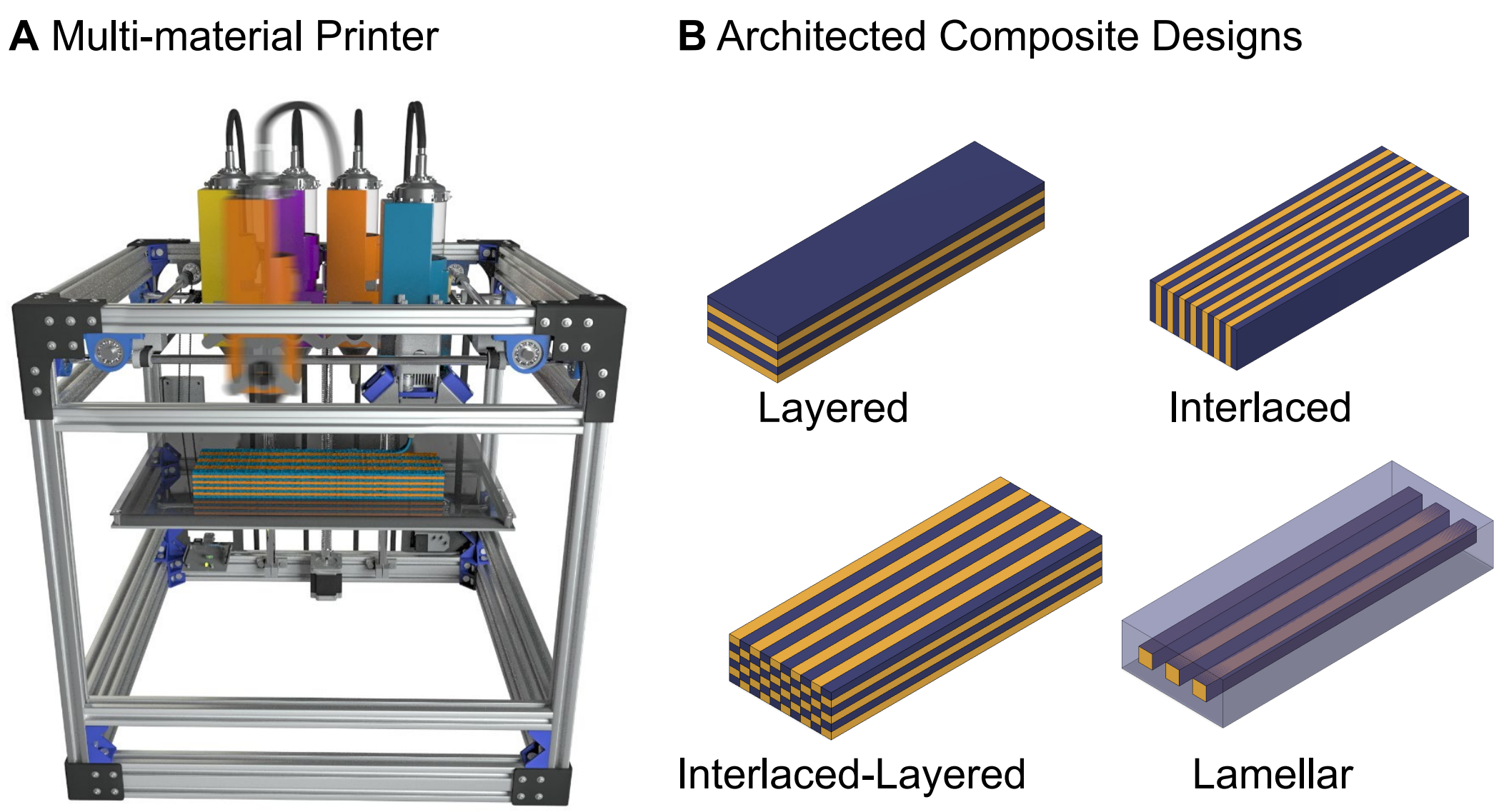

Figure S1: A. Custom-built multi-material extrusion printer capable of depositing multiple materials. B. Various architected composite designs enabled by this printer (e.g., layered, interlaced, interlaced-layered, and lamellar configurations), allowing precise control over spatial arrangement for engineering mechanical performance.

Employing the multi-material printer, the spatial arrangement of materials can be programmed at the mesoscale to realize layered, interlaced, lamellar, or hybrid designs (Figure S1B) to achieve specific mechanical functionalities. Such a high degree of control opens new avenues for structural optimization and multifunctionality in advanced material systems.

Multi-Material Printer Design, Operation, and Execution

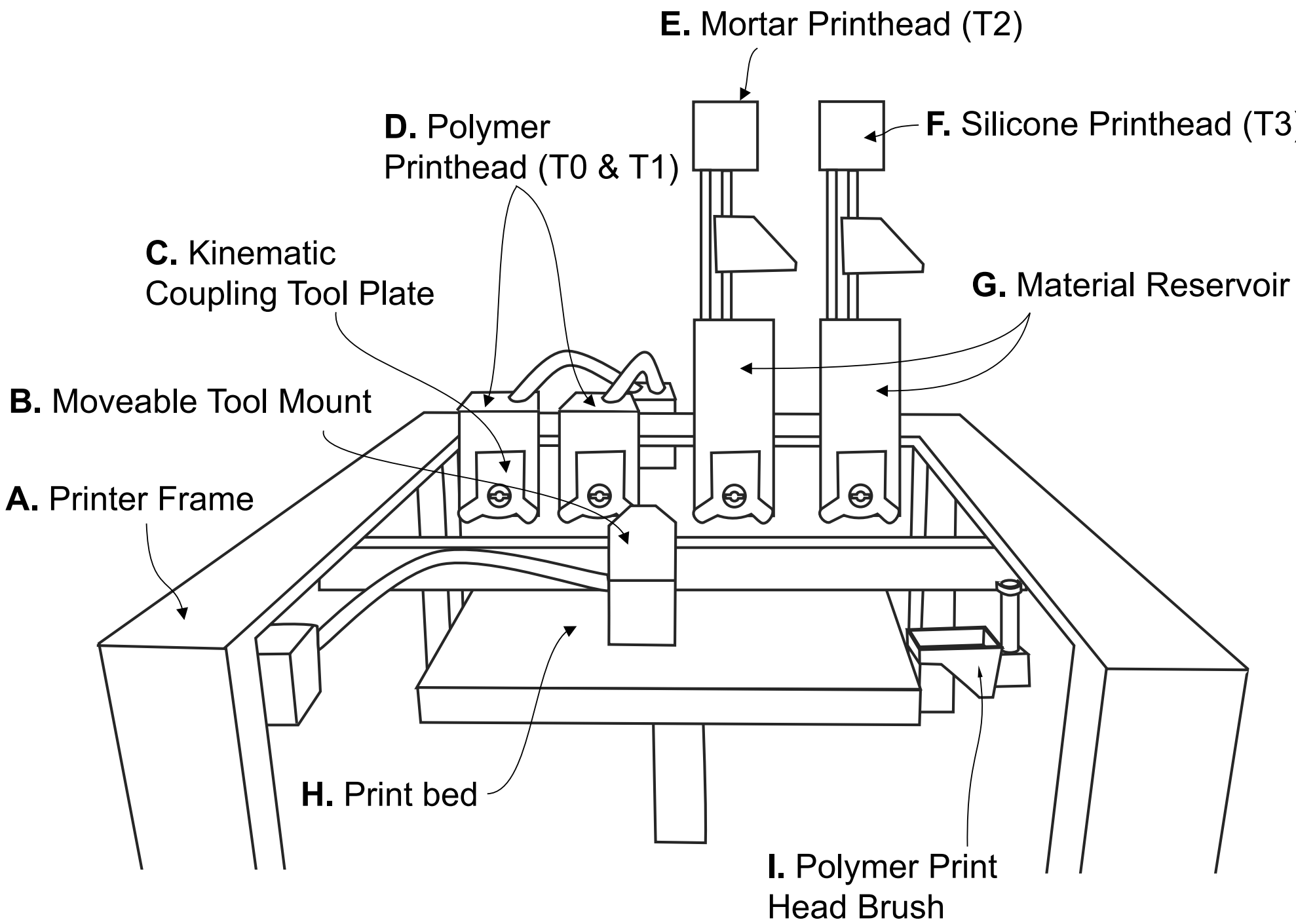

Figure S2: Schematic of the custom multi-material 3D-printing system demonstrating A. printer frame, B. moveable tool mount, C. kinematic coupling tool plate, D. polymer printhead (T0 & T1), E. mortar printhead (T2), F. silicone printhead (T3), G. material reservoir, H. print bed, and I. polymer printhead brush.

The multi-material 3D-printer is developed with the associated software, firmware, and tuning required to control the machine and fabricate the architected multi-material assembly. The multi-material system is built using an E3D ToolChanger [1], a tool-changing platform consisting of the frame (Figure S2A), motion system, and control electronics. The ToolChanger is engineered to allow for customizable movable tool mount (Figure S2B) that uses a kinematic coupling mechanism (Figure S2C) to select and park up to four different toolheads (Figure S2D-F). Each toolhead can be configured to deposit various materials, according to the multi-material assemble requirements.

The current system features polymer printheads (T0 and T1; Figure S2D), a mortar printhead (T2; Figure S2E), and a silicone printhead (T3; Figure S2F). Mortar and Silicone toolhead are each connected to dedicated material reservoirs (Figure S2G). The acrylic print bed (Figure S2H) serves as the substrate for material deposition, while a polymer printhead brush (Figure S2I) facilitates cleaning of polymer nozzles during tool changes.

While selected, the 'current' toolhead is activated to 3D-print using its respective 3D-printing technique and processing parameters according to the designed toolpath, with the other 'standby'

toolheads docked to the frame of the machine. When parked, a toolhead enters a 'standby' mode. When selecting a new toolhead, the 'current' one is first parked, with the tool mount then moving to pick up the next toolhead in the sequence, transitioning to become the new 'current' toolhead. In this work, the two main 3D-printing technologies used were syringe-based direct ink writing (DIW) [2, 3] of mortar and silicone, although the system is equipped and has been tested to fabricate using Fused Filament Fabrication (FFF) [4] with polymer.

The toolheads are designed by engineering Hyrel SDS-150XT extruder heads with custom 3D-printed components to accommodate large syringes – 150 ml for mortar and 290 ml for silicone – and to interface with the tool-changing system of E3D ToolChanger printer. Additional modifications are also made to the extruder body for both the mortar and silicone toolhead to allow interfacing with the tool parking mount and for the motor wires to be rerouted, ensuring that the wires would not impede the range of motion of the toolhead. The silicone toolhead required more significant changes, with the extruder body being enlarged to reinforce the entirety of the silicone tube walls. Lastly, motors with higher torque specifications are selected to facilitate reliable extrusion as the consistency of the mortar changes throughout the print.

Materials Formulation and Preparation

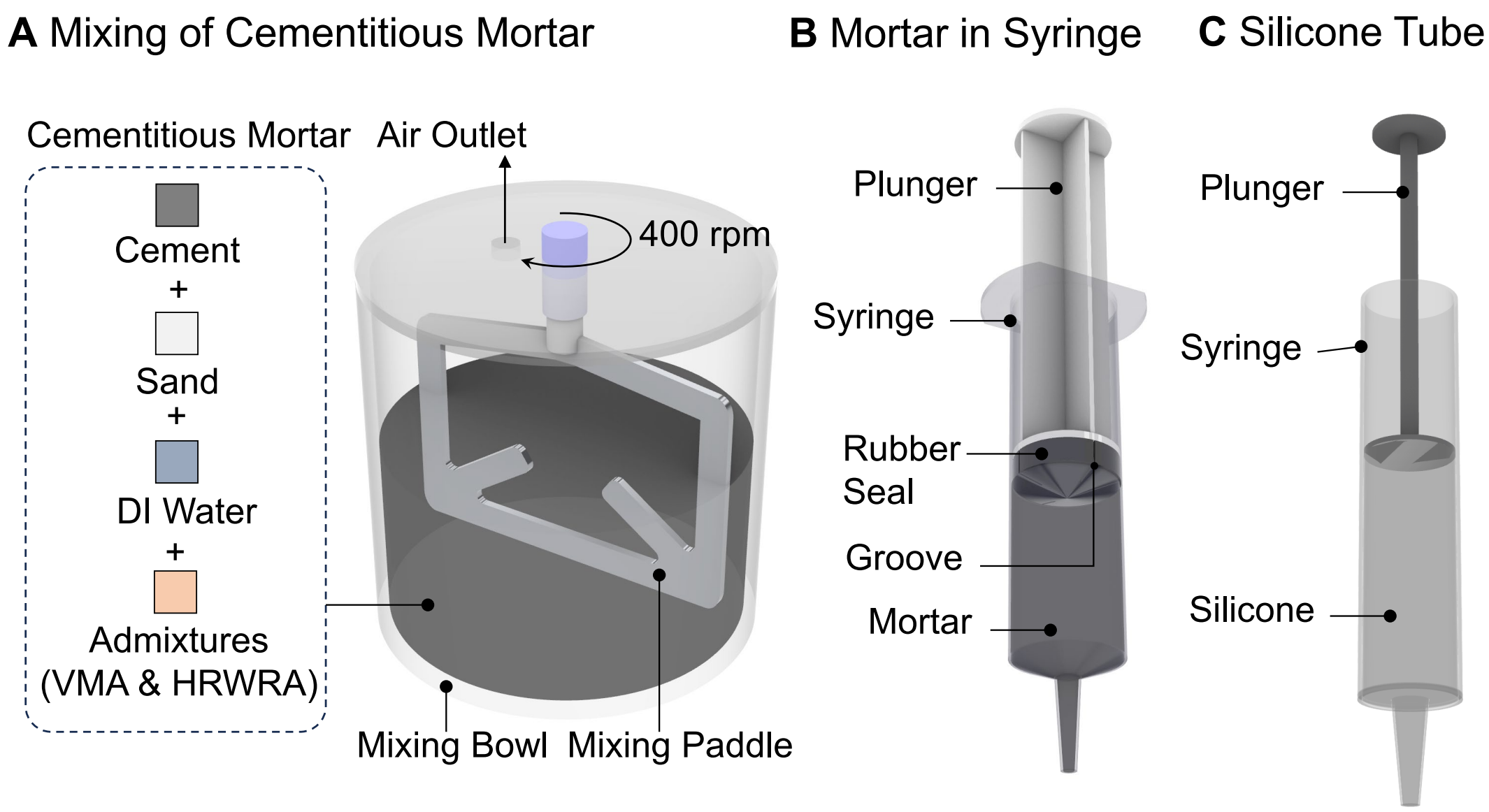

Figure S3: Schematic of material preparation. A. Mixing of cementitious mortar composed of cement, sand, deionized water, and admixtures (VMA and HRWRA) using a mixing paddle at 400 rpm, B. loading of mortar into a modified syringe equipped with a grooved plunger for the air escape, C. Silicone tube for extrusion-based applications.

Multi-Material Printer Control

The multi-material development requires a firmware to control the hardware mechanisms, take in sensor inputs, facilitate homing, and adjust other parameters such as flow rate and speed in-situ during the 3D-printing process. In addition, it requires software to generate the toolpaths that, when followed, generate architected multi-material assemblies. Together, these components constitute print control system and are subject to processing parameters depending on the toolhead in-use and the deployed material. The processing parameters include the following variables: (i) the thermal compatibility of different materials, (ii) the selection and parking toolheads of different materials considering their time-dependent material properties during standby and prime process before being selected, (iii) the mechanical specifications of each toolhead (e.g., torque, volume), and the limitations these places on the printing process and components, and (iv) tool-changing handling to take into account toolhead geometries, weight, and necessary material priming process. These control parameters have been accounted for in engineering the system for successful development.

Firmware of the Multi-Material Printing System

The control electronics for the 3D-printer consist of a Duet 2 control board, which uses a 32-bit microprocessor running RepRap Firmware (RRF) 3.0 [5]. This combination allows for several advantages in programing and control. RRF is a powerful control firmware stack for multi-tool 3D-printers, and it significantly enhances the versatility of the tool-changing platform through its user-defined macro-based customization and independent tool definitions. RRF supports a range of features not commonly available in other 3D-printer firmware, particularly with respect to RRF's advanced G-code and M-code commands.

G-code commands are commonly used to simply move a CNC device's toolhead to a specific location corresponding to an x, y, and z coordinate within the printer's build volume at a particular feed rate, with M-codes being used to control auxiliary parameters, such as tool temperatures, fan speeds, and accelerations. In RRF, however, the G-code and M-code commands more closely resemble a programming language. RRF is used here to interface with lower-level settings of the printer, with the ability to change parameters that would typically have to be configured by compiling and uploading a different firmware binary to the 3D-printer. RRF's G- and M-code commands can configure tool definitions, assign end stop pins, and even read sensor inputs.

Each Duet board supports a large number of inputs and outputs, with vast expansion possible through expansion boards. For the developed 3D-printer device, a DUEX 5 expansion board was used [6]. A hardware add-on to the DUET mainboard, the DUEX 5 has the capacity to support up to seven toolheads along with the necessary associated sensors (such as temperature sensors). Finally, the DUET board was controlled through a built-in web interface running on the DUET mainboard via RRF, making it very easy for the user to interact with the device over a network.

Automated Multi-Material Toolpath Generation Using Grasshopper

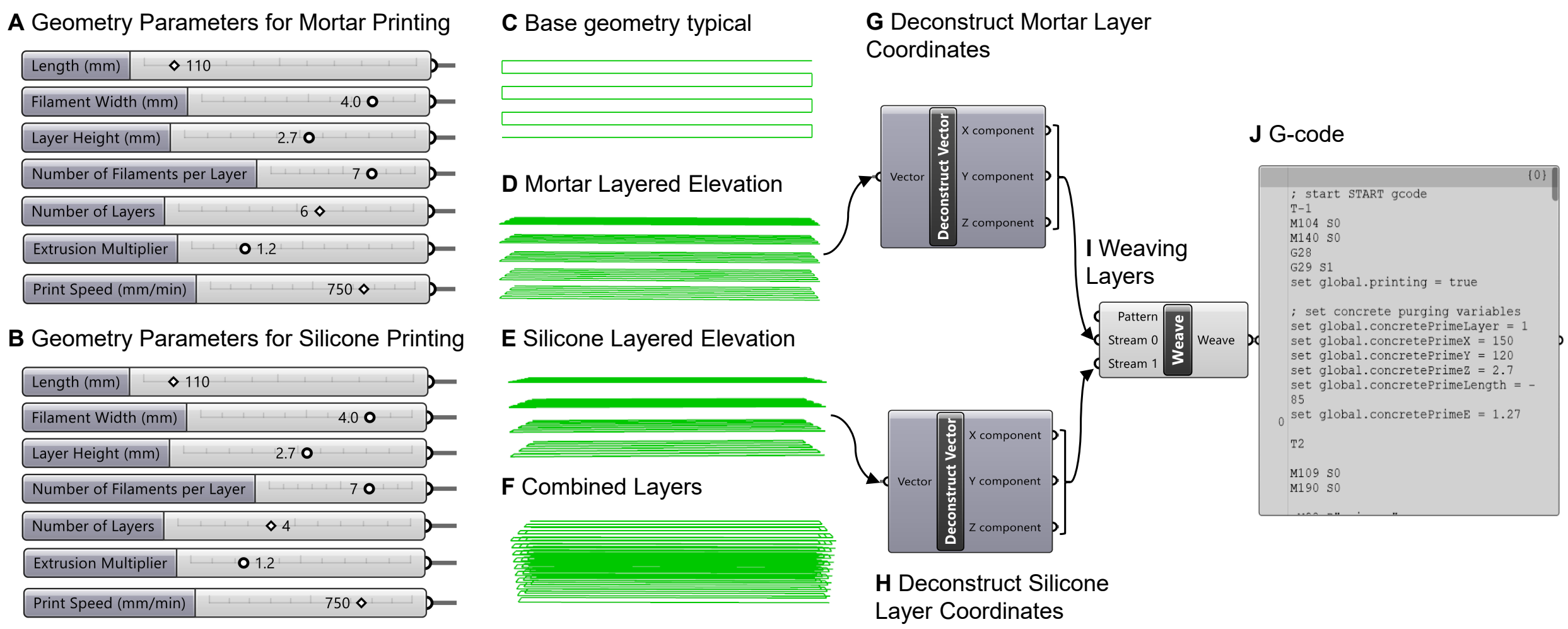

Figure S4: Workflow for automated generation of multi-material toolpaths using Grasshopper. A, B. Input parameters for mortar and silicone printing, respectively, including specimen length, filament width, layer height, number of filaments per layer, number of layers, extrusion multiplier, and print speed. C. Representative base geometry of a single layer derived from the defined parameters. D, E. Layered geometry generation for mortar and silicone, respectively. F. Combined multi-material geometry with alternating mortar and silicone layers. G, H. Deconstruction of mortar and silicone toolpaths into their respective X, Y, and Z coordinates. I. Interlacing (weaving) of the deconstructed coordinates to define the sequential multi-material deposition, resulting in J. the final G-code for multi-material printing.

Calculation of J-integral and R-curve

The resistance (R)-curve is evaluated by assessing the evolution of crack growth resistance, in terms of the J-integral ($J_{(i)}$), as a function of crack extension using a single-edge notch bend (SENB) test [7, 8, 9, 10]. The J-integral is evaluated using ASTM E1820 [7], where it is expressed as the sum of the elastic component (J_{el_i}) and the plastic component (J_{pl_i}):

$$J_{(i)} = J_{el_i} + J_{pl_i} \tag{1}$$

The elastic component J_{el_i} is calculated as a function of the stress intensity factor $K_{(i)}$, Young's modulus E, and Poisson's ratio ν:

$$J_{el_i} = \frac{K_{(i)}^2 \left(1 - \nu^2\right)}{E} \tag{2}$$

where the stress intensity factor $K_{(i)}$ is given by:

$$K_{(i)} = \left[\frac{P_i S}{B\, W^{3/2}}\right] f\left(\frac{a_i}{W}\right) \tag{3}$$

with B the specimen thickness, W the specimen depth, a_i the total crack length, P_i the load, and S the span length. The geometry factor $f(a_i/W)$ is defined as:

$$f\left(\frac{a_i}{W}\right) = \frac{3\left(\frac{a_i}{W}\right)^{1/2}\left[1.99 - \left(\frac{a_i}{W}\right)\left(1 - \frac{a_i}{W}\right)\left(2.15 - 3.93\left(\frac{a_i}{W}\right) + 2.7\left(\frac{a_i}{W}\right)^2\right)\right]}{2\left(1 + 2\frac{a_i}{W}\right)\left(1 - \frac{a_i}{W}\right)^{3/2}} \tag{4}$$

The plastic component J_{pl_i} is determined as:

$$J_{pl_i} = \left[J_{pl_{i-1}} + \left(\frac{\eta_{i-1}}{b_{i-1}}\right)\left(\frac{A_{pl_i} - A_{pl_{i-1}}}{B}\right)\right] \tag{5}$$

where B is the specimen thickness and b_i is the ligament depth, $b_i = W - a_i$, with W being the specimen depth and a_i the total crack length (sum of the intial notch length, a_0 and crack extension, Δa_i). The parameter η_i is a geometry-dependent factor (= 1.9 for SENB), and A_{pl_i} is the plastic area under the load versus CMOD curve corresponding to point i.

It is evident that the evaluation of the J-integral requires the determination of a_i. In this study, a_i is obtained from the compliance, calculated using the load-crack mouth opening distance (CMOD) data recorded during the SENB test [8].

The crack mouth opening displacement at step i (CMOD_i) during the SENB test is calculated as

$$\mathrm{CMOD}_i = \frac{6\, P_i\, a_i\, S}{W^2\, B\, E'}\, V_1\left(\frac{a_i}{W}\right), \tag{6}$$

where P_i is the applied load, a_i is the total crack length, W is the specimen depth, B is the specimen thickness, S is the span length, and E' is the effective Young's modulus. For plane strain conditions, $E' = E/(1-\nu^2)$, where E is the Young's modulus and ν is the Poisson's ratio.

The function $V_1(a_i/W)$ is a geometry factor defined as

$$V_1\left(\frac{a_i}{W}\right) = 0.76 - 2.28\left(\frac{a_i}{W}\right) + 3.87\left(\frac{a_i}{W}\right)^2 - 2.04\left(\frac{a_i}{W}\right)^3 + \frac{0.66}{\left[1-\left(\frac{a_i}{W}\right)\right]^2}. \tag{7}$$

Rearranging Equation (6), the effective Young's modulus E' can be expressed as

$$E' = \frac{6\,P_i\,a_i\,S}{W^2\,B\,\mathrm{CMOD}_i}\,V_1\left(\frac{a_i}{W}\right), \tag{8}$$

where S is the span length, P_i is the applied load, a_i is the total crack length, W is the specimen depth, B is the specimen thickness, CMOD_i is the crack mouth opening displacement, and $V_1(a_i/W)$ is the geometry factor defined in Equation (7).

The compliance at step i (C_i) is defined as

$$C_i\left(\frac{a_i}{W}\right) = \frac{\mathrm{CMOD}_i}{P_i}. \tag{9}$$

Substituting Equation (9) into Equation (8) gives

$$E' = \frac{6\,a_i\,S}{W^2\,B}\,\frac{1}{C_i\left(\frac{a_i}{W}\right)}\,V_1\left(\frac{a_i}{W}\right). \tag{10}$$

Since the effective Young's modulus E' is a material property, it remains constant during the test. Therefore, Equation (10) can be written for both the crack length at step i (a_i) and the initial notch length (a_0) as

$$E' = \frac{6\,a_i\,S}{W^2\,B}\,\frac{1}{C_i\left(\frac{a_i}{W}\right)}\,V_1\left(\frac{a_i}{W}\right) = \frac{6\,a_0\,S}{W^2\,B}\,\frac{1}{C_0\left(\frac{a_0}{W}\right)}\,V_1\left(\frac{a_0}{W}\right), \tag{11}$$

where a_0 is the initial notch length and C_0 is the initial compliance, obtained from the slope of the initial linear elastic portion of the load-CMOD curve.

Rearranging Equation (11) gives the crack length at the step i as

$$a_i = a_0\,\frac{C_i\left(\frac{a_i}{W}\right)}{C_0\left(\frac{a_0}{W}\right)}\,\frac{V_1\left(\frac{a_0}{W}\right)}{V_1\left(\frac{a_i}{W}\right)}, \tag{12}$$

By computing J-integral for each increment of the crack extension ($\Delta a = a_i - a_0$), the R-curve can be obtained.

Unified Phase-field Cohesive Zone Model (PF-CZM) Framework For Crack Propagation in Hard-Soft Materials with Interfaces

In the present work, we employ the large-deformation variational formulation developed by [11], which is capable of modeling crack propagation in layered assemblies composed of hard-soft constituents separated by interfacial zones. This formulation provides a comprehensive framework for analyzing crack propagation in layered materials by combining phase-field modeling of bulk fracture with a potential-based cohesive zone model to capture dissipation along the interface. In this framework, the solid body Ω_0 is divided into two regions (Ω_0^1 and Ω_0^2) by an interface Γ_0, with each region potentially exhibiting a different constitutive response (e.g., hard linear elastic ceramic or mortar, soft hyperelastic polymer), as illustrated in Figure S5.

The kinematics involve a motion mapping $\mathbf{x} = \chi(\mathbf{X}, t)$ from the reference to current configuration (Fig. S5A), with the displacement field defined as $\mathbf{u}(\mathbf{X}, \mathbf{t}) = \mathbf{x} - \mathbf{X}$ where $\mathbf{X}$ is the vector representing the location of an arbitrary material point in the domain of the initial (reference) configuration $\Omega_0 = \Omega_0^1 \bigcup \Omega_0^2$. The body is subject to body forces $\mathbf{b}_0$ and boundary conditions, including applied tractions $\mathbf{t}_0$ and prescribed displacements $\bar{\mathbf{u}}$.

The energy contribution due to the interface dissipation, Π^{int}, is accounted for within the total potential energy functional as follows:

$$\Pi(\mathbf{u}, d) = \Pi^{b^k}(\mathbf{u}, d) + \Pi^{\text{int}}(\mathbf{u}) + \mathcal{P}(\mathbf{u}) \tag{13}$$

where Π^{b^k} represents the bulk energy contributions with k referring to the bulk material type considered (i.e., hard or soft), and $\mathcal{P}$ represents external work.

For the bulk contribution to the total potential in Eq. 13, the phase-field variational formulation is used, wherein $\Pi^{b^k}(\mathbf{u}, d)$ is given by:

$$\Pi^{b^k}(\mathbf{u}, d) = \int_{\Omega_0^k} \Psi^k(\mathbf{F}, d)\mathrm{dV} + \int_{\Omega_0^k} G_c^k \gamma_{(d,\nabla d)}\mathrm{dV} \tag{14}$$

where $\Psi^k(\mathbf{F}, d)$ and G_c^k are the Helmholtz free energy and the fracture toughness of bulk material k, respectively, and the crack surface density function is given by the following expression:

$$\gamma_{(d,\nabla d)} = \frac{1}{c_\phi}\left[\frac{1}{l_c^k}\phi(d) + l_c^k \nabla d \cdot \nabla d\right] \tag{15}$$

where $c_\phi = 4\int_0^1 \sqrt{\phi_{(\beta)}}\mathrm{d}\beta$ and $\phi(d) = d^2$ (AT2) [12].

As for the interface, the PPR cohesive potential function $\varphi^{\text{int}}(\Delta\mathbf{u})$, defined in terms of the dis-

placement jump across the interface $\Delta\mathbf{u} = (\Delta\mathbf{u}_t, \Delta\mathbf{u}_n)^\mathsf{T}$, is used [13]:

$$\begin{aligned}\varphi^{\text{int}}(\Delta_n, \Delta_t) =& \min(\mathcal{G}_n, \mathcal{G}_t) + \left[\Gamma_n\left(1 - \frac{\Delta_n}{\delta_n}\right)^{\alpha}\left(\frac{m}{\alpha} + \frac{\Delta_n}{\delta_n}\right)^{m} + \langle\mathcal{G}_n - \mathcal{G}_t\rangle\right]\times \\ & \left[\Gamma_t\left(1 - \frac{|\Delta_t|}{\delta_t}\right)^{\beta}\left(\frac{n}{\beta} + \frac{|\Delta_t|}{\delta_t}\right)^{n} + \langle\mathcal{G}_t - \mathcal{G}_n\rangle\right]\end{aligned} \tag{16}$$

where $\mathcal{G}_n$ and $\mathcal{G}_t$ are Mode-I and Mode-II fracture energies, Γ_n and Γ_t are energy constants, m and n are exponents of the PPR CZM model, α and β are shape parameters that control the softening behavior: $\alpha, \beta < 2$ produces concave traction-separation curves, while $\alpha, \beta \gg 2$ yields convex curves typical of quasi-brittle materials; and δ_n and δ_t are the final crack openings in the normal and tangential directions, respectively, related to the critical normal and tangential crack openings, δ_{tc} and δ_{nc}, as follows: $\delta_{tc} = \lambda_t\delta_t$ and $\delta_{nc} = \lambda_n\delta_n$, respectively.

The normal and tangential cohesive tractions, T_n and T_t, are derived from the potential, Eq. 16 as follows:

$$\begin{aligned}T_n =& \frac{\Gamma_n}{\delta_n}\left[m\left(1 - \frac{\Delta_n}{\delta_n}\right)^{\alpha}\left(\frac{m}{\alpha} + \frac{\Delta_n}{\delta_n}\right)^{m-1} - r\left(1 - \frac{\Delta_n}{\delta_n}\right)^{\alpha-1}\left(\frac{m}{\alpha} + \frac{\Delta_n}{\delta_n}\right)^{m}\right]\times \\ & \left[\Gamma_t\left(1 - \frac{|\Delta_t|}{\delta_t}\right)^{\beta}\left(\frac{n}{\beta} + \frac{|\Delta_t|}{\delta_t}\right)^{n} + \langle\mathcal{G}_t - \mathcal{G}_n\rangle\right]\end{aligned} \tag{17}$$

$$\begin{aligned}T_t =& \frac{\Gamma_t}{\delta_t}\left[n\left(1 - \frac{|\Delta_t|}{\delta_t}\right)^{\beta}\left(\frac{n}{\beta} + \frac{|\Delta_t|}{\delta_t}\right)^{n-1} - \beta\left(1 - \frac{|\Delta_t|}{\delta_t}\right)^{\beta-1}\left(\frac{n}{\beta} + \frac{|\Delta_t|}{\delta_t}\right)^{n}\right]\times \\ & \left[\Gamma_n\left(1 - \frac{\Delta_n}{\delta_n}\right)^{\alpha}\left(\frac{m}{\alpha} + \frac{\Delta_n}{\delta_n}\right)^{m} + \langle\mathcal{G}_n - \mathcal{G}_t\rangle\right]\frac{\Delta_t}{|\Delta_t|}\end{aligned} \tag{18}$$

The normal and tangential traction-separation curves reach their maximum when the normal and tangential separations reach critical crack opening widths δ_{nc} and δ_{tc}, respectively:

$$\left.\frac{\partial T_n}{\partial \Delta_n}\right|_{\Delta_n=\delta_{nc}} = 0, \quad \left.\frac{\partial T_t}{\partial \Delta_t}\right|_{\Delta_t=\delta_{tc}} = 0 \tag{19}$$

At these critical points, the maximum tractions correspond to the cohesive strengths:

$$T_n(\delta_{nc}, 0) = \sigma_{max}, \quad T_t(0, \delta_{tc}) = \tau_{max} \tag{20}$$

The contribution to the total potential Eq. 13 is then given by the potential function $\varphi^{\text{int}}(\Delta_n, \Delta_t)$, integrated over the domain Γ_0:

$$\Pi^{\text{int}}(\Delta\mathbf{u}) = \int_{\Gamma_0} \varphi^{\text{int}}(\Delta\mathbf{u})\mathrm{dA} \tag{21}$$

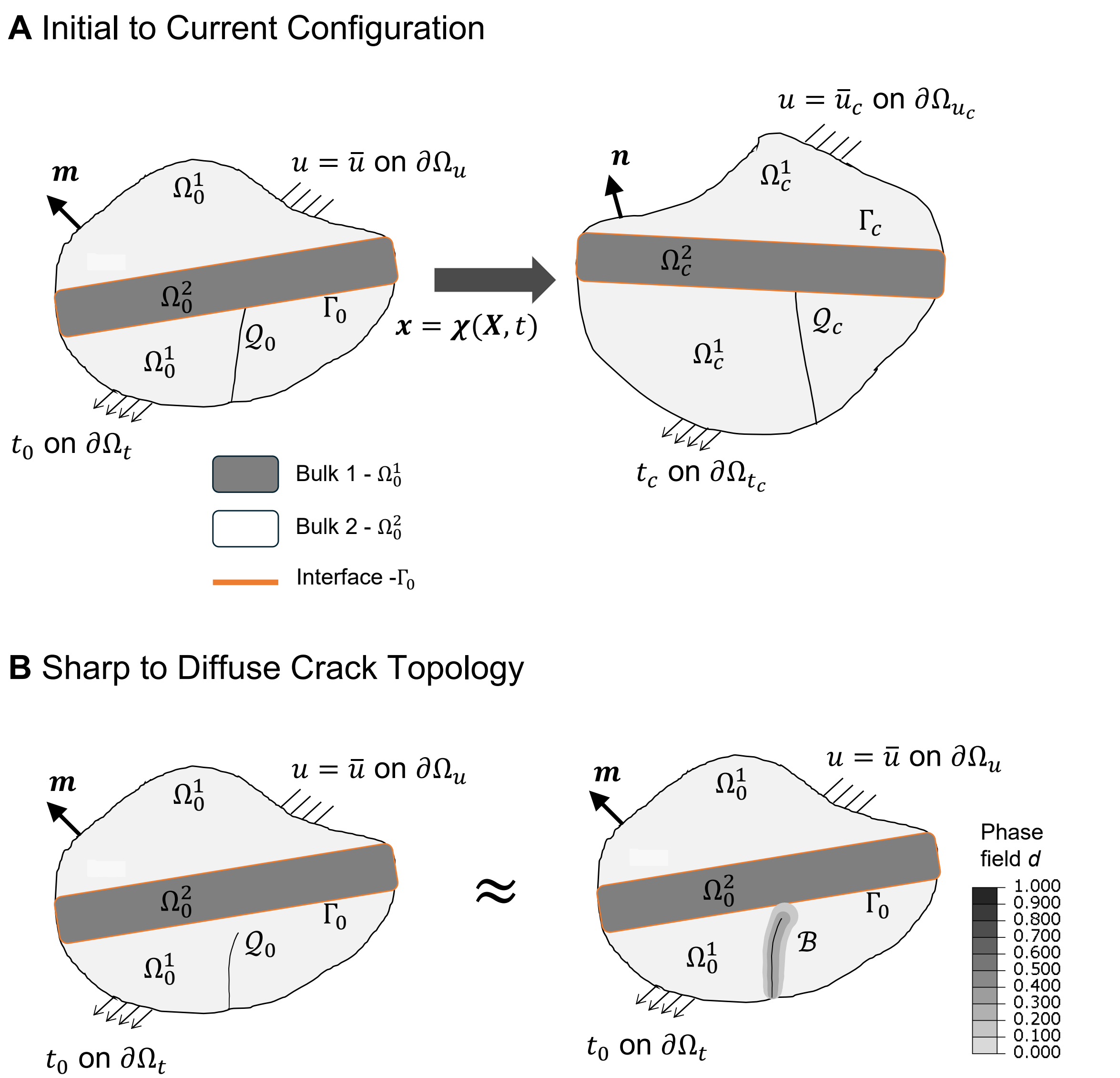

Figure S5: A. Kinematics of a solid body containing an initial crack $\mathcal{Q}_0$ and an interface Γ_0 separating two regions Ω_0^1 and Ω_0^2 in the initial and Ω_c^1 and Ω_c^2 in the current configurations. B. Substitution of the sharp crack topology $\mathcal{Q}_0$ with a diffuse representation $\mathcal{B}$ having a finite width over which a scalar phase-field variable varies from 0 (intact) to 1 (complete fracture).

The equilibrium of the system is determined through the principle of stationary (or minimum potential) energy, requiring that the variation of the total potential energy vanishes:

$$\delta\Pi = \delta\Pi^{b^k} + \delta\Pi^{\text{int}} + \delta\mathcal{P} = 0 \tag{22}$$

The variational expressions with respect to the displacement and phase-field variable are given by:

$$\delta\Pi_u^{b^k} = \int_{\Omega_0^k} \frac{\partial\Psi^k(\mathbf{F},d)}{\partial\mathbf{F}}\frac{\partial\delta\mathbf{u}}{\partial\mathbf{X}}\mathrm{dV}, \quad \delta\Pi_d^{b^k} = \int_{\Omega_0^k} \frac{\partial\Psi^k(\mathbf{F},d)}{\partial d}\delta d\mathrm{dV} + \int_{\Omega_0^k} \frac{G_c^k}{c_\phi l_c}\phi'_{(d)}\delta d\mathrm{dV} - \int_{\Omega_0^k} \frac{2G_c^k l_c}{c_\phi}\Delta d\delta d\mathrm{dV} \tag{23}$$

where $\Delta d = \nabla\cdot\nabla d$ is the Laplacian of the phase-field variable; whereas for the interface, the variation takes the following form:

$$\delta\Pi^{\mathrm{int}} = \delta\mathbf{u}^{\mathsf{T}}\int_{\Gamma_0}\left(\frac{\partial\Delta\mathbf{u}}{\partial\mathbf{u}}\right)^{\mathsf{T}}\mathbf{T}\mathrm{dA} \tag{24}$$

where $\mathbf{T}$ is the first Piola-Kirchhoff cohesive traction vector (T_t, T_n) , where T_n and T_t are given by Equation. 17 and 18:

$$\mathbf{T} = \frac{\partial\varphi^{\mathrm{int}}(\Delta\mathbf{u})}{\partial\Delta\mathbf{u}} = (T_t, T_n)^{\mathsf{T}} \tag{25}$$

The coupled large deformation phase-field PPR CZM framework has been implemented in Abaqus via a UEL subroutine to examine crack propagation [11].

The following bulk properties were used for the effect of soft material geometry numerical study (Figure 5):

Table 1: Bulk material properties for mortar and elastomers used in the PF-CZM numerical simulation (Figure 5 & 6)

Material	E (MPa)	ν	G_c (N/mm)	l_c (mm)
Mortar	8803	0.22	0.02	0.1
Elastomer (Silicone)	0.3	0.49	0.86	0.1

Table 2: Interface properties for mortar-elastomer interfaces used in the PF-CZM numerical simulation (Figure 5 & 6)

Interface Type	$\mathcal{G}_n$ (N/mm)	$\mathcal{G}_t$ (N/mm)	σ_{max} (MPa)	τ_{max} (MPa)
Mortar-Silicone	0.30	3.0	0.18	1.8

The following properties were used for the effect of soft material properties study (Figure 6):

The elastomer's Young modulus was calculated using $E_e = 2G_e(1+\nu)$ where G_e is the elastomer rubber (shear) modulus, and ν is Poisson's ratio (assumed to be equal to 0.49, typical of incompressible elastomeric materials). Note that the mortar properties were kept constant and only the

Table 3: Material and interface properties used in parametric elastomer stiffness study (Figure 6)

Case		Bulk				Interface			
		E (MPa)	ν	G_c (N/mm)	l_c (mm)	$\mathcal{G}_n$ (N/mm)	$\mathcal{G}_t$ (N/mm)	σ_{max} (MPa)	τ_{max} (MPa)
Case I	Mortar	8800	0.22	0.02	0.1	0.3	3	0.18	1.8
	Elastomer	**0.3**	0.49	0.86	0.1				
Case II	Mortar	8803	0.22	0.02	0.1	0.3	3	0.18	1.8
	Elastomer	**2.98**	0.49	0.86	0.1				
Case III	Mortar	8803	0.22	0.02	0.1	0.3	3	0.18	1.8
	Elastomer	**14.9**	0.49	0.86	0.1				
Case IV	Mortar	8803	0.22	0.02	0.1	0.3	3	0.18	1.8
	Elastomer	**29.8**	0.49	0.86	0.1				

stiffness of the soft elastomer was changed. Note that in all simulations performed in this work, the following values were used for the PPR parameters: $\alpha = 4$, $\beta = 4$, $\lambda_n = 0.03$, $\lambda_t = 0.03$.

Modulus of Rupture (MOR) and Work-of-fracture enhancements as a function of elastomer thickness in the Mortar-silicone (Mortar-SI) layered configurations

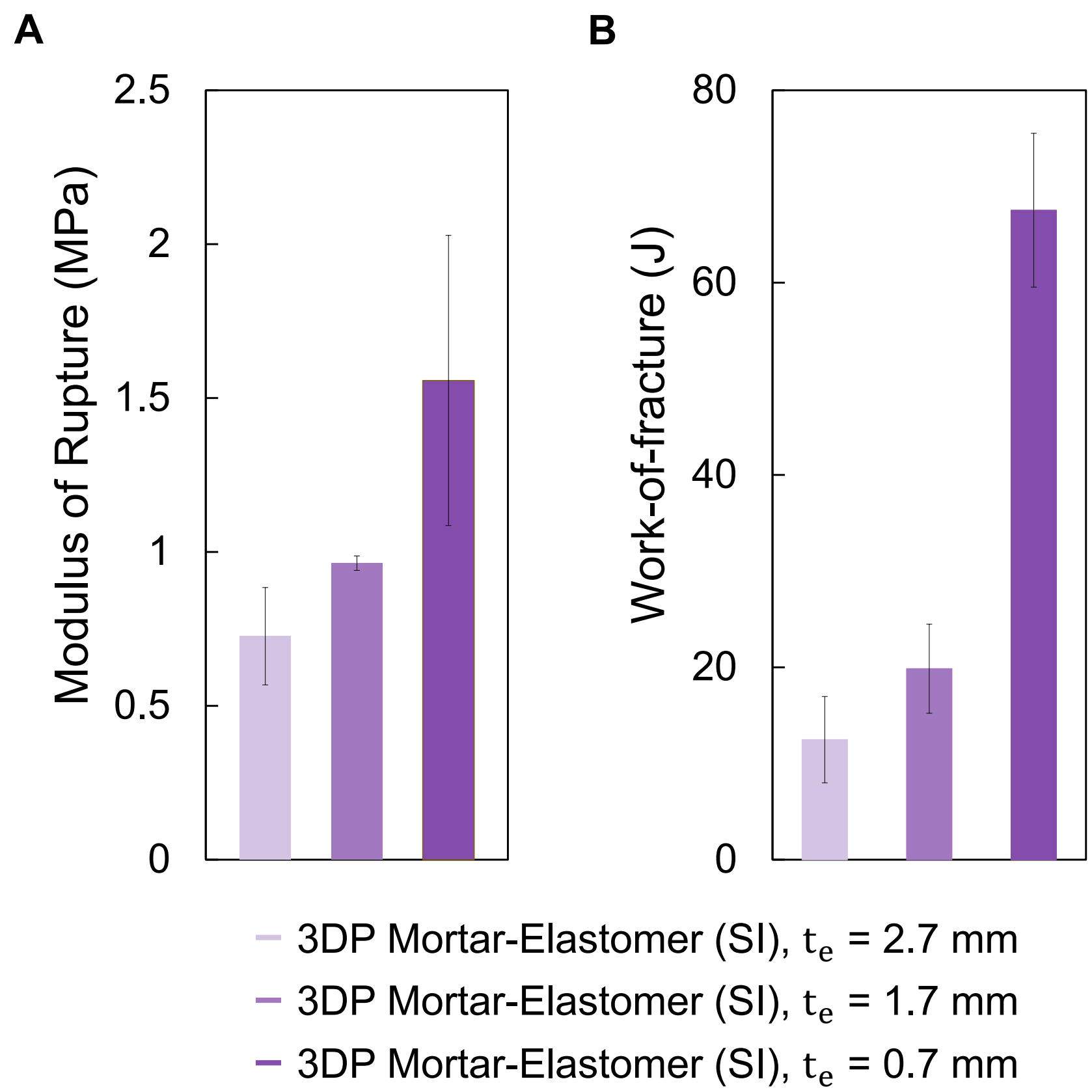

Figure S6: A. Modulus of rupture (MOR) and B. Work-of-fracture for the three Mortar-SI configurations with varying soft layer thicknesses.

Mechanical Characteristics of Bulk Hard (Mortar) and Soft (Silicone, Polyurethane) and their Interfaces

Mechanical Properties of Mortar

The fracture toughness (G_c^m) was determined based on critical stress intensity factor (K_{IC}) (Eq. 3). The Young's modulus was determined from Eq. 10. The Poisson's ratio was obtained based on literature from [14]. The calculated values are reported in Table 3.

Mechanical Properties of Elastomers: Silicone (SI) and Polyurethane (PU)

The fracture toughness of the two elastomers used in this work (i.e., SI and PU) was measured using the tear (trouser) test [15]. For each material, specimens consisting of a rectangular strip (width 15 mm, length 150 mm, thickness $t_s = 2$ mm) with a longitudinal pre-cut (notch) of 40 mm were prepared. Tensile tests were then performed for each specimen using an MTS Criterion C45.305 with tensile clamps at a constant displacement rate of 50 mm/min in accordance with ASTM-D624 [16]. The fracture toughness was calculated as:

$$G_c^e = \frac{2F_{\text{avg}}}{t_s}, \tag{26}$$

where F_{avg} is the average tearing force in the plateau region and t_s is the sample thickness. To prevent crack path deflection toward specimen edges, the sides of the specimen were reinforced with Kapton polyimide tape, whose stiffness is negligible. Elastomer sheets were cast in molds and cured for 7 days before cutting. Five replicates were tested at room temperature, and the point of tear was taken as the onset of catastrophic rupture [15].

The tensile stress-strain response for the two elastomers (i.e., SI and PU) was obtained by performing a direct-tension test on dog-bone specimens in accordance with ASTM D412-16 [17]. Each specimen had an overall length of 115 mm, width of 25 mm, and a thickness of 2 mm, with a neck length of 33 mm and a width of 6 mm. The tests were conducted under displacement control at 500 mm/min until failure, and four replicates were tested. The shear modulus was then obtained by fitting a simple Neo-Hookean hyperelastic constitutive formulation [18].

The measured fracture toughness and shear modulus corresponding to each of the two elastomers are illustrated in Figure S7A, and C, respectively.

Mechanical Properties of Interfaces in Mortar-Silicone and Mortar-Polyurethane Multi-materials

The interface strength between hardened mortar and elastomer (silicone or polyurethane) was evaluated in flexure and shear using the three-point bending test and the antisymmetric four-point

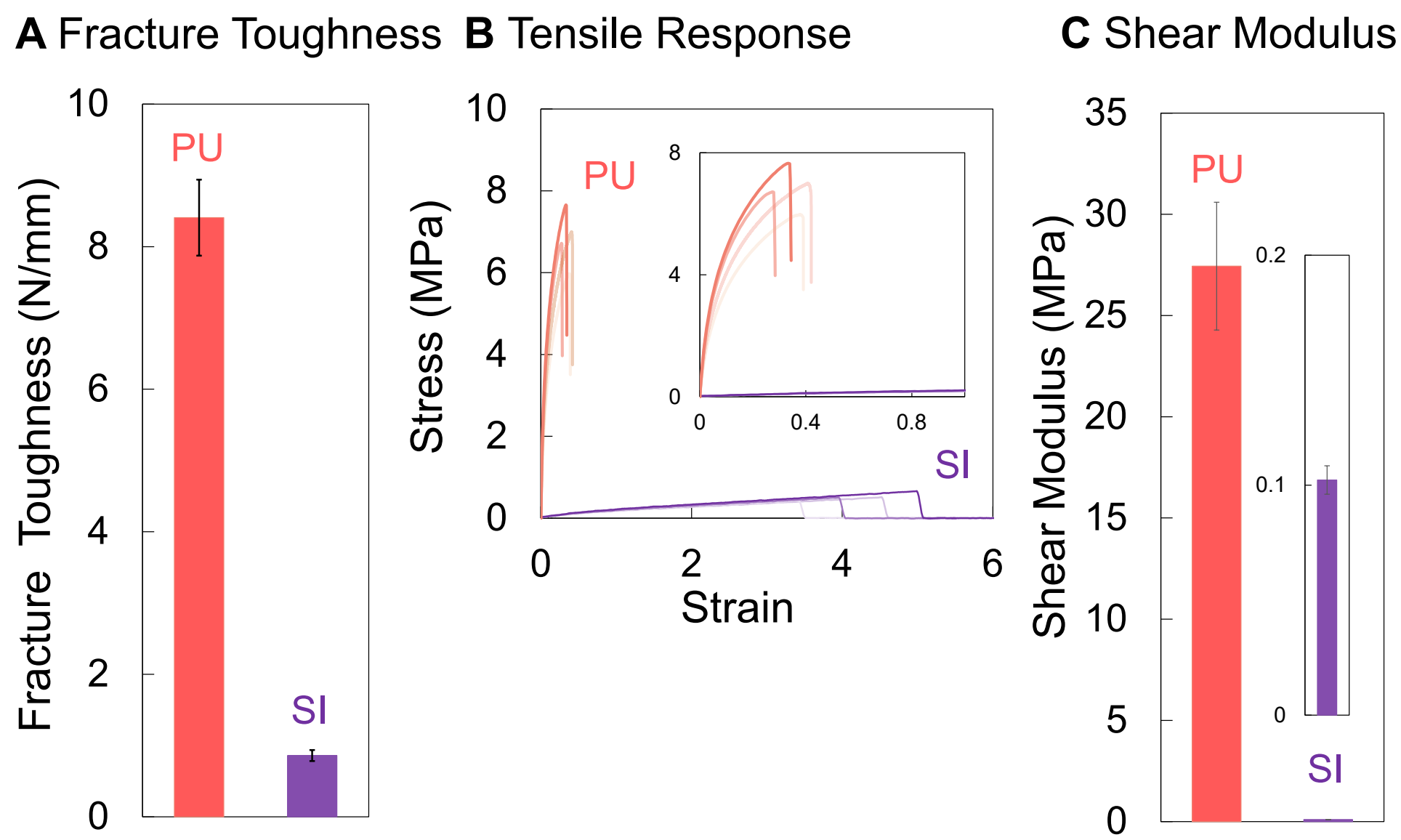

Figure S7: A. Fracture toughness, B. Stress-strain response under tension, and C. Shear modulus of the elastomeric materials (polyurethane (PU) and silicone (SI)) materials used in this work.

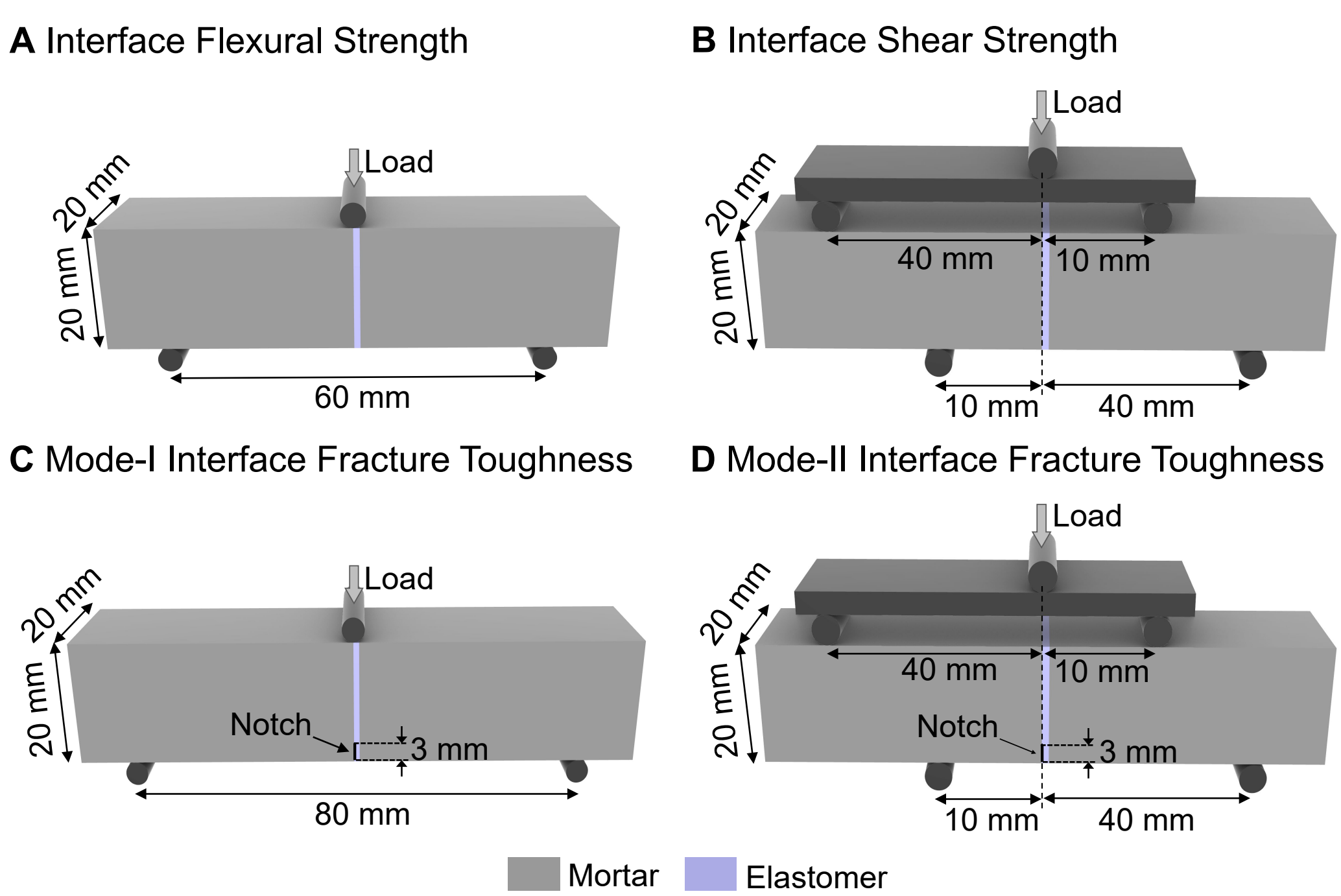

Figure S8: Schematics of the four mechanical tests used to characterize the mortar-elastomer interface. A. Three-point bending for interface flexural strength. B. Antisymmetric four-point bending for interface shear strength. C. SENB configuration for Mode I interfacial fracture toughness. D. Notched antisymmetric four-point bending for Mode II interfacial fracture toughness. Mortar and elastomer are shown in gray and light purple, respectively.

bending test, respectively (Figure S8A and S8B) [19, 20].

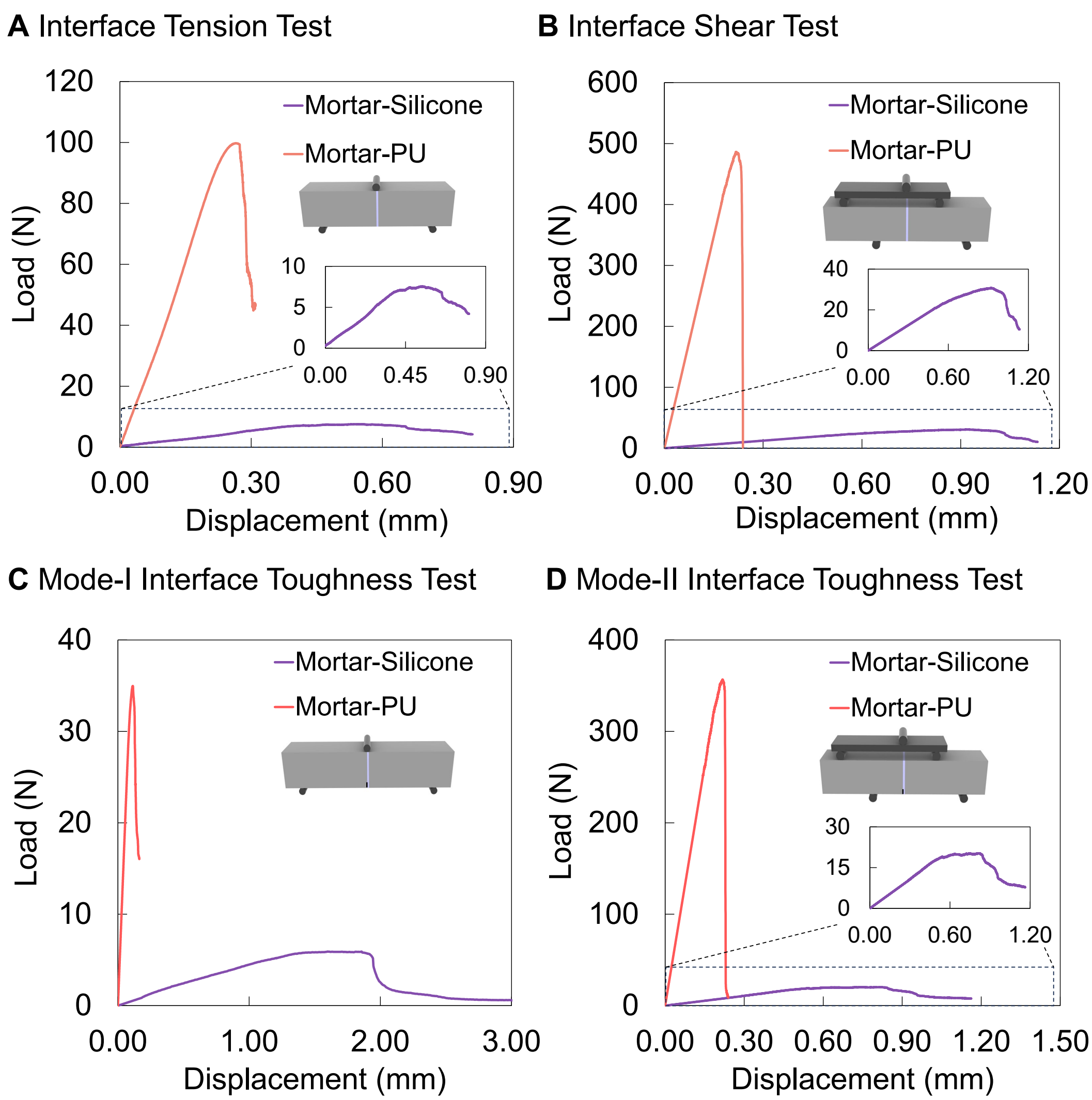

Figure S9: Load-displacement responses of mortar-elastomer interfaces obtained from A. interface tensile test using three-point bending (3PB), B. interface shear test using antisymmetric four-point bending, C. Mode I interfacial fracture toughness test using the SENB test, and D. Mode II interfacial fracture toughness test using notched antisymmetric four-point bending. Across all loading configurations, the mortar-PU interface exhibits a substantially higher load-bearing capacity than the mortar-silicone interface, exceeding it by factors of 11.9× (tension test), 15.9× (shear test), 7.2× (Mode I fracture toughness test), and 20.1× (Mode II fracture toughness test). Purple refers to Mortar-SI and Red refers to the Mortar-PU.

For the three-point bending test, an approximately 1 mm elastomer layer was placed between two mortar prisms measuring 40 mm × 20 mm × 20 mm (Figure S8A). Similarly, for the antisymmetric four-point bending test, approximately 1 mm elastomer layer was placed between two mortar prisms of dimensions 50 mm × 20 mm × 20 mm. The upper and lower rollers were arranged in an antisymmetric configuration with distances $S_1 = 10$ mm and $S_2 = 40$ mm from the midspan to the inner and outer rollers, respectively, on either side of the beam (Figure S8B). This configuration

eliminates bending moments at the interface and induces pure shear.

Fracture toughness of the interface was characterized under Mode I and Mode II loading using single-edge notch bend (SENB) and notched antisymmetric four-point bending configurations, respectively (Figure S8C and S8D) [7, 21]. In the SENB setup, a span length (S) of 80 mm, depth (W) of 20 mm, and thickness (W) of 20 mm were used, with a notch length of 3 mm (Figure S8C). In the notched antisymmetric four-point bending configuration, the specimen dimensions were identical to those of the unnotched specimens, with a 3 mm notch introduced at the interface to promote Mode II fracture (Figure S8D).

Figure S9 summarizes the load-displacement behavior of the mortar-elastomer interfaces across four test configurations: interface tension, interface shear, Mode I fracture toughness, and Mode II fracture toughness.

<u>**Micro-CT Analysis of Mortar-Elastomer Interface**</u>

X-ray micro-computed tomography was performed using a Zeiss Xradia 520 Versa X-ray microscope with a 0.4× objective. For the mortar-silicone samples, scans were conducted at 110 kV and 15.5 W using a HE2 source filter, while the mortar-polyurethane (PU) samples were scanned at 140 kV and 21 W using a HE4 source filter. In both cases, an exposure time of 1 s, binning of 2, and a full 360° rotation were employed. The resulting voxel resolutions were 33.75 μm per pixel for the mortar-silicone samples and 48.45 μm per pixel for the Mortar-PU samples.

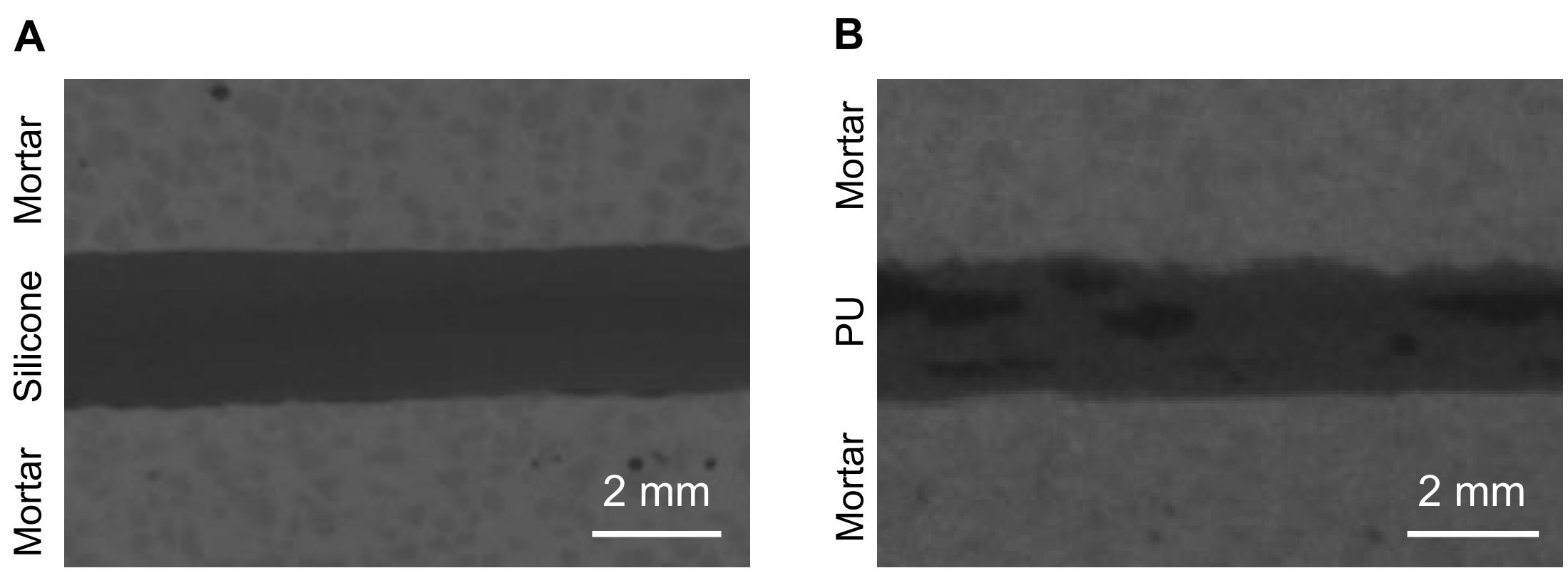

Figure S10: X-ray micro-computed tomography at a typical cross-sections of mortar-elastomer interfaces. A. mortar-silicone layer (with resolution of 33.75 μm/pixel) and B. mortar-polyurethane (PU) layer (with resolution of 48.45 μm/pixel).

Figure S10 presents X-ray micro-computed tomography cross-sections of mortar-elastomer interfaces. Figure S10A shows the mortar-silicone specimen, where a continuous silicone layer is visible between two mortar regions. The silicone layer appears uniform in thickness with a smooth adherence to the surrounding mortar. The silicone layer does not exhibit any visible pores at this resolution (33.75 μm/pixel). Figure S10B shows the mortar-polyurethane (PU) specimen. The PU layer shows the presence of pores, indicated by localized darker regions. Both the silicone and PU interfaces with mortar do not show the presence of large pores, indicating continuous contact and adherence with the mortar.

Digital Image Correlation (DIC) Crack Evolution in Thin-layered Mortar-SI and Mortar-PU systems

Figure S11 presents representative DIC major-principal-strain contours for the thin-layer Mortar-SI (0.7 mm) architecture, synchronized with the marked points (i-ix) on the force-displacement response. Consistent with the mechanisms discussed in the main text for the 2.7 mm case, crack evolution proceeds in a staged manner: strain localizes first at the notch tip and advances through the initial mortar layer(s), followed by repeated arrest/deflection at mortar-silicone interfaces and subsequent re-nucleation in the next mortar layer. The discrete load drops and subsequent re-hardening segments correspond to these successive mortar-layer fracture events, while the silicone layers remain largely intact and provide bridging. Compared to the PU system (below), the Mortar-SI (0.7 mm) specimen exhibits a smaller range of deformation and a more abrupt terminal drop once the remaining load path is exhausted, reflecting the relatively compliant nature of silicone and the smaller extent of sustained post-peak load transfer at this stiffness level.

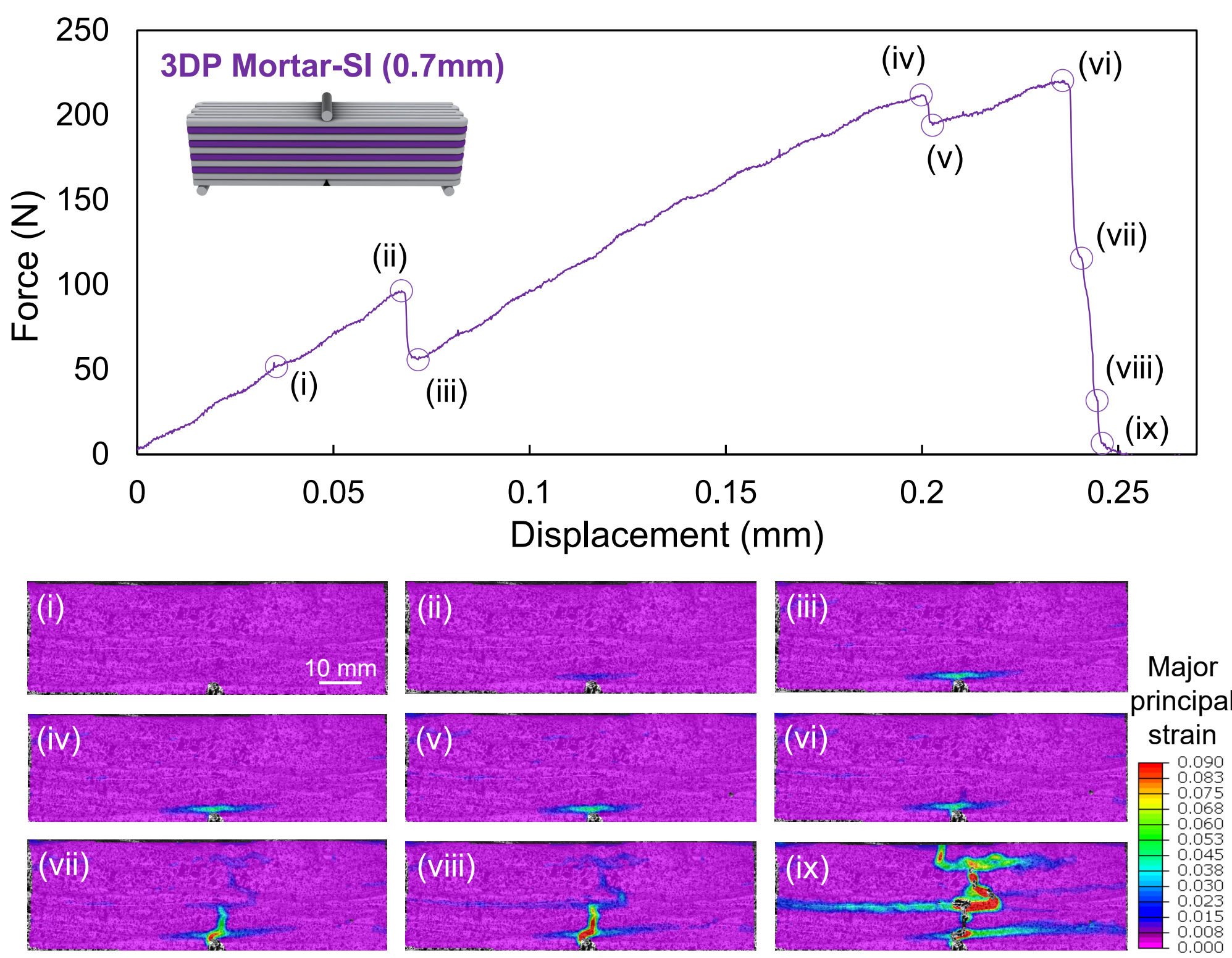

Figure S11: DIC-based crack evolution in 3DP Mortar-SI (0.7 mm) layered architecture under SENB. Major principal strain contours at selected stages (i-ix) are shown above the corresponding force-displacement plot. The sequence highlights staged crack growth through mortar layers with repeated arrest, deflection at mortar-silicone interfaces, and re-nucleation in subsequent mortar layers. The silicone layers remain largely intact and bridge across cracked mortar segments, enabling multi-stage load drops followed by re-hardening, and culminating in a terminal drop once the remaining load-carrying capacity is exhausted. Scale bar: 10 mm.

Figure S12 shows the corresponding DIC strain evolution for Mortar-PU (0.7 mm), with frames (i-vi) mapped to the labeled points on the force-displacement curve. The early stages capture notch-driven crack initiation and upward propagation through mortar, followed by pronounced crack deflection along a mortar-elastomer interface and sustained bridging as the elastomer layer continues to transfer load between cracked mortar segments. In contrast to the silicone case, the PU-based architecture maintains substantial load over a much larger displacement range, manifested by a long, stable post-peak response with progressive load decay rather than catastrophic loss of capacity. The DIC fields corroborate that the crack path becomes increasingly tortuous after the primary mortar cracking events, consistent with elastomer-enabled load redistribution and continued energy dissipation through interfacial processes and elastomer deformation during the post-mortar regime.

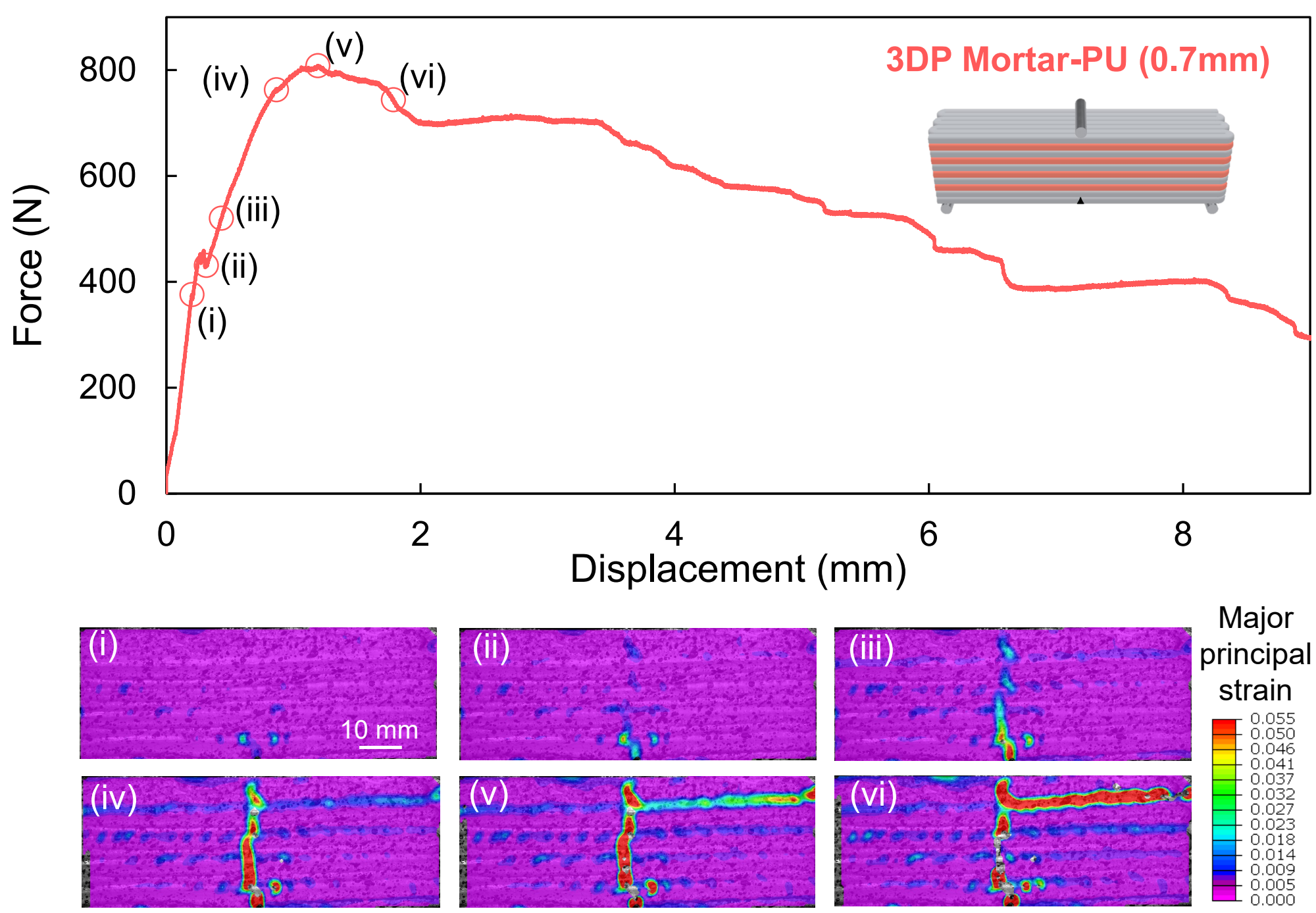

Figure S12: DIC-based crack evolution in 3DP Mortar-PU (0.7 mm) layered architecture under loading. Major principal strain contours at selected stages (i-vi) are shown above the corresponding force-displacement plot. The sequence captures fracture initiated at the notch in the mortar layer, followed by pronounced crack deflection along a mortar-elastomer interface and sustained bridging by the polyurethane layer, resulting in extended stable post-peak load transfer over large displacement, and an ultimate interfacial failure. The evolving strain fields during the gradual post-peak response indicate increasing deformation and distributed dissipation across soft material layers. Scale bar: 10 mm.

Representative R-curves Corresponding to the Thin-layer Mortar-SI and Mortar-PU systems

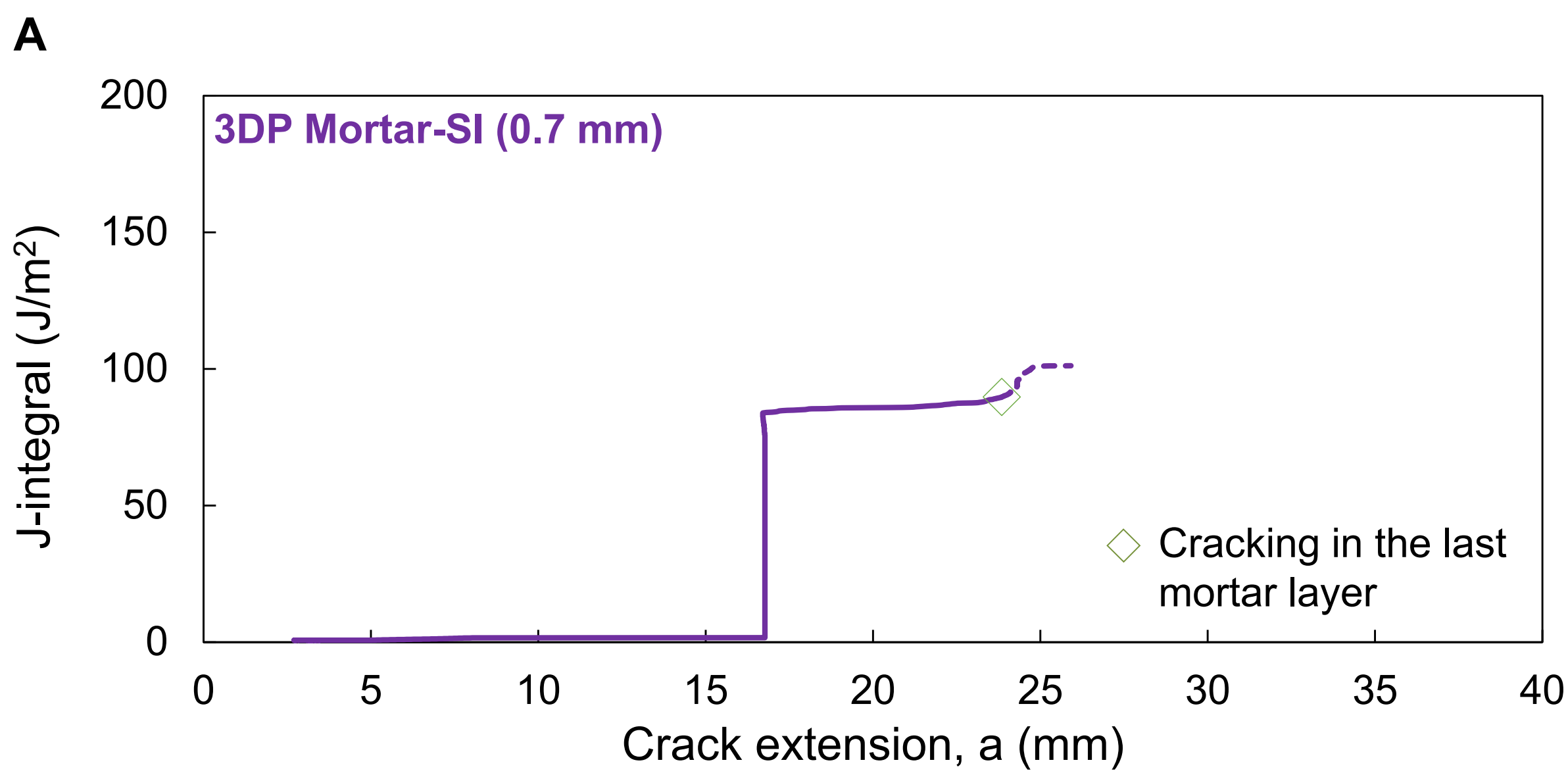

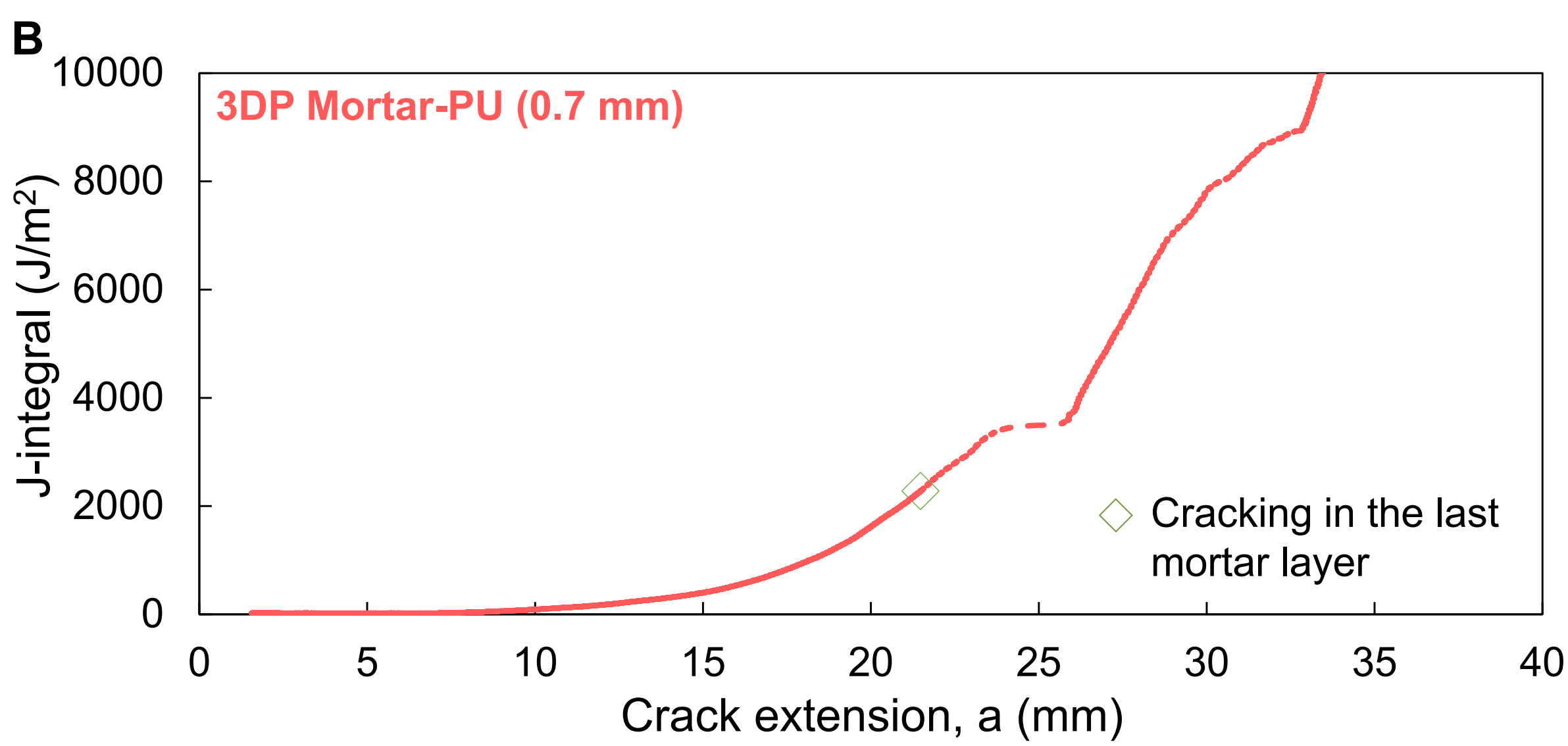

Figure S13: Representative R-curves (J-integral versus crack extension) for the two layered mortar-elastomer composites. A. 3DP Mortar-PU with $t_e = 0.7$ mm and B. 3DP Mortar-SI with $t_e = 0.7$ mm, obtained from SENB tests. For each specimen, the specific J-integral value reported in Figure 9B in the main manuscript corresponds to the point marked "Last Mortar Layer Cracking" on the R-curve, i.e., the final fracture event in the uppermost mortar layer before the remaining load is carried predominantly by elastomer bridging and interfacial processes. These representative curves illustrate the substantially higher fracture resistance and damage resilience in the Mortar-PU system, supported by and extended rising R-curve behavior, compared to the Mortar-SI counterpart.

Variability in Fracture Properties

The relatively large scatter observed in the specific J-integral for the layered mortar-elastomer composites arises primarily as a derived quantity, not a directly measured variable. As detailed in Supporting Information, J-integral depends on (i) the effective modulus of elasticity E_{eff}, extracted from the load-CMOD compliance of each specimen, and (ii) the load-CMOD up to the first peak. Both E_{eff} and the first-peak load themselves exhibit variability across nominally identical specimens, and this variability propagates directly into the computed J-integral. For example, the effective modulus of elasticity for the 3DP mortar-silicone system exhibits a standard deviation on the order of 40% of the mean, consistent with the scatter observed in specific J-integral.

This level of variation is physically reasonable for 3D-printed layered composites. Unlike monolithic mortars, the architected mortar-elastomer beams are true multi-material systems with multiple interfaces. Each additional design degree of freedom (constituent, interface, layer thickness, etc.) introduces an additional source of intrinsic scatter in the effective properties. Therefore, a higher variability relative to single-constituent monolithic mortars is expected. Moreover, the global compliance is highly sensitive to the soft layers: small specimen-to-specimen differences in elastomer thickness, interfacial quality, or local deformation response can have a pronounced effect on overall stiffness and therefore on the calculated J-integral.

Finally, fracture in these hard-soft layered systems proceeds through a multi-stage sequence of mechanisms - crack initiation in a mortar layer, arrest or deflection at the elastomer interlayer, crack bridging, and re-nucleation in subsequent mortar layers. The competition between these energetically distinct toughening mechanisms can be somewhat stochastic in that a difference in the local microstructure or interface conditions, however small, can shift the onset of crack arrest, re-nucleation, or bridging, altering, leading to noticeable variations in compliance, peak loads, and the displacement at which load drops or post-peak hardening occur. The sequential nucleation of cracks across multiple layers also introduces both statistical and energetic variability, akin to a Type 1 size-effect situation in which failure is governed by crack initiation at the weakest location in a heterogeneous structure (i.e., mortar layers in the case of proposed composites). In this context, the error bars can be interpreted as reflecting the intrinsic stochasticity of the architected composite, however, further quantification of the distributions and uncertainties is a worthwhile future effort. Importantly, despite such variability, the layered mortar-elastomer, in the case of PU-based systems, consistently and reproducibly exhibits an order-of-magnitude enhancement in specific J-integral and ductility relative to their monolithic mortar counterparts, making the effect of variability less significant.

Acoustic Emission Activity Corresponding to the Thin-layer Mortar-SI and Mortar-PU systems

To complement the load-CMOD, R-curve, and DIC analyses, in-situ acoustic emission (AE) measurements were performed during SENB tests on representative 3DP Mortar-Silicone and Mortar-Polyurethane layered composites with $t_e = 0.7$ mm. The AE signals were recorded using broadband sensors and processed to extract standard hit-level metrics (counts, energy, amplitude) as a function of time and localized position along the beam length.

Figure S14 and S15 summarize the temporal evolution of AE counts and energy, overlaid with the force-time response, for the Mortar-SI and Mortar-PU composites, respectively. In the Mortar-SI system (Figure S14), AE activity is strongly concentrated in a narrow time window around peak load: high-count, high-energy bursts coincide with the dominant mortar-layer fracture episode, followed by sparse activity during the elastomer-dominated bridging regime. By contrast, the Mortar-PU system (Figure S15) exhibits sustained AE activity that persists well into the post-peak regime, indicating a more prolonged sequence of damage events (layer-wise mortar cracking, interfacial dissipation, and soft-layer participation) during stable crack growth.

Figure S16 and S17 further analyze AE amplitude in conjunction with cumulative counts and cumulative energy. For Mortar-SI (Figure S16), both cumulative metrics show steep step-wise increases localized around a single high-amplitude cluster, consistent with a relatively short, localized fracture process. For Mortar-PU (Figure S17), cumulative counts and energy grow in multiple stages over an extended time interval, reflecting continued energy dissipation beyond the first major cracking event and supporting the interpretation of a more distributed and temporally extended damage evolution in the stiffer-elastomer system.

Finally, Figure S18 and S19 provide one-dimensional AE source-localization along the beam length for the Mortar-SI and Mortar-PU composites, respectively. In the mortar-SI composite (Figure S18), both event amplitude and event count are strongly localized near the notch/midspan region, consistent with a comparatively concentrated fracture process. In the mortar-PU composite (Figure S19), the spatial distribution of AE activity is broader around the central fracture region, indicative of more distributed damage and layer-wise cracking coupled with interface/soft-layer dissipation. Together, Figure S14-S19 corroborate the multi-stage, spatially and temporally extended damage mechanisms in the architected hard–soft composites - particularly in the Mortar-PU system - and reinforce the mechanistic picture underlying the rising R-curves and large fracture toughness, load-bearing, and ductility enhancements.

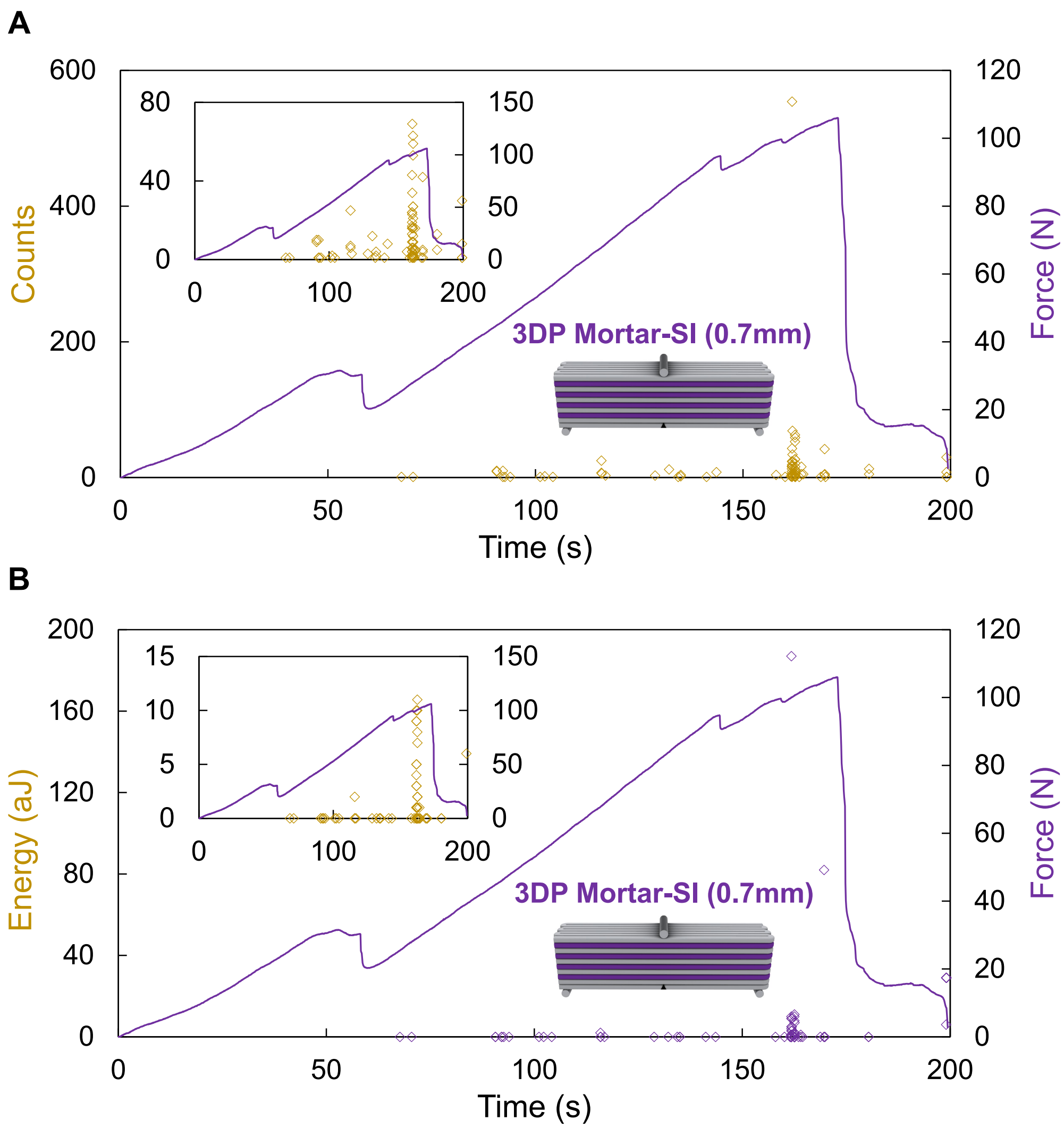

Figure S14: Event-level AE signal metrics, counts and energy (left Y-axes), overlaid with continuous force-time plot (right Y-axis), for the 3DP Mortar-Silicone layered composite (t_e = 0.7 mm) during SENB test. A. AE *counts* versus time (left axis), demonstrates that high-count events localize within a narrow time window near peak load, consistent with burst-like cracking of mortar layers, followed by sparse activity during elastomer-dominated bridging. B. AE *energy (aJ)* (left axis) versus time, illustrates that the highest-energy events coincide with the main fracture episode while lower-energy events persist at later times. Insets magnify the peak-load window to emphasize the clustering of high-count/high-energy events associated with the dominant mortar-layer cracking stage.

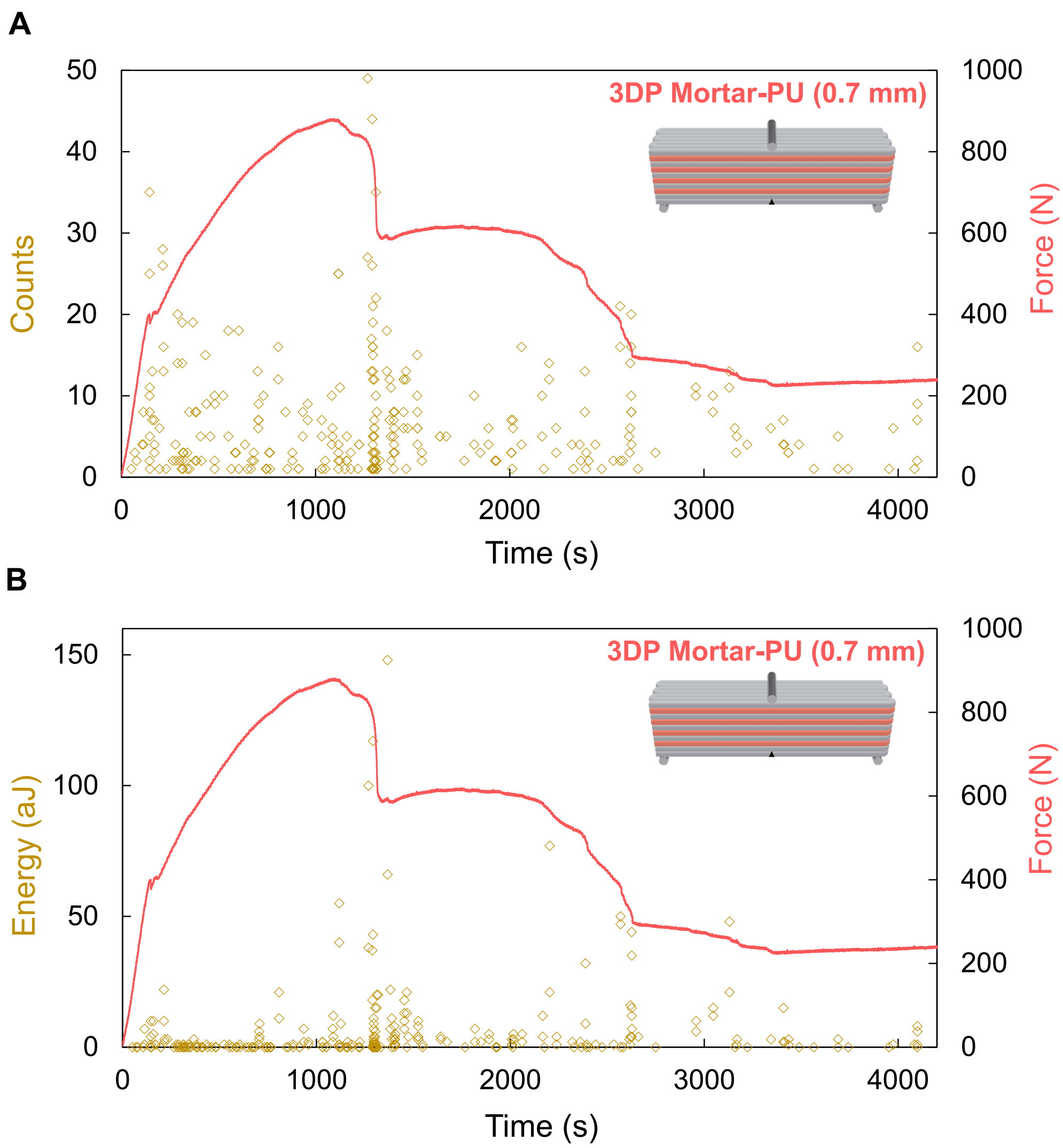

Figure S15: Event-level AE signal metrics, counts and energy (left Y-axes), overlaid with continuous force-time plot (right Y-axis), for the 3DP Mortar-Polyurethane layered composite (t_e = 0.7 mm) during SENB test. A. Event *counts* versus time (left axis), demonstrates sustained AE activity distributed throughout loading and into the post-peak regime, consistent with progressive damage processes (layer-wise mortar cracking coupled with interface/PU-mediated dissipation) rather than a sparse or single burst around the peak. B. Event *energy (aJ)* (left axis) versus time, illustrates that energetic events occur not only near the first major load drop but also during subsequent stages as the composite continues to carry load, consistent with ongoing dissipation during stable crack growth and interfacial/soft-layer participation.

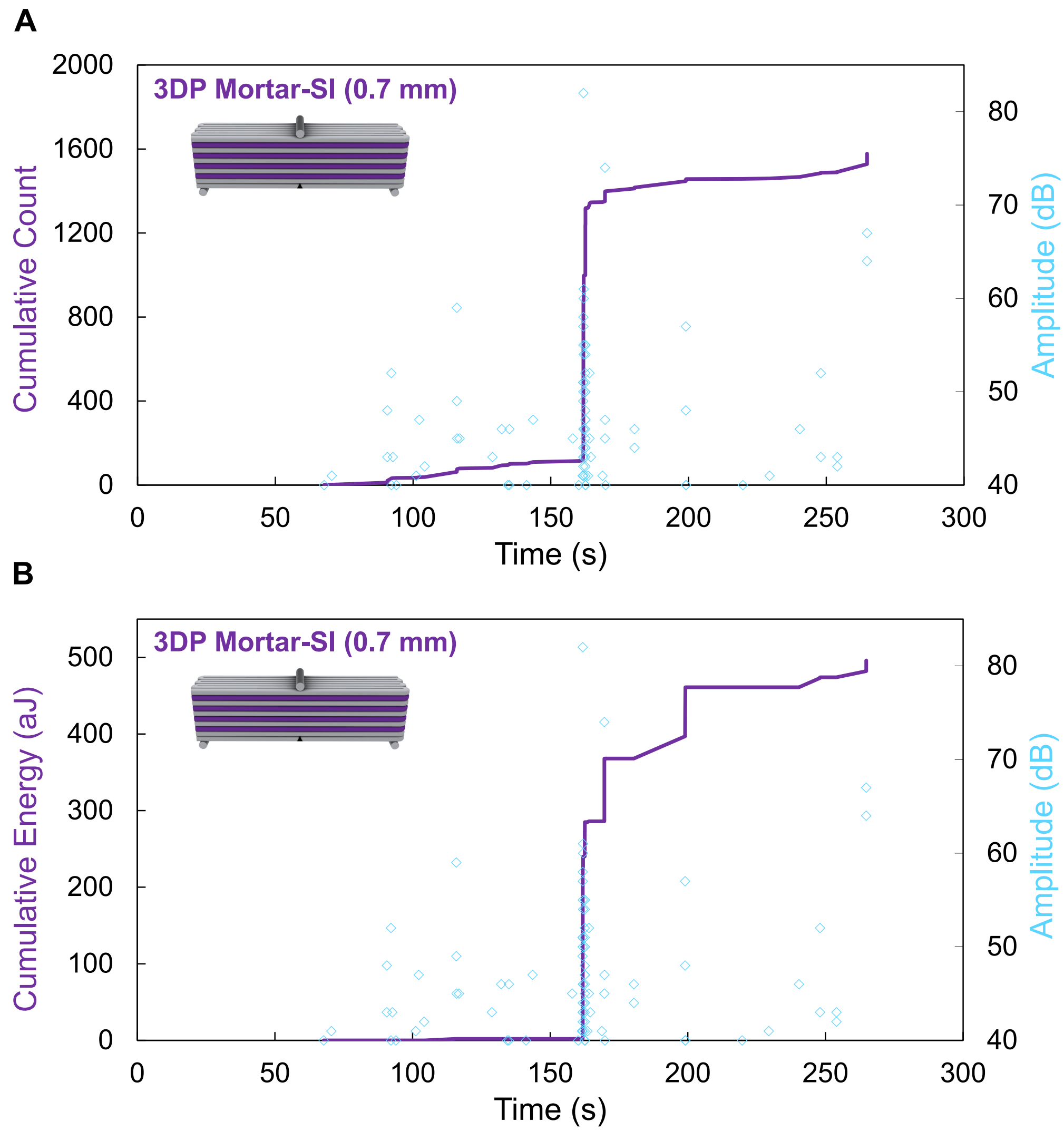

Figure S16: AE amplitude evolution with cumulative metrics, counts and energy, for the 3DP Mortar-Silicone layered composite ($t_e = 0.7$ mm) during SENB test. Diamond markers denote the amplitude (dB) of individual AE events (right axis), overlaid with cumulative count or cumulative energy (left axes) to visualize when the dominant emission activity occurs. A. Cumulative AE *event count* (solid curve, left axis) versus time with superimposed event amplitude (right axis), showing step-wise accumulation concentrated around the principal cracking episode (amplitude burst), followed by a near-plateau indicating limited additional damage or microcracking once silicone-dominated bridging governs deformation. B. Cumulative AE *energy (aJ)* (solid curve, left axis) versus time with superimposed event amplitude (right axis), demonstrating that most cumulative energy is accrued during the same narrow high-amplitude window, consistent with localized mortar-layer fracture events dominating the acoustic signature in the silicone-based system.

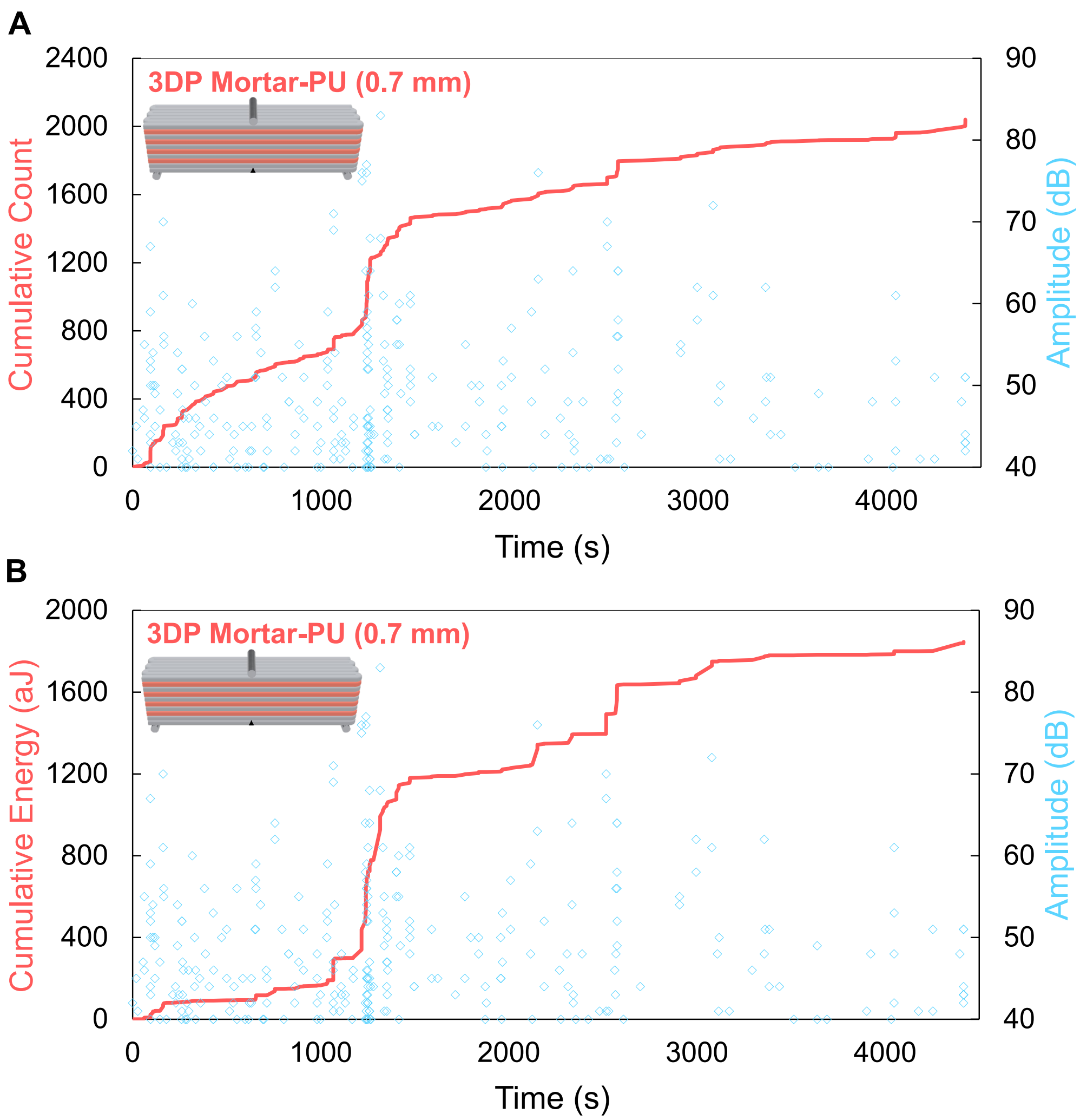

Figure S17: AE amplitude evolution with cumulative metrics, counts and energy, for the 3DP Mortar-Polyurethane layered composite ($t_e = 0.7$ mm) during SENB test. Diamond markers denote the amplitude (dB) of individual AE events (right axis), overlaid with cumulative metrics (left axis) to characterize the prolonged damage evolution enabled by the stiffer elastomer. A. Cumulative AE *event count* versus time (solid curve, left axis) with event amplitude (right axis), highlighting a progressive (multi-step) accumulation throughout loading and well into the post-peak regime, consistent with sustained emission activity during stable crack growth and interface/PU-governed mechanisms. B. Cumulative AE *energy* (aJ; solid curve, left axis) with event amplitude (right axis), showing continued energy accumulation beyond the first major mortar cracking episode, supporting that the PU-based architecture maintains active dissipation pathways as load is redistributed through the layered system during extended deformation.

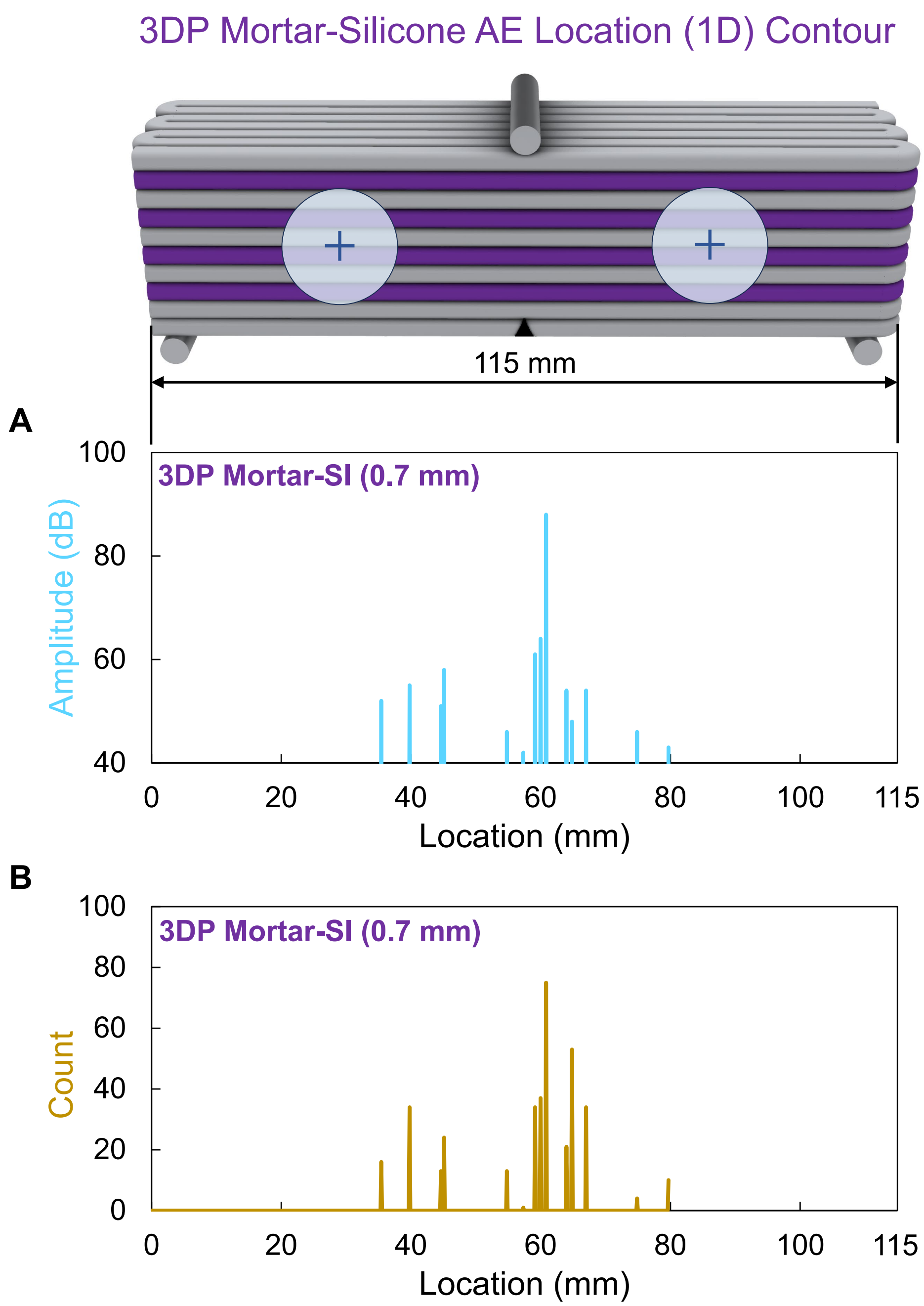

Figure S18: One-dimensional (1D) AE source-location distribution along the beam length for the 3DP Mortar-SI layered composite ($t_e = 0.7$ mm) during SENB test. A schematic indicates the specimen length (115 mm) and the 1D coordinate used for localization with two sensors mounted to one side. A. The 1D distribution of event *amplitude* (dB) versus location, and B. The corresponding event *count* versus location. The AE activity is strongly concentrated near the middle one-third region, where the dominant experimentally observed crack path and toughening mechanisms reside, indicating that acoustic activities remains spatially localized in the mortar-silicone composite.

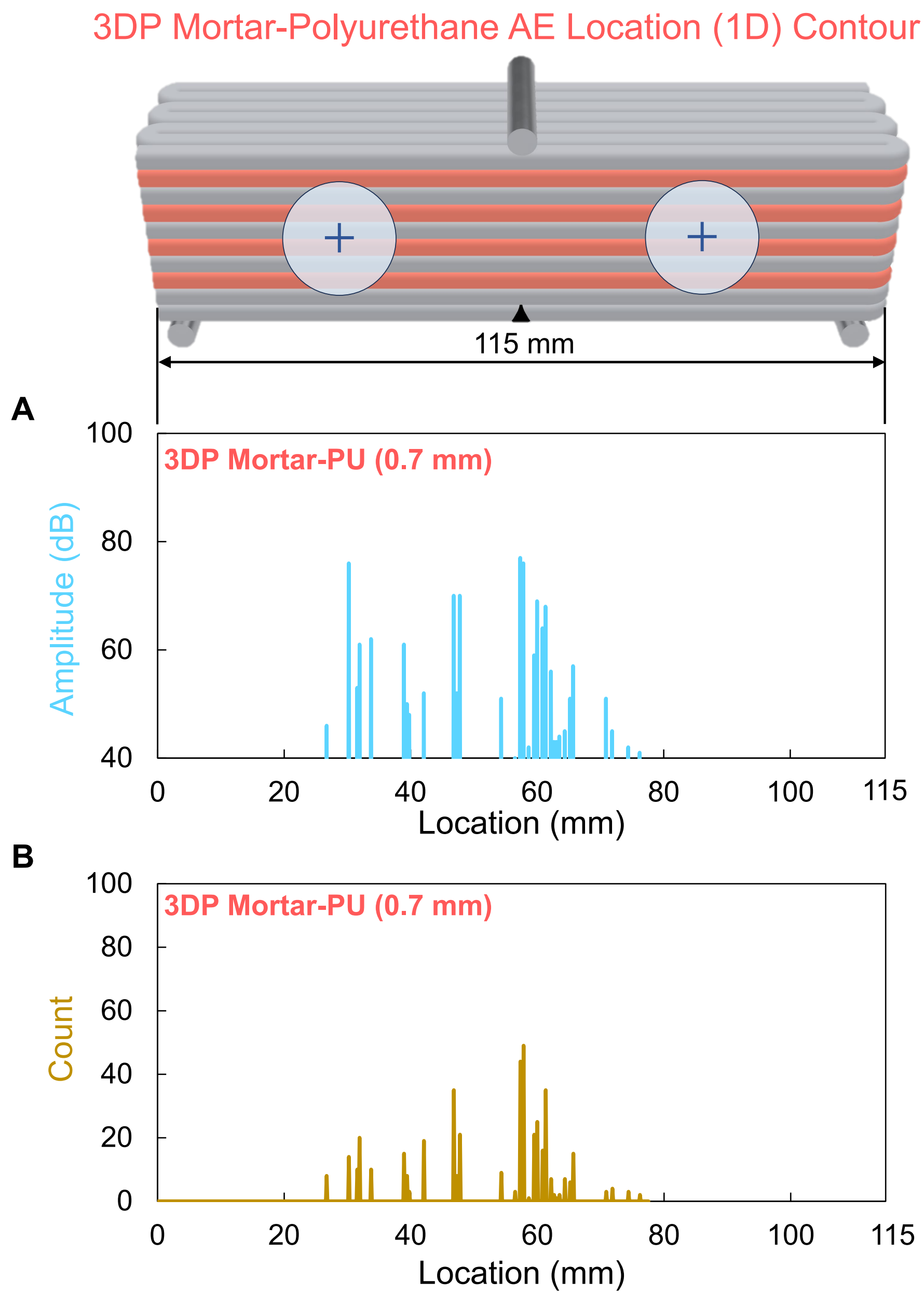

Figure S19: One-dimensional (1D) AE source-location distribution along the beam length for the 3DP Mortar–PU layered composite ($t_e = 0.7$ **mm) during the SENB test.** A schematic indicates the specimen length (115 mm), the 1D coordinate system used for localization, and the positions of the two AE sensors mounted on one side of the beam. A. One-dimensional distribution of event *amplitude* (dB) versus location. B. Corresponding distribution of event *count* versus location. Compared to the Mortar-SI composite, the mortar-PU architecture exhibits a broader spatial distribution of AE activity around the central region, where the dominant crack path and toughening mechanisms are experimentally observed. This broader spread is indicative of a more extended damage process zone associated with layer-wise mortar cracking and interface/soft-layer dissipation in the multi-material medium.

Supporting Videos

- **Movie S1**: Multi-material Additive Manufacturing (MMAM) Technique with Concrete-Silicone (Si: 2.7 mm) (3X) (Link - https://youtu.be/zs7Dg7e40F4)
- **Movie S2**: Multi-material Additive Manufacturing (MMAM) Technique with Concrete-Polyurethane (PU: 0.7 mm) (3X) (Link - https://youtu.be/o5v62SogYEw)
- **Movie S3**: Von Mises Strain in Mortar-Polyurethane Additively Manufactured Composites Under SENB test (Link - https://www.youtube.com/watch?v=QwcpEzYK83g)
- **Movie S4**: Shear Strain in Mortar-Polyurethane Additively Manufactured Composites Under SENB test (Link - https://www.youtube.com/watch?v=WzQBQZaGCSs)

References

[1] E3D-Online, Research and development: Motion system and tool-changer, **2018**, Accessed: 2025-08-08.

[2] S. M. Sajadi, P. J. Boul, C. Thaemlitz, A. K. Meiyazhagan, A. B. Puthirath, C. S. Tiwary, M. M. Rahman, P. M. Ajayan, *Advanced Engineering Materials* **2019**, *21*, 8 1801380.

[3] T. Ching, Y. Li, R. Karyappa, A. Ohno, Y.-C. Toh, M. Hashimoto, *Sensors and Actuators B: Chemical* **2019**, *297* 126609.

[4] H. Gonabadi, A. Yadav, S. Bull, *The International Journal of Advanced Manufacturing Technology* **2020**, *111*, 3 695.

[5] Duet3D Documentation, Reprapfirmware overview, **2024**, Accessed: 2025-08-08.

[6] Duet3D Documentation, Duex2 and duex5, **2024**, Accessed: 2025-08-08.

[7] ASTM International, Standard test method for measurement of fracture toughness, Technical Report E1820-22, ASTM International, West Conshohocken, PA, **2022**, URL https://www.astm.org, ASTM E1820-22.

[8] Y. Jenq, S. P. Shah, *Engineering fracture mechanics* **1985**, *21*, 5 1055.

[9] N. Banthia, J. Sheng, *Cement and Concrete Composites* **1996**, *18*, 4 251.

[10] N. Banthia, N. Nandakumar, *Cement and Concrete Composites* **2003**, *25*, 1 3.

[11] A. Najmeddine, S. Gupta, R. Moini, *Journal of the Mechanics and Physics of Solids* **2025**, *196* 106016.

[12] L. Ambrosio, V. M. Tortorelli, *Communications on Pure and Applied Mathematics* **1990**, *43*, 8 999.

[13] K. Park, G. H. Paulino, J. R. Roesler, *Journal of the Mechanics and Physics of Solids* **2009**, *57*, 6 891.

[14] R. N. Swamy, *Cement and Concrete Research* **1971**, *1*, 5 559.

[15] H. W. Greensmith, A. Thomas, *Journal of polymer science* **1955**, *18*, 88 189.

[16] ASTM International, Standard test method for tear strength of conventional vulcanized rubber and thermoplastic elastomers, Technical Report D624-00(2012), ASTM International, West Conshohocken, PA, **2012**, URL https://www.astm.org/d624-00r12.html, ASTM D624-00(2012).

[17] ASTM International, Standard test methods for vulcanized rubber and thermoplastic elastomers—tension, Technical Report D412-16(2021), ASTM International, West Conshohocken, PA, **2021**, URL https://www.astm.org/d412-16r21.html, ASTM D412-16(2021).

[18] M. Shakiba, A. Najmeddine, *Journal of Mechanics of Materials and Structures* **2023**, *17*, 3 229.

[19] A. Prihar, S. Gupta, H. S. Esmaeeli, R. Moini, *Nature Communications* **2024**, *15*, 1 7498.

[20] E. Theotokoglou, E. Sideridis, *Journal of reinforced plastics and composites* **2011**, *30*, 13 1125.

[21] S. R. Choi, D. Zhu, R. Miller, *Engineering fracture mechanics* **2005**, *72*, 13 2144.
